# Genome‑wide identification, phylogenetic and expression pattern analysis of GATA family genes in foxtail millet (*Setaria italica*)

**DOI:** 10.1186/s12864-022-08786-0

**Published:** 2022-08-02

**Authors:** Dili Lai, Xin Yao, Jun Yan, Anjing Gao, Hao Yang, Dabing Xiang, Jingjun Ruan, Yu Fan, Jianping Cheng

**Affiliations:** 1 College of Agriculture, Guizhou University, Guiyang City, 550025 People’s Republic of China; 2 School of Food and Biological Engineering, Chengdu University, Chengdu, 610106 People’s Republic of China

**Keywords:** *Setaria italica*, GATA, Genome-wide, Abiotic stress

## Abstract

**Background:**

Transcription factors (TFs) play important roles in plants. Among the major TFs, GATA plays a crucial role in plant development, growth, and stress responses. However, there have been few studies on the GATA gene family in foxtail millet (*Setaria italica*). The release of the foxtail millet reference genome presents an opportunity for the genome-wide characterization of these GATA genes.

**Results:**

In this study, we identified 28 GATA genes in foxtail millet distributed on seven chromosomes. According to the classification method of GATA members in *Arabidopsis*, *SiGATA* was divided into four subfamilies, namely subfamilies I, II, III, and IV. Structural analysis of the *SiGATA* genes showed that subfamily III had more introns than other subfamilies, and a large number of cis-acting elements were abundant in the promoter region of the *SiGATA* genes. Three tandem duplications and five segmental duplications were found among *SiGATA* genes. Tissue-specific results showed that the *SiGATA* genes were mainly expressed in foxtail millet leaves, followed by peels and seeds. Many genes were significantly induced under the eight abiotic stresses, such as *SiGATA10*, *SiGATA16*, *SiGATA18,* and *SiGATA25*, which deserve further attention.

**Conclusions:**

Collectively, these findings will be helpful for further in-depth studies of the biological function of SiGATA, and will provide a reference for the future molecular breeding of foxtail millet.

**Supplementary Information:**

The online version contains supplementary material available at 10.1186/s12864-022-08786-0.

## Introduction

Transcription factors (TFs) regulate gene expression by recognizing and binding to cis-acting elements in the promoter regions of target genes [1]. These proteins play important roles in plants, including controlling flower development [2, 3], carbon and nitrogen metabolism [4], the circadian clock [5], cell differentiation [6], hormone response [7], and disease resistance [8]. At present, many TFs have been identified and analyzed in foxtail millet, such as NAC [9], bHLH [10], AP2/ERF [11], GRAS [12], WRKY [13], and bZIP [14]. However, few studies have focused on the GATA TF family in foxtail millet. As its name suggests, the GATA TFs can recognize and bind to the W-GATA-R (W = T/A, R = G/A) domain in the promoter region and regulate the transcription level of downstream genes [15, 16]. The DNA domain of these GATA TFs were composed of a type IV zinc finger in the form of CX_2_CX_17-20_CX_2_C, followed by a highly basic region [17]. Most animal and fungal GATA factors contain the CX_2_CX_17_CX_2_C or CX_2_CX_18_CX_2_C domains [18, 19]. Plant GATA factors typically contain 17–20 residues in the zinc finger, whereas the vast majority of GATA TF in plants contain (CX_2_CX_18_CX_2_C) and (CX_2_CX_20_CX_2_C) zinc finger structures [16, 20].

Studies have shown that the interaction between the zinc finger and specific DNA elements were promoted by hydrophobic interactions with nitrogen groups in the DNA main groove [21, 22]. In plants, GATA TFs are considered important regulators of many biological processes, such as stress responses, nitrogen metabolism, flowering, development, and hormone signal transduction [17, 23]. In *Arabidopsis thaliana*, the overexpression and loss of function of GATA TFs GNC (GATA factor, nitrate-inducible, carbon metabolism-involved) and GNL (GNC-Like) alter the regulation of chlorophyll synthesis, flowering time, and cold tolerance [24–26]. GNC and GNL are important inhibitors of the gibberellin signaling pathway that function through DELLA and PIF regulation [27, 28]. In wheat, overexpression of the GATA TF *TaZIM-A1* results in delayed flowering and reduced 1000-grain weight [29]. GATA TFs also play an important role in plants in response to abiotic stress. For example, the light efficiency and biomass of rice *OsGATA8*-overexpressing lines under salt stress are higher than those of wild-type and mutant plants [30]. Under low nitrogen stress, the expression levels of *GATA44* and *GATA58* in soybean seedlings are significantly reduced [31]. The first GATA transcription factor in plants was identified in tobacco, in which researchers cloned a GATA-1 zinc finger protein homologous to a fungal nitrogen metabolism regulator. At present, GATA TFs have been identified in many plants, including *Arabidopsis* [17], rice [17, 23], soybean [31], *Brachypodium distachyon* [32], tomato [33], maize [34], potato [35], and rape [36].

Foxtail millet, a model plant for Poaceae C4, originated in northern Asia [37–39]. The identification, classification, evolution, and function of the GATA gene family are not clear at present. Therefore, in this study, the structures, cis-acting elements, duplication events, and predicted protein–protein interactions of 28 GATA genes in the whole genome of foxtail millet were analyzed. We also discuss the evolutionary relationships of these *SiGATA* genes among several plants, including *A. thaliana*, tomato, soybean, *B. distachyon*, rice, and maize, and analyze the conserved motifs, collinearity, and evolutionary relationship between the *SiGATA* and GATA genes in other plants. In addition, the spatial and tissue expression patterns of *SiGATA* genes in different tissues during millet fruit development were analyzed to determine the role of specific *SiGATA* members in different biological processes of foxtail millet development. The expression of *SiGATA* genes in foxtail millet seedlings under eight types of abiotic stress was measured at different times after treatment, and the response of different *SiGATA* genes to stresses were determined. In this study, we comprehensively analyzed the GATA gene family of foxtail millet and screened important GATA genes for foxtail millet during growth and development processes and under stress treatment, providing a reference for the molecular breeding of foxtail millet.

## Results

### Identification of GATA genes in foxtail millet

In total, 28 GATA genes in the whole genome of foxtail millet were identified using two BLAST methods (Table S1), and these were renamed *SiGATA01* to *SiGATA28* according to their distribution sequence on millet chromosomes. The basic characteristics of these genes were analyzed, including coding sequence (CDS) length, protein sequence length, relative molecular weight, isoelectric point, and subcellular localization prediction (Table S1). Among the 28 SiGATAs, the shortest protein sequence was SiGATA17 with only 141 amino acids. The longest protein sequence was SiGATA26, comprising 580 amino acids. In general, the shorter the amino acid sequence, the smaller the relative molecular weight of the protein. The relative molecular weight of the 28 SiGATA proteins in this study ranged from 15.36 KDa (SiGATA17) to 61.68 KDa (SiGATA26). The isoelectric points of the 28 SiGATA proteins varied widely, ranging from 4.77 (SiGATA24) to 10.18 (SiGATA21). Interestingly, the isoelectric points of most SiGATA proteins (22/28) were greater than 7, suggesting that SiGATA proteins were biased towards being rich in basic amino acids. Subcellular localization prediction showed that 21 SiGATA proteins were localized in the nucleus, followed by five in the chloroplast, with one each in the mitochondria and plastids.

### Multiple sequence alignment, phylogenetic analysis, and classification of SiGATA proteins

**Fig. 1:**
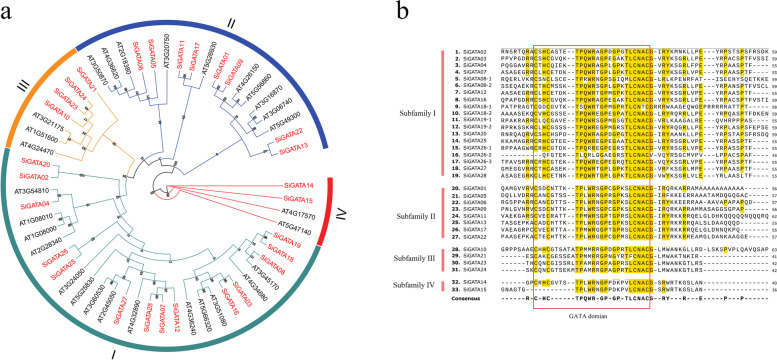
Unrooted phylogenetic tree and sequence alignment showing the relationship between GATA proteins of *Setaria italica and Arabidopsis thaliana.* The phylogenetic tree was generated using the ML method with MEGA X. **a** The phylogenetic relationship between *S. italica* and *A. thaliana* GATA protein was studied. **b** Approximately 60 bp sequence alignment of the *SiGATA* domain

To explore the evolutionary relationship of the GATA protein family in foxtail millet, the amino acid sequences of 28 SiGATA proteins and 29 *A. thaliana* GATA proteins were used to construct a phylogenetic tree (Table S2). According to the classification method of GATA proteins previously reported in *A. thaliana* [17], 28 foxtail GATA proteins were divided into four subfamilies, namely subfamilies I, II, III, and IV. Among the four subfamilies, subfamily I contained the most members with 14 SiGATA proteins. This was followed by subfamily II (8), subfamily III (4), and subfamily IV, with only two SiGATA proteins (SiGATA14 and SiGATA15) (Fig. 1a, Table S1). Some SiGATA proteins were found to cluster closely to *A. thaliana* GATA proteins (bootstrap support ≥ 70). These proteins might be homologous and have similar physiological functions.

To further understand the domains of GATA in different subfamilies, we extracted and compared the amino acid sequences of 28 GATA protein domains. Most SiGATA proteins contained only one GATA domain, whereas SiGATA8, SiGATA18, and SiGATA19 had two GATA domains, and SiGATA26 had three. As shown in Fig. 1b, the SiGATA proteins contained conserved GATA domain sequences. Interestingly, all members of subfamilies I and II contained 18 residues in the zinc finger loop (CX_2_CX_18_CX_2_C), whereas all members of subfamily III contained 20 residues in the zinc finger loop (CX_2_CX_20_CX_2_C). In subfamily IV, SiGATA14 had a typical CX_2_CX_18_CX_2_C domain structure. However, SiGATA15 lacked the first CX_2_C structure, which might give rise to new functions for SiGATA15. In addition, the GATA domains of all subfamilies also contained highly conserved amino acid sites, such as TP, GP, and LCNACG. However, differences were observed in the GATA domains among the different subfamilies to some extent. For example, different subfamilies showed abundant variability in an amino acid site before the conserved region of LCNACG, with T for subfamily I and III, S for subfamily II, and V for subfamily IV. Variations at these loci could enable different subfamilies to perform different functions.

### Gene structures, conserved motifs, and cis‑acting elements analysis of *SiGATA* gene family

**Fig. 2:**
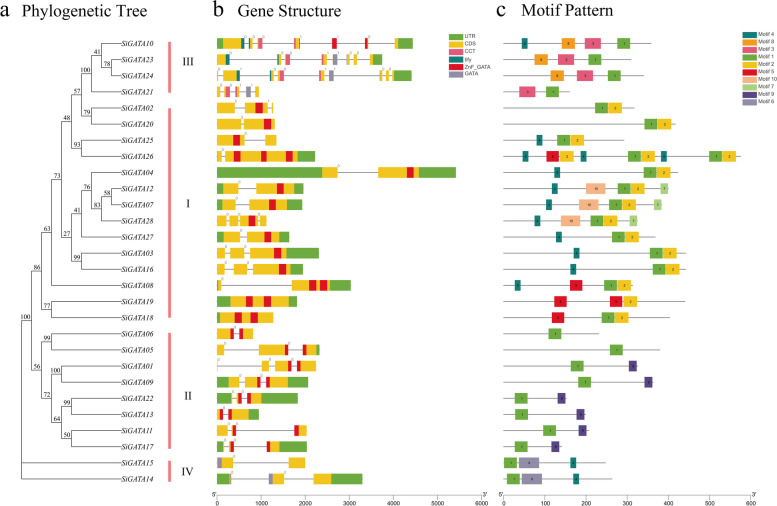
Phylogenetic relationships, gene structure, and motif distributions of *S. italica* GATA genes. **a** The phylogenetic tree was constructed with the ML method with 1000 replicates on each node. **b** Exons and introns are indicated by rectangles and gray lines, respectively. **c** The amino acid motifs (1–10) in SiGATA protein are shown in colored boxes. The black line shows the relative lengths of proteins

Many differences in the number and distribution of introns in different subfamilies were observed, with the number of introns ranging from zero to eight. Subfamily III had the largest number of introns (Fig. 2, Table S1), with an average of 6.25 introns, of which *SiGATA23* and *SiGATA24* contained seven and eight introns, respectively. Subfamily I had the least number of introns, with *SiGATA18* and *SiGATA19* containing no introns. Genes in the same subfamily had similar intron/exon structures. Further analysis of the distribution of GATA domains showed diversity in the distribution of domains of different subfamilies. All member domains of subfamily I were found to be distributed on a single exon and were not separated by introns. This was true for genes with only one, two (*SiGATA08*, *SiGATA18*, *SiGATA19*), or three (*SiGATA26*) GATA domains. Interestingly, this was not the case for the domain distribution of subfamily II members. The GATA domains of these genes contained introns that require further post-transcriptional modifications to eliminate introns. These results are similar to those observed in the rice GATA TF family [23]. In addition, some *SiGATA* genes were found to have CCT or tify domains in addition to the GATA domains. For example, members of subfamily III had one or both CCT and tify domains. The structural diagram of all SiGATA proteins was constructed using the online MEME tool. As shown in Fig. 2c, SiGATA members of the same subfamily tended to have similar motifs, except for conserved motif 1 of the widely distributed GATA domain (Table S3). Conservative motifs 8 and 3 were unique to subfamily III; 5,10, and 2 were unique to subfamily I; 9 was unique to subfamily II, and 6 was unique to subfamily IV. The similar motif arrangements of the SiGATA proteins in the same subfamily indicated that their protein structure was conserved. In conclusion, similar gene structures, conserved motif arrangements, and phylogenetic tree structures within the same subfamily further support the reliability of the SiGATA subfamily classification.

In total, 91 cis-acting elements were found in the promoter region of the foxtail millet GATA family by analyzing 2 KB promoter sequences upstream of the *SiGATA* initiation codon coding sequence (ATG). The promoter regions of the foxtail millet GATA family were found to contain abundant cis-acting elements, falling into seven categories as follows: light response, hormone response, promoter-related, developmental-related, environmental-stress-related, binding-site-related, and other elements (Table S4). All *SiGATA* genes contained core elements related to transcription initiation (TATA-box and CAAT-box), which proves that the promoter analysis was reliable. Among the environmental stress-related elements, hypoxia-inducible (GC-motif, ARE), low-temperature response (LTR), and drought-inducible (MBS) elements were found to be widely present in the *SiGATA* genes. Two hormone-related elements were found in most *SiGATA* genes, abscisic acid cis-acting element (ABRE) and methyl jasmonate cis-acting elements (TGACG-motif and CGTCA-motif). Most genes contained the light response-related elements G-Box (CACGTC), Box 4 (ATTAAT), and Sp1 (GGGCGG), which are involved in light response regulation. Root expression-related elements (AS-1), endosperm expression-related elements (AAGAA-motif), and meristem expression-related elements (CAT-box) are development-related elements, which were also determined to exist in most GATA genes. In addition, 26 other types of cis-acting elements were found in the foxtail millet GATA promoter regions, including Unnamed_4, STRE, MYB, and MYC. Venn analysis was performed on cis-acting elements of more than 10 *SiGATA* genes. Among the elements related to environmental stress (Figure S1a), *SiGATA17*, *SiGATA03*, and *SiGATA21* all had five cis-acting elements (ARE, LTR, MBS, GC- motif, and W box). Three genes (*SiGATA06*, *SiGATA26*, and *SiGATA02*) contained five developmentally relevant elements (Figure S1b). Twenty-two foxtail millet GATA genes contained an ABRE and a methyl jasmonate cis-acting element (CGGTA-motif and TGACG-motif) (Figure S1c). All millet GATA genes contained CAAT-box and TATA-box elements (Figure S1d). Among the light-responsive elements (Figure S1e), three genes (*SiGATA28*, *SiGATA09*, and *SiGATA12*) had the following five light-responsive cis-acting elements: GATA-motif, G-box, Box 4, GT1-motif, and TCCC-motif.

### Chromosomal distribution, gene duplication, and synteny analysis of the SiGATA genes

**Fig. 3:**
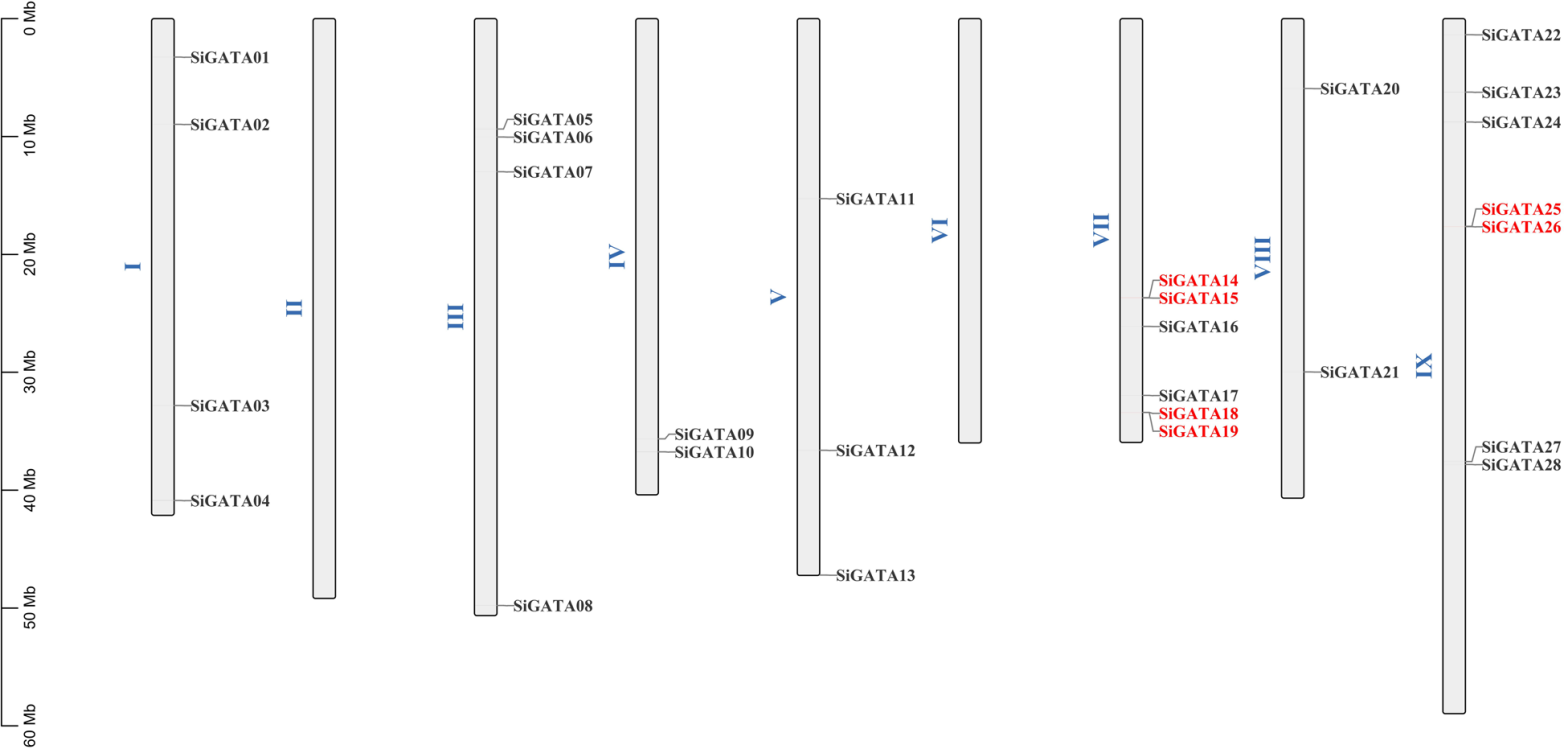
Schematic representation of the chromosomal distribution of *Setaria italica* GATA genes. Vertical bars represent the chromosome of *S. italica*. The chromosome number is on the left side of each chromosome. The scale on the left represents chromosome length. The red font represents the tandem gene duplication

**Fig. 4:**
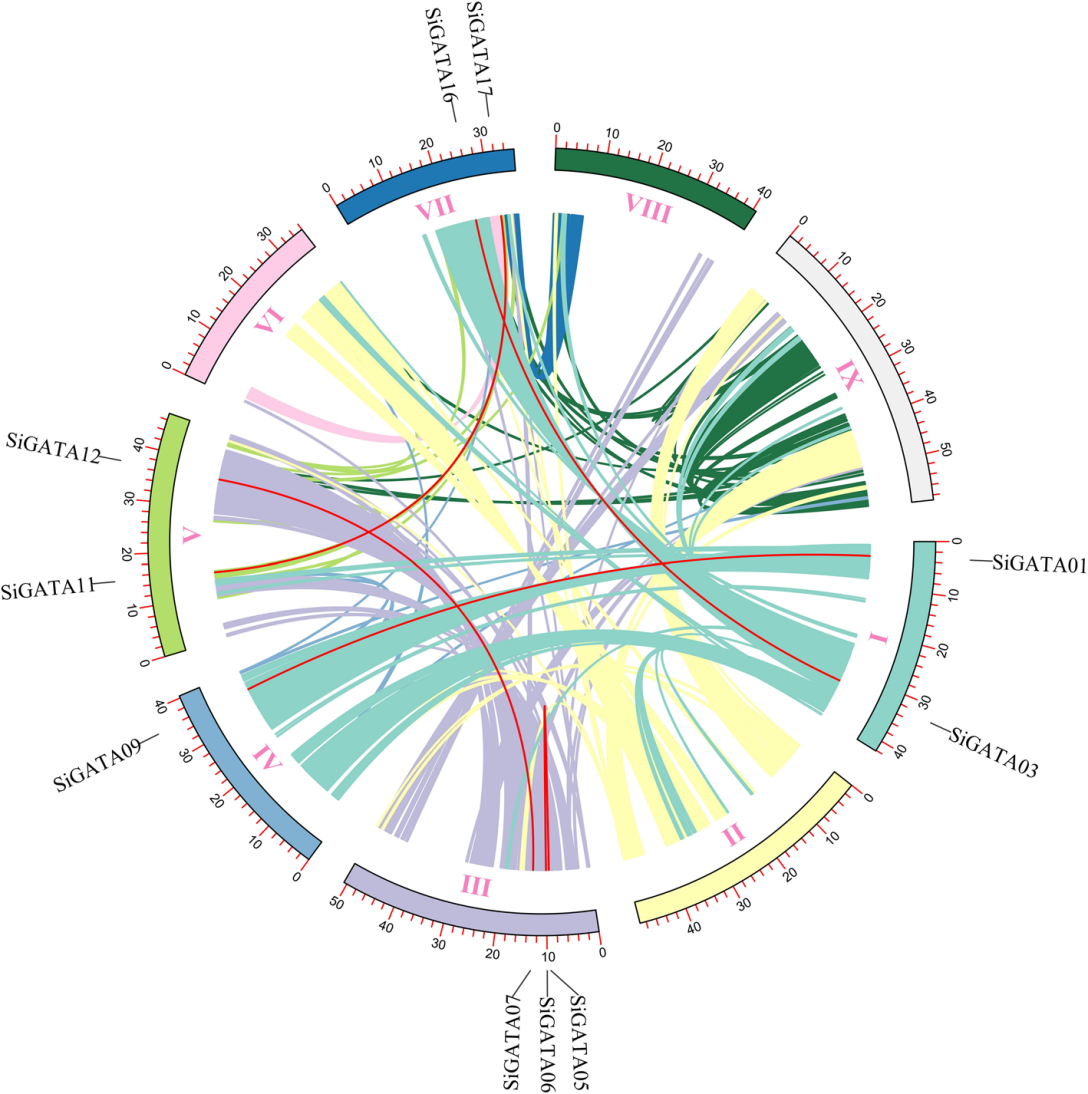
Colinear region of the GATA gene of *Setaria italica*. The colored lines represent all the colinear blocks in the *S. italica* genome, and the red lines represent GATA gene pairs subjected to segmental duplication. Chromosome numbers are shown at the bottom of each chromosome

According to the foxtail millet genome annotation, 28 *SiGATA* genes were mapped to nine chromosomes. As shown in Fig. 3, *SiGATA01* to *SiGATA28* were found to be distributed from chromosome I to chromosome IX. *SiGATA* genes were found in all chromosomes, except chromosomes II and VI. Among them, chromosome IX contained the most GATA genes (up to seven). Chromosomes IV and VIII contained only two GATA genes. According to Holub [40], two or more closely related genes distributed in the range of 200 KB were defined as tandem replication events. In the *SiGATA* genes, we found three tandem duplication events, including two on chromosome VII, namely *SiGATA14* and *SiGATA15*, *SiGATA18*, and *SiGATA19*, and one on chromosome IX, namely *SiGATA25* and *SiGATA26* (Table S5). In addition, segmental duplication events were identified using BLASTP and MCScanX (Fig. 4, Table S5). The genes with a fragment replication event included *SiGATA03*/*SiGATA16*, *SiGATA11*/*SiGATA17*, *SiGATA07*/*SiGATA12*, *SiGATA01*/*SiGATA09*, and *SiGATA05*/*SiGATA06*. Among the eight pairs of genes with duplication events, four pairs of *SiGATA* genes belonged to subfamily I, three pairs belonged to subfamily II, and one pair belonged to subfamily IV (Table S5). These duplication events are the main driving force of *SiGATA* genes expansion, and subfamily I and subfamily II, with a relatively large number of *SiGATA* genes, might have been expanded during the whole genome duplication process.

**Fig. 5:**
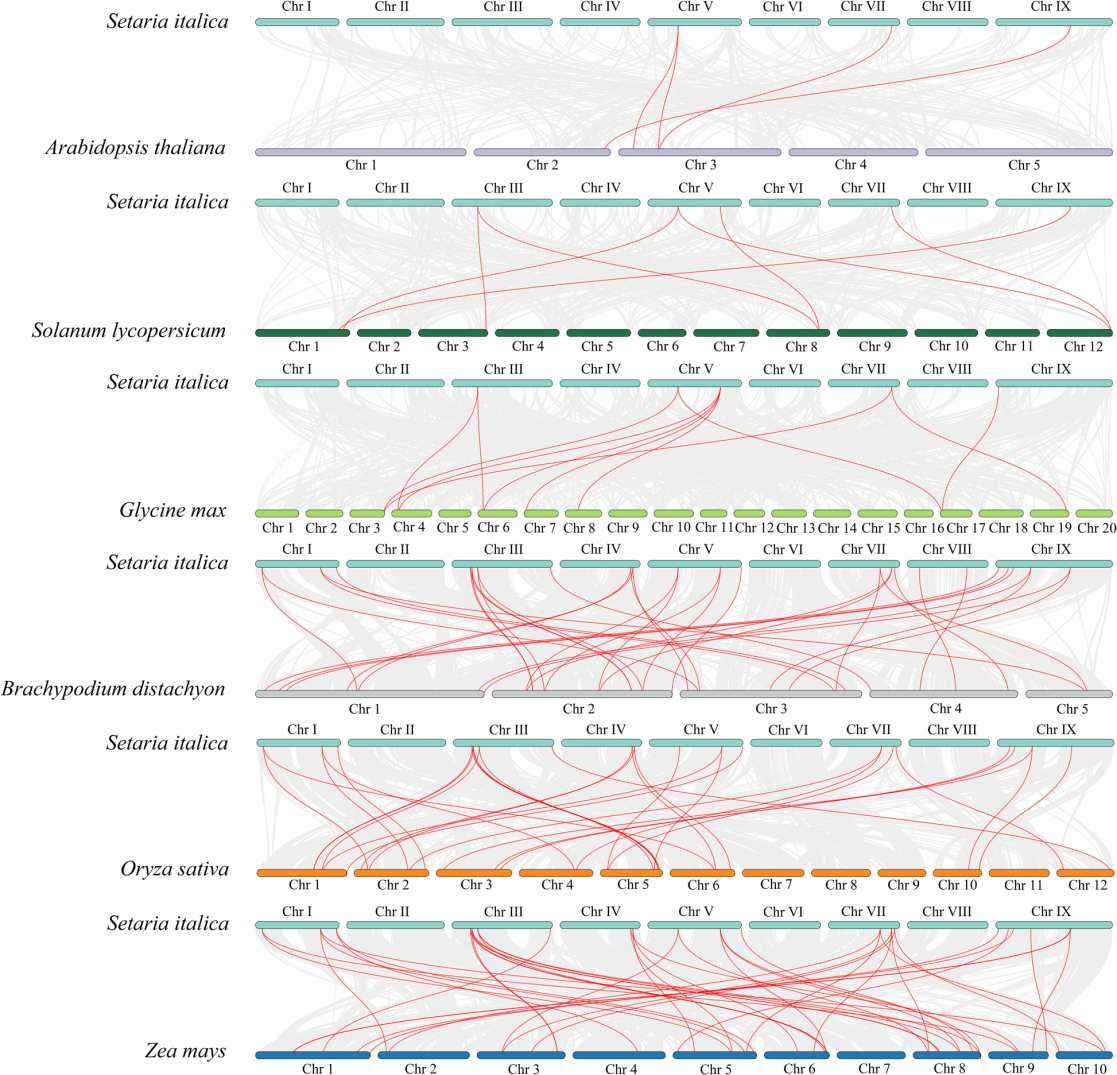
Synteny analysis of GATA genes between *Setaria italica* and six representative plant species (*Arabidopsis thaliana*, *Solanum Lycopersicum*, *Glycine max*, *Brachypodium distachyon*, *Oryza sativa*, and *Zea mays*). The gray line in the background shows colinear blocks in the genomes of *S. italica* and other plants, while the red line highlights colinear GATA gene pairs

To further study the evolutionary mechanism of the GATA family of foxtail millet, a collinear map of foxtail millet and six representative species was constructed, including three monocotyledons (rice, maize, and *B. distachyon*) and three dicotyledons (*A. thaliana*, tomato, and soybean). As shown in Fig. 5, the collinearity of the foxtail millet GATA genes with monocotyledons was better than that with dicotyledons. Among the monocotyledons, maize and millet had the best collinearity, with a total of 42 *SiGATA* collinearity genes found in these two species, followed by *B. distachyon* (35) and rice (31) (Table S6). In dicotyledons, there were fewer collinear *SiGATA* genes. In soybean, there were 11 collinear *SiGATA* genes, followed by tomato (7) and *A. thaliana* (4). Interestingly, *SiGATA11* and *SiGATA17* shared collinear genes with both monocotyledons and dicotyledons. *SiGATA27* was determined to have collinear genes with five other plants, except for soybean. These results suggest that *SiGATA11*, *SiGATA17*, and *SiGATA27* might be relatively ancient genes that existed before monocotyledon differentiation. In addition, we calculated the ka/ks values of the *SiGATA* gene pairs (Table S7) to better understand the evolutionary constraints acting on the *SiGATA* gene family. The gene in subfamily I had the highest ka/ks value of 0.71. The ka/ks value of subfamily IV was the lowest, at only 0.32. However, the ka/ks values of both subfamily pairs and duplicate event pairs were less than 1. This suggests that the *SiGATA* gene pairs have undergone uneven selection pressure during evolution.

### Evolutionary analysis of SiGATA proteins and the GATA proteins of several other species and protein interaction prediction

**Fig. 6:**
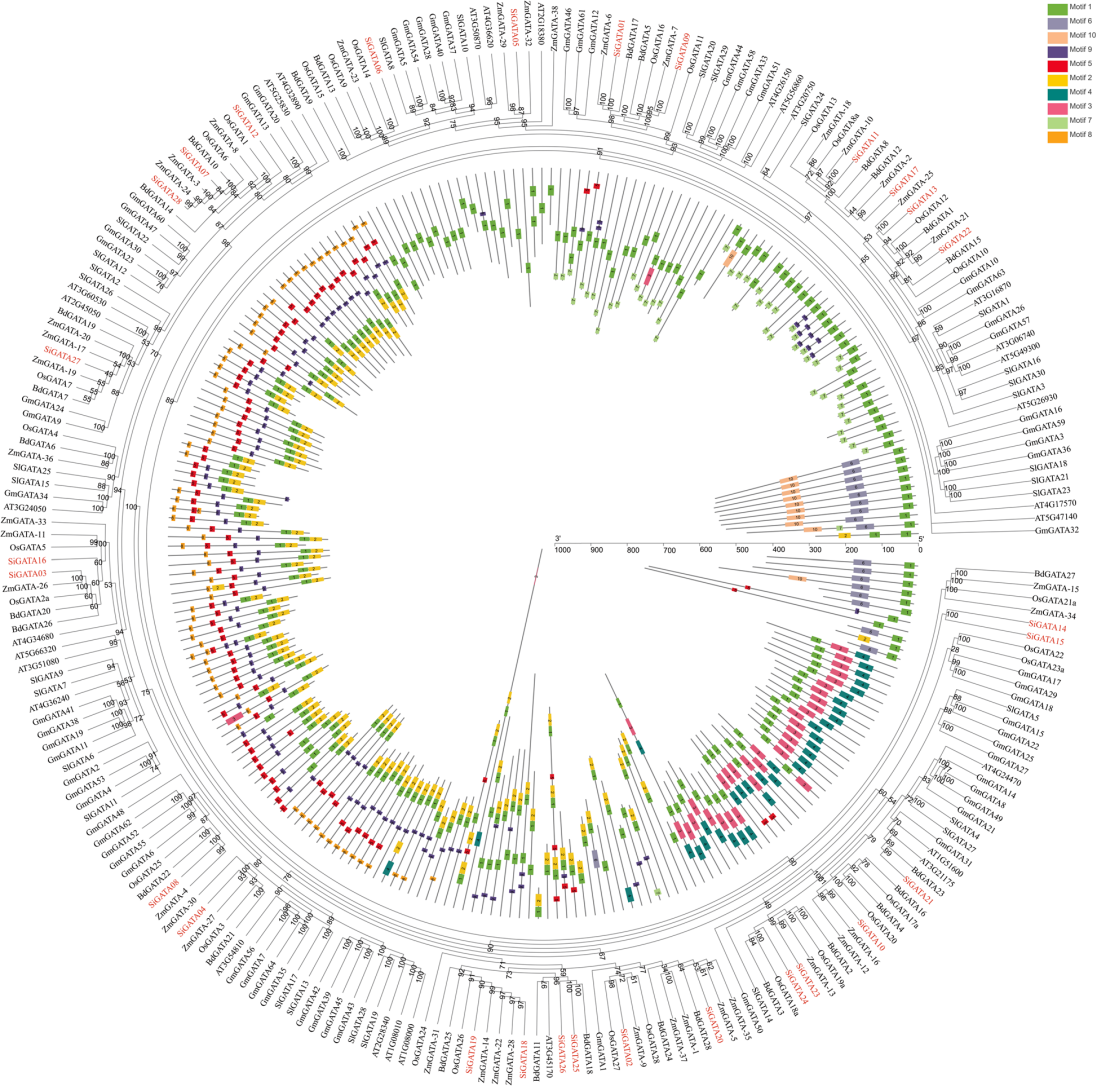
Phylogenetic relationships and motifs of GATA proteins from *Setaria italica* and six different plants (*Arabidopsis thaliana*, *Solanum Lycopersicum*, *Glycine max*, *Brachypodium distachyon*, *Oryza sativa*, and *Zea mays*). Outer panel: an unrooted phylogenetic tree constructed using the ML method. Inner panel: distribution of the conserved motifs in GATA proteins. Different colored boxes represent different motifs and their positions in each GATA protein sequence

To analyze the evolutionary relationship between the *SiGATA* family in foxtail millet and that of six species (*A. thaliana*, tomato, soybean, rice, *B. distachyon*, and maize), an unrooted ML evolutionary tree was constructed from the GATA amino acid sequences of these species (Table S2), and the conserved motifs of these proteins were analyzed (Table S3). As shown in Fig. 6, most *SiGATA* genes tended to cluster with these GATA genes of maize, *B. distachyon*, and rice, indicating that *SiGATA* genes were more closely related to GATA genes of monocotyledons. All GATA proteins from the six studied plants contained conserved motif 1 of GATA, but the conserved motifs and sequences differed greatly among different branches. The branch of *SiGATA* members in subfamily II contained more conserved motifs 1 and 7, and the branch of *SiGATA* members in subfamily I contained more conserved motifs 8–5–9–1 and 2. Subfamily III mainly contained conserved motifs 4–3–1, and subfamily IV mainly contained conserved motifs 1–6. These results indicate that motifs in the same subfamily have similar patterns, which might also indicate that these proteins have similar functions.

Therefore, we predicted interactions between 28 SiGATA proteins using STING. As shown in Figure S2, the results showed that among the 28 SiGATA proteins, 19 proteins were found to have possible interactions after prediction. Of the 19 interacting proteins, nine belonged to subfamily I and six to subfamily II. The fewest were in subfamilies III and IV, both of which have only two genes. Interestingly, SiGATA10, a member of subfamily III, was predicted to possibly interact with the 11 SiGATA proteins. Results suggested that both SiGATA14 (subfamily IV) and SiGATA15 (subfamily IV) could also interact with six SiGATA proteins. This implies that SiGATA10, SiGATA14, and SiGATA15 play important roles in the GATA family.

### Expression patterns of *SiGATA* genes in different tissues and fruit development of foxtail millet

**Fig. 7:**
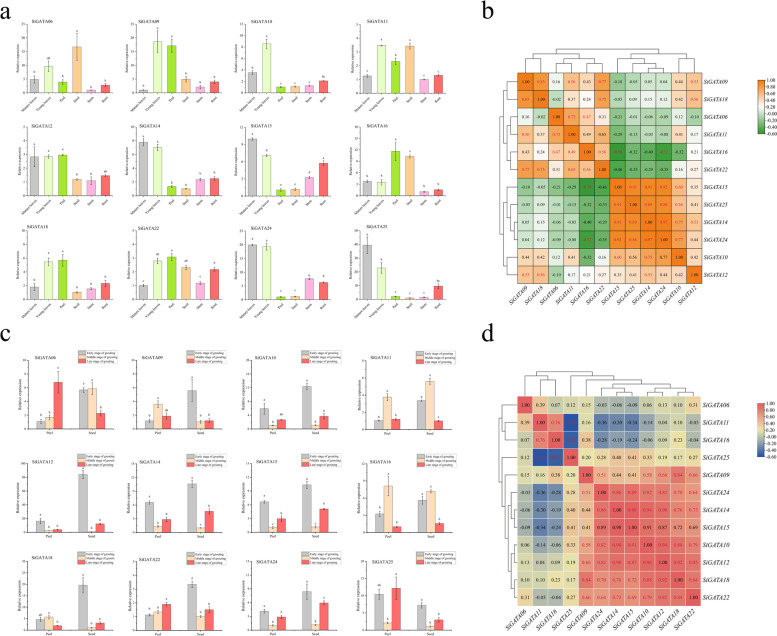
Tissue and Spatio-temporal expression patterns and correlation analysis of 12 *Setaria italica* GATA genes. **a** Tissue-specific expression pattern of GATA genes at the mid-grain filling stage. Expression profiles of 12 *S. italica* GATA genes in young leaves, mature leaves, roots, stems, peels, and seed organs. **b** Correlation analysis of tissue-specific expression of GATA genes. **c** Expression patterns of 12 *S. italica* GATA genes during fruit development. qRT-PCR was used to detect the expression of GATA genes in the peel and fruit before, during, and after grain filling. **d** Correlation analysis of GATA gene-specific expression during the grain-filling process. In the bar chart, the error bars were obtained from three measurements. Lowercase letters above the horizontal line indicate significant differences between treatments (α = 0.05, LSD). In the graph of the correlation analysis, a positive number indicates a positive correlation, whereas a negative number means a negative correlation. Red numbers indicate a significant correlation at the 0.05 level

To explore the expression of *SiGATA* genes in different tissues, we selected 12 *SiGATA* genes from different subfamilies and examined their expression patterns in six different tissues (mature leaves, young leaves, peels, seeds, roots, and stems). As shown in Fig. 7a, the expression of these genes was detected in the different tissues. Most *SiGATA* genes (11) were highly expressed in the leaves, and some *SiGATA* genes (six) were also highly expressed in seeds and peels. However, the expression of *SiGATA* in the roots and stems was low. Correlation analysis of the *SiGATA* expression levels in different tissues (Fig. 7b) showed that most genes were significantly positively correlated (P < 0.05). For example, *SiGATA10*, *SiGATA14*, *SiGATA15*, *SiGATA24*, and *SiGATA25* were positively correlated pairwise, and *SiGATA06*, *SiGATA11*, and *SiGATA16* were positively correlated. However, some genes were also negatively correlated. For example, *SiGATA16* was significantly negatively correlated with *SiGATA15* and *SiGATA24*.

The foxtail millet fruits are rich in various nutrients, and tissue-specific expression showed that many *SiGATAs* (six) were highly expressed in seeds and peels. Therefore, we further explored the expression pattern of *SiGATA* during fruit filling, the results of which are shown in Fig. 7c. *SiGATAs* were highly expressed in both the peel and seed at the early stage of grain filling and especially in the early stage of seed development. In addition, some genes, such as *SiGATA06*, *SiGATA11*, and *SiGATA16*, were highly expressed in the middle stage of grain filling. Correlation analysis revealed that *SiGATA10*, *SiGATA12*, *SiGATA14*, *SiGATA15*, *SiGATA18*, *SiGATA22*, and *SiGATA24* were positively correlated (p < 0.05) (Fig. 7d). There was a significant positive correlation between *SiGATA11* and *SiGATA16*, and a significant negative correlation between both and *SiGATA24*.

### Expression patterns of *SiGATA* genes in response to different abiotic stresses

**Fig. 8:**
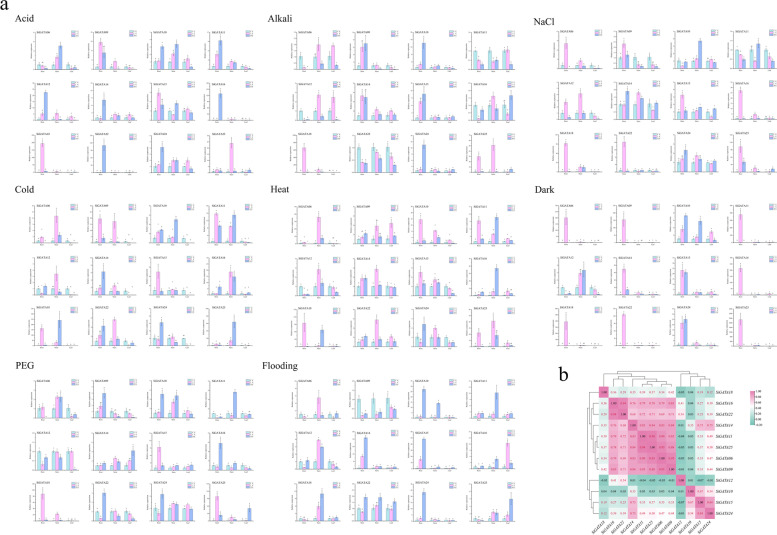
Expression of 12 *Setaria italica* GATA genes under abiotic stress (acid, alkali, NaCl, PEG, dark, flooding, heat, and cold) at the seedling stage. **a** qRT-PCR was used to detect the expression of 12 *S. italica* GATA genes in roots, stems, and leaves at different times. The error bars were obtained from three measurements. Lowercase letters above the horizontal line indicate significant differences between treatments (α = 0.05, LSD). **b** Positive number: positive correlation; negative number: negative correlation. Red numbers indicate a significant correlation at the 0.05 level

Eight abiotic stresses were applied to foxtail millet plants, and the expression patterns of 12 *SiGATA* genes were detected in the roots, stems, and leaves. As shown in Fig. 8a, *SiGATA* genes were induced or repressed to different degrees under different stresses. In particular, *SiGATA16*, *SiGATA18*, and *SiGATA25*, except for not being the most highly expressed under flooding, were highly expressed under the remaining seven stresses. Interestingly, the expression levels of *SiGATA10*, *SiGATA14*, *SiGATA15*, and *SiGATA24* were relatively high under flooding conditions, although they were also significantly induced under other stresses. The stress-induced expression of *SiGATA* in roots and stems was common in different tissues under stress treatment. However, in stress-treated leaves, although some genes were induced to be expressed, most of the *SiGATA* genes were inhibited. Regarding the response time of *SiGATA* gene expression after stress treatment, most genes were significantly expressed after 2 h of stress treatment. In addition, the expression data of the *SiGATA* genes after eight stress treatments were used for correlation analysis, the results of which indicated that most *SiGATA* genes were significantly positively correlated (p < 0.05) (Fig. 8b). For example, except for *SiGATA10* and *SiGATA12*, significant positive correlations were observed between the other genes. Interestingly, *SiGATA10* was only significantly positively correlated with *SiGATA14*, *SiGATA15*, and *SiGATA24*, and *SiGATA12* was only significantly positively correlated with *SiGATA16* and *SiGATA22*.

## Discussion

### Characteristics of *SiGATA* genes

Interestingly, 78.57% (22/28) of the SiGATA proteins had isoelectric points greater than 7, indicating that most SiGATA proteins were composed of more alkaline amino acids, which might be closely related to the GATA domain followed by a highly basic region [17]. The prediction results for subcellular localization showed nuclei (21), chloroplasts (5), plastids (1), and mitochondria (1). The prediction of subcellular localization was relatively accurate since most *SiGATA* genes were found to be located in the nucleus. Understanding the domain characteristics of the *SiGATA* genes are helpful to distinguish the differences between *SiGATA* genes. Most *SiGATA* genes only had one GATA domain, although there were exceptions, including two (*SiGATA8*, *SiGATA18*, and *SiGATA19*) or three (*SiGATA26*) GATA domains in some members of subfamily I. This result is similar to that of other species in the GATA family; that is, most plants have only one zinc finger domain for GATA factors [17, 23, 31, 41]. The presence of additional GATA domains in *SiGATA08*, *SiGATA18*, and *SiGATA19* could have key roles in different cellular processes. For example, *OsGATA26* (LOC_Os12g07120.1), the homologous gene of *SiGATA18* in rice, is significantly upregulated under salt, drought, and external ABA treatments; therefore, the additional GATA domain might play an important role in abiotic stress signaling [23]. In this study, we also found that *SiGATA18* was significantly upregulated under multiple stresses (acid, alkali, NaCl, cold, heat, darkness, and PEG), suggesting that multiple GATA domains could endow endure *SiGATA18* with the function of an active response under multiple stresses. Moreover, *SiGATA18* and *SiGATA19* are tandem duplication genes, which also implies that these genes might have some functional differences from the *SiGATA* genes containing only one GATA domain. Except for the special case of *SiGATA15*, which was found to lack the first CX_2_C structure, the *SiGATA* gene family consists of two types of GATA domains. Among them, the GATA domain contained by members of subfamilies I, II, and IV was CX_2_CX_18_CX_2_C, and that contained by members of subfamily III was CX_2_CX_20_CX_2_C. This result was similar to that of most GATA TFs in plants containing CX_2_CX_18_CX_2_C and CX_2_CX_20_CX_2_C zinc finger structures [16, 20]. The *SiGATA* gene family generally contains GATA domains of two zinc finger structures, but there are differences in some loci within the GATA domains of different subfamilies; therefore, the differences in these loci could impart different functions to different subfamily members. A schematic diagram of the full-length SiGATA protein shows that conserved motif 1 of the GATA domain was distributed in all gene members. However, different subfamilies had unique conserved motifs, which might further support the functional differences among SiGATA members in different subfamilies.

Plant GATA factors involved in the light signaling pathway often regulate light signal transduction by binding to the GATA motif in the promoters of light-related genes [42, 43]. At the same time, studies have also shown that GATA2 (*At2g45050*) is a key transcriptional regulator that integrates light and brassinosteroid signaling pathways [44]. This suggests that GATA can interact with light-related genes and that GATA itself might also be a light-related gene, which makes it more important to study cis-acting elements in the *SiGATA* promoter region. Many cis-acting elements were found in the promoter region of *SiGATA*. Light response-related elements, Box 4, G-box, and Sp1, exist in most gene promoter regions. In addition, 11 *SiGATA* gene promoter regions had GATA-motif cis-acting elements, suggesting that *SiGATA* genes might interact with each other and jointly participate in light signal transduction. In plants, GATA TFs are involved in the stress response, flowering, development, and hormone signal transduction, as well as other important biological processes [17, 23]. The results of cis-acting element analysis were also consistent with the function of GATA TFs, such as the environmental stress-related elements, hypoxia-inducible (GC-motif, ARE), low-temperature response (LTR), and drought-inducible (MBS) elements, which were found to be widely present in the *SiGATA* gene promoters. Subsequent experiments also showed that *SiGATA16* and *SiGATA25*, containing LTR cis-acting elements, were highly inducible under low-temperature stress. *SiGATA10*, *SiGATA14*, and *SiGATA15* contained ARE cis-acting elements, and their expression was found to be significantly induced under flooding stress. In addition, ABRE and methyl jasmonate cis-acting element (CGGTA-motif, TGACG-motif), root expression-related element (as-1), endosperm expression-related element (AAGAA-motif), and tissue expression-related element (CAT-box) were also present in most *SiGATA* gene promoters, suggesting that these *SiGATA* genes might be involved in stress responses, hormone signal transduction, and plant growth and development.

### Evolution of *SiGATA* genes

Zhang found that duplication events of foxtail millet genes are mostly generated by whole-genome duplication events that are shared by grasses [45]. Replication events of the *SiGATA* members were investigated within the whole genome of the foxtail millet, and it was found that there were three tandem duplication events and five segmental duplication events. It can be seen that these duplication events play a significant role in the amplification of the *SiGATA* gene, especially for subfamilies I and II with more *SiGATA* members. In addition, collinearity analysis of foxtail millet with three monocotyledons and three dicotyledons revealed that *SiGATA* genes had the most homology with maize (42), followed by *B. distachyon* (35), rice (31), and *A. thaliana* (4), which might be related to foxtail millet as a C4 crop among monocotyledonous plants [46]. Interestingly, among these homologous genes, *SiGATA11*, *SiGATA17*, and *SiGATA27* had colinear genes with almost all these six representative species. We speculate that *SiGATA11*, *SiGATA17*, and *SiGATA27* might be ancient and could have existed before the differentiation of monocots and dicots. The *SiGATA* and GATA genes in all six species showed that most *SiGATA* genes were clustered with GATA genes in monocotyledonous plants (maize, *B. distachyon*, and rice), suggesting that millet is more closely related to monocotyledonous plants. Furthermore, to understand the evolutionary constraints acting on *SiGATA* genes, we analyzed the ka/ks values of the *SiGATA* gene pairs. The results showed that the ka/ks value of the gene pair of subfamilies and the gene pair of duplication events were all less than 1, indicating that the *SiGATA* gene pairs have undergone purifying selection pressure during their evolution.

To further understand the function of the *SiGATA* genes, we used STRING [47] to predict the interaction between SiGATA proteins to better understand the unique position of certain genes in the evolutionary process. Among the 28 SiGATA proteins, 19 were determined to possibly interact with each other. SiGATA10 might interact with 11 SiGATA proteins including SiGATA1, SiGATA9, SiGATA12, and SiGATA28. The promoter regions of these four genes contained the GATA-motif, a cis-acting element, which further implies that SiGATA10 interacts with these four genes and participates in the light signal transduction pathway. SiGATA14 and SiGATA15 could interact with the six SiGATA proteins. However, among the proteins interacting with SiGATA14 and SiGATA15, the promoter region of SiGATA08 also contained GATA-motif cis-acting elements, similar to those in SiGATA10. These results suggest that SiGATA10, SiGATA14, and SiGATA15 might have a unique position and play an important role in the evolution of the GATA family. Although SiGATA10, SiGATA14, and SiGATA15 were not predicted to interact, they were found to interact with SiGATA01, SiGATA04, and SiGATA27. This suggests that SiGATA10, SiGATA14, and SiGATA15 could have synergistic effects on certain functions. Interestingly, this result was verified in subsequent experiments. The results of spatiotemporal expression and response expression under stress showed that *SiGATA10*, *SiGATA14*, and *SiGATA15* were significantly positively correlated. This indicates that these three genes could be functionally similar or jointly involved in some biological processes and further supports the accuracy of protein interaction prediction.

### Spatio-temporal expression patterns of the *SiGATA* genes and their response to abiotic stress

GATA TFs play important roles in many aspects of plant growth and development. For example, GATA-type TFs are involved in the regulation of signal transduction associated with plant hormones (GA and auxin). As direct targets of PIF TFs, *GNC* (GATA, NO3-inducible, and carbon metabolic-involved) and *GNL* (GNC-like) are involved in GA signal transduction. In addition, these two genes regulate the TF ARF2 to regulate auxin [24, 27]. *GNC* and *CGA1/GNL* play important roles in chlorophyll synthesis and might regulate nitrogen and carbon metabolism [48, 49]. *GNC* and *GNL*, members of GATA subfamily II, are most closely related to *SiGATA01* and *SiGATA09*. *SiGATA09* is highly expressed in the young leaves and peels of foxtail millet, particularly during the early grain filling stage of seed development. *CGa1* (cytokinin-responsive GATA1) can regulate chloroplast development in rice, and *OsCga1* overexpression maintains chloroplast development under low-nitrogen conditions, resulting in a reduction in plant size but an increase in the harvest index [50]. Interestingly, *CGa1* (*LOC_Os02g12790*) was found to be homologous to *SiGATA01* and *SiGATA09* in this study, which explains the high expression of *SiGATA09* in young leaves and peel of foxtail millet. SiGATA09 may be involved in chlorophyll synthesis. Zhang et al. found that HAN (HANABA TARANU, *At3g50870*) directly binds to the GNC promoter and acts as a suppressor of flower development, suggesting that GATA genes might also interact with their homologous genes [2]. This indicates that GATA TFs play an important role in plants. However, there might be interactions between GATA TFs, which is similar to the situation found in this study in that *SiGATA10* was suggested to interact with multiple *SiGATA* genes. GATA also plays an important role in the regulation of plant light signaling pathways, and *AT2G45050* (*GATA2*) has been identified as a key transcriptional regulator of the integration of brassinosteroid (BR) and light signaling pathways [44]. In *A. thaliana*, *ATGATA2* is highly expressed in hypocotyls and petioles, which is a key light signal regulator that mediates the interaction between brassinosteroid and the light signal pathway [44]. *SiGATA27* is a gene homologous to *GATA2*, but the expression pattern of *SiGATA27* was not detected in this study. Therefore, the functional analysis of *SiGATA27* is worthy of further study. However, for *SiGATA07*, *SiGATA12* and, *SiGATA28*, which belong to the same subfamily and have a close evolutionary relationship with *SiGATA27*, the promoter regions of *SiGATA12* and *SiGATA28* all contained GATA-motif cis-acting elements. This further suggests that *SiGATA12* and *SiGATA28* are involved in the optical signal transduction pathway.

In addition to its important role in plant growth and development, GATA plays an important role in the response to abiotic stress. For example, under salt stress, the biomass accumulation of *OsGATA8*-overexpressing lines were higher than that of wild-type rice. In addition, different environmental stresses (such as drought, salinity, and ABA) increase the expression of *OsGATA8*, which integrates leaf greening, biomass production, reactive oxygen species clearance, and ion homeostasis, and improves tolerance to stress [30]. In addition, in terms of plant cold tolerance, OsGATA16 binds to the *OsWRKY45-1* promoter and inhibits its expression, thus improving cold tolerance in rice at the seedling stage [51]. In this study, *SiGATA01* and *SiGATA09* were found to be homologous genes of *OsGATA16*, and the expression levels of *SiGATA09* in roots and stems under 2 h of cold stress were significantly increased by approximately 15-fold compared to the control. Therefore, *SiGATA09* might improve the cold tolerance of plants. Members of GATA subfamily III have been reported to contain a tify domain, which plays an important regulatory role in the plant stress response [52]. This study also found that *SiGATA10*, *SiGATA23*, and *SiGATA24*, which are members of the *SiGATA* subfamily III, also have a tify domain. However, the expression levels of *SiGATA10* and *SiGATA24* were significantly increased under different stress treatments, especially in 24-h roots under flooding stress. In this study, many genes were found to respond positively to stress, especially *SiGATA16*, *SiGATA18*, and *SiGATA25*. These three genes were highly expressed in the roots and stems under multiple stress treatments; therefore, these three genes might lead to relative stress tolerance. Interestingly, tissue-specific expression analysis showed that *SiGATA* genes were highly expressed mainly in the leaves of normal growing plants, whereas most *SiGATA* genes were highly expressed mainly in the roots and stems after stress treatment. Although the reason for this difference was not clear, the mechanism underlying this difference is worthy of further study. In addition, *SiGATA10* is worthy of attention because its expression is induced under various stresses, with the interaction prediction results suggesting that it interacts with other *SiGATA* genes. Therefore, whether it has a unique function among the *SiGATA* genes warrants further investigation.

## Conclusion

In this study, the subfamily classification, gene structure, chromosome location, repeated events, and expression patterns of the foxtail millet GATA TF family were comprehensively analyzed for the first time. In total, 28 *SiGATA* genes were found in foxtail millet, providing a solid foundation for further verification of the functions of the *SiGATA* genes. Additionally, we analyzed the expression of 12 *SiGATA* genes from different subfamilies in the different tissues of millet fruits and at different stages of grain filling and found that *SiGATA* genes might be involved in foxtail millet development. Furthermore, the expression patterns of these genes under eight different abiotic stresses were explored, and *SiGATA* genes that responded positively to multiple stresses were screened. The results presented in this study provide insights into the function of *SiGATA* genes and provide a reference for the molecular breeding of foxtail millet in future studies.

## Materials and methods

### Foxtail millet plant materials, growth conditions, and abiotic stress treatments

The foxtail millet used in this study was Yugu 1, a northern cultivar. The test materials were all planted in a greenhouse, and samples of the peel and fruit in the early, middle, and late stages of grain filling, as well as the root, stem, and leaf samples in the middle stage of grain filling were obtained. The principle of sample selection was based on plants with the same growth conditions and five replicates. Foxtail millet plants at the seedling stage (28 days) were treated with eight abiotic stresses, including salt (5% NaCl), acid (HCl 0.1 mol/L), alkali (NaOH, 0.2 M), darkness (complete shading), flooding (whole plant), heat (40 °C), drought (30% PEG6000), and cold (4 °C). According to Fan et al.’s method, acid, alkali, salt, and drought stress were applied [12, 53]. That is, under the same stress conditions, different repetitions with the same volume of liquid immersion roots were used. Five replicates of each stress treatment were used, and the roots, stems, and leaves of millet plants were sampled after 0, 2, and 24 h of stress treatment. All samples were immediately frozen in liquid nitrogen and stored at − 80℃. Twelve *SiGATA* genes selected from different subfamilies were analyzed using qRT-PCR to detect the expression pattern of *SiGATA* genes.

### Total RNA extraction, cDNA reverse transcription, and qRT-PCR analysis

Total RNA was extracted from all samples using an RNA extraction kit (TaKaRa Bio) and reverse-transcribed into cDNA for qRT-PCR analysis. The qRT-PCR primers (Table S6) for the 12 *SiGATA* genes were designed using Primer 5.0. Si001873 mg (*ACTIN* gene) was used as an internal control. Standard RT-qPCR with SYBR Premix Ex Taq II (TaKaRa Bio) was repeated at least three times on a CFX96 real-time system (Bio-Rad). The experimental data obtained by qRT-PCR were analyzed using the 2^− (ΔΔCt)^ method.

### Genome-wide identification of *SiGATAs* in foxtail millet

Two BLAST methods were used to identify these GATA genes of millet. First, the whole millet genome was aligned with the GATA genes of *A. thaliana* and rice to obtain candidate GATA genes. Next, the hidden Markov model of the GATA domain (PF00320) was used to search for GATA proteins using HMMER 3.0 software (default parameters; http://HMMER.org/). Finally, all candidate genes were verified using Batch CD-Search (https://www.ncbi.nlm.nih.gov/Structure/bwrpsb/bwrpsb.cgi) and the SMART tool (http://SMART.embl heidelberg.de/). Protein length, molecular weight (MW), PI analysis (https://web.expasy.org/compute_pi/), and protein subcellular localization prediction (https://wolfpsort.hgc.jp/) were performed for all SiGATA proteins.

### Phylogenetic analysis, classification, chromosomal distribution, and gene duplication of the *SiGATA* gene family

Phylogenetic trees were constructed using protein sequences of other species (*A. thaliana*, *Oryza sativa*, *Solanum Lycopersicum*, *Glycine Max*, *Zea Mays*, *B. distachyon*) with muscle wrapper. Then, an ML phylogenetic tree was built with an IQ-tree wrapper (bootstrap number set to 1000) and the best substitution model was automatically selected. The classification method for the GATA gene family in *A. thaliana* could be used as a reference for the classification of the GATA gene family in millet. The physical location information of the *SiGATA* gene was obtained from the whole millet genome, and *SiGATA* was located on the chromosome according to this information. The Collinear Scanning Toolbox (MCScanX), with default parameters, was used to scan *SiGATA* genes for collinearity to obtain a record of their gene duplication events. The homology between *SiGATA* and GATA genes among different species was analyzed using a multiple synteny plot (https://github.com/CJ-Chen/TBtools) [54].

### Gene structure, conserved motifs, cis-acting element analysis, and protein interaction prediction of *SiGATAs*

A gene structure map was obtained by comparing the CDS sequence of *SiGATA* with the corresponding genomic DNA sequence. The full-length conserved motif of the GATA protein was obtained using the online MEME tool (http://meme-suite.org/tools/meme), and the maximum conserved motif search value was set to 10. The promoter sequence 2 kb upstream of the* SiGATA* gene was used with PlantCARE (http://bioinformatics.psb.ugent.be/webtools/plantcare/html/) to analyze the cis-acting elements in the promoter region. Protein interactions were predicted using STRING (https://string-preview.org/). In addition, the ka/ks value was calculated using the ka/ks calculator.

### Statistical analysis

Analysis of variance (*p* < 0.05) was performed using JMP6.0 software (SAS Institute) and compared with the least significant difference (LSD) test at the 0.05 and 0.01 levels. Pearson analysis was used for correlation analysis. All histograms were drawn using OriginPro2019b software (OriginLab).

## Supplementary Information

**Additional file 1: Figure S1**. Cis-acting element Venn diagram of *SiGATA* genes ATG upstream 2000-bp promoter sequence. (**a**) Environment related element, (**b**) growth related element, (**c**) hormone related element, (**d**) promoter related element, and (**e**) light response related element. **Figure S2**. Prediction results of protein‒protein interaction networks among 28 SiGATA proteins. **Table S1**. List of the 28 *S. italica* GATA genes identified in this study. **Table S2**. GATA protein sequence information from six representative species for phylogenetic tree analysis. **Table S3**. Analysis and distribution of the conserved motifs in GATA proteins of *S. italica* and other species. **Table S4**. *SiGATA* gene promoter region cis-acting element details. **Table S5**. Tandem duplication and fragment duplication events of the *S. italica* GATA gene. **Table S6**. One-to-one orthologous GATA gene relationships between *S. italica* and other plants. **Table S7**. Ka/ks values of each subfamily gene pair and all duplication events gene pairs. **Table S8**. Primer sequences for qRT-PCR.

## Data Availability

The entire *Setaria italica* genome sequence information was obtained from the Ensembl Genomes website (http://ensemblgenomes.org/). *S. italica* materials (Yugu 1) used in the experiment were supplied by Prof. Cheng Jianping of Guizhou University, and this material was used with permission from Guizhou University and Prof. Jianping Cheng. The datasets supporting the conclusions of this study are included in the article and its additional files.
