# Supplementary material for: Genome‑wide identification, phylogenetic and expression pattern analysis of GATA family genes in foxtail millet (Setaria italica)
Source: BMC Genomics. 2022 Aug 2;23:549. doi: 10.1186/s12864-022-08786-0 (PMC9347092; doi:10.1186/s12864-022-08786-0)
Supplement: Supplementary file 1 — Additional file 1: Figure S1. Cis-acting element Venn diagram of SiGATA genes ATG upstream 2000-bp promoter sequence. (a) Environment related element, (b) growth related element, (c) hormone related element, (d) promoter related element, and (e) light response related element. Figure S2. Prediction results of protein‒protein interaction networks among 28 SiGATA proteins. Table S1. List of the 28 S. italica GATA genes identified in this study. Table S2. GATA protein sequence information from six representative species for phylogenetic tree analysis. Table S3. Analysis and distribution of the conserved motifs in GATA proteins of S. italica and other species. Table S4. SiGATA gene promoter region cis-acting element details. Table S5. Tandem duplication and fragment duplication events of the S. italica GATA gene. Table S6. One-to-one orthologous GATA gene relationships between S. italica and other plants. Table S7. Ka/ks values of each subfamily gene pair and all duplication events gene pairs. Table S8. Primer sequences for qRT-PCR. [file 12864_2022_8786_MOESM1_ESM.pdf]

Supplementary material

Figure S1. Cis-acting element Venn diagram of *SiGATA* genes ATG upstream 2000-bp promoter sequence. **(a)** Environment related element, **(b)** growth related element, **(c)** hormone related element, **(d)** promoter related element, and **(e)** light response related element.

Figure S2. Prediction results of protein–protein interaction networks among 28 SiGATA proteins.

Table S1. List of the 28 *S. italica* GATA genes identified in this study.

Table S2. GATA protein sequence information from six representative species for phylogenetic tree analysis.

Table S3. Analysis and distribution of the conserved motifs in GATA proteins of *S. italica* and other species.

Table S4. *SiGATA* gene promoter region cis-acting element details.

Table S5. Tandem duplication and fragment duplication events of the *S. italica* GATA gene.

Table S6. One-to-one orthologous GATA gene relationships between *S. italica* and other plants.

Table S7. Ka/ks values of each subfamily gene pair and all duplication events gene pairs.

Table S8. Primer sequences for qRT-PCR.

Figure S1. Cis-acting element Venn diagram of *SiGATA* genes ATG upstream 2000-bp promoter sequence. **(a)** Environment related element, **(b)** growth related element, **(c)** hormone related element, **(d)** promoter related element, and **(e)** light response related element.

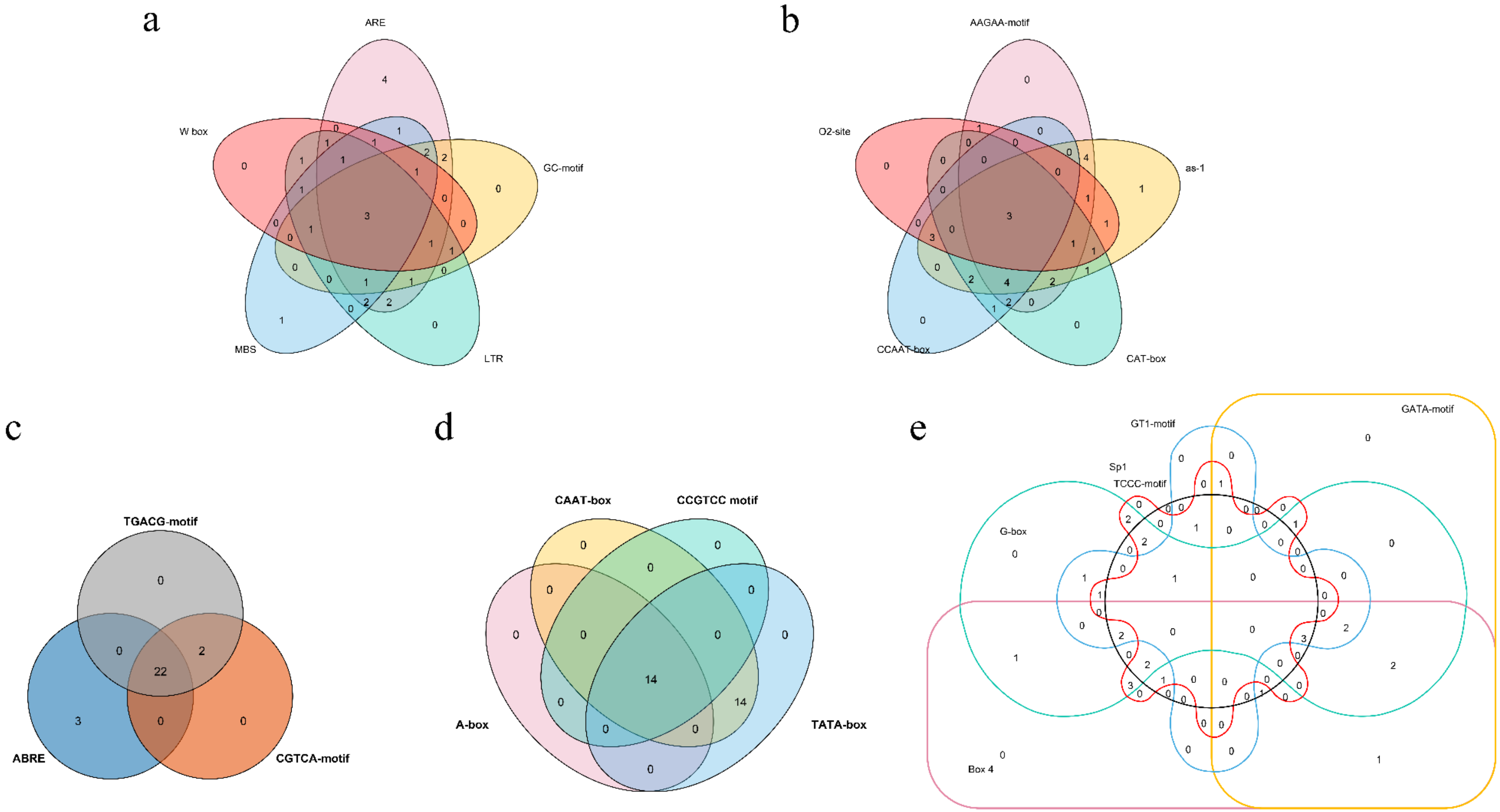

Figure S2. Prediction results of protein–protein interaction networks among 28 SiGATA proteins.

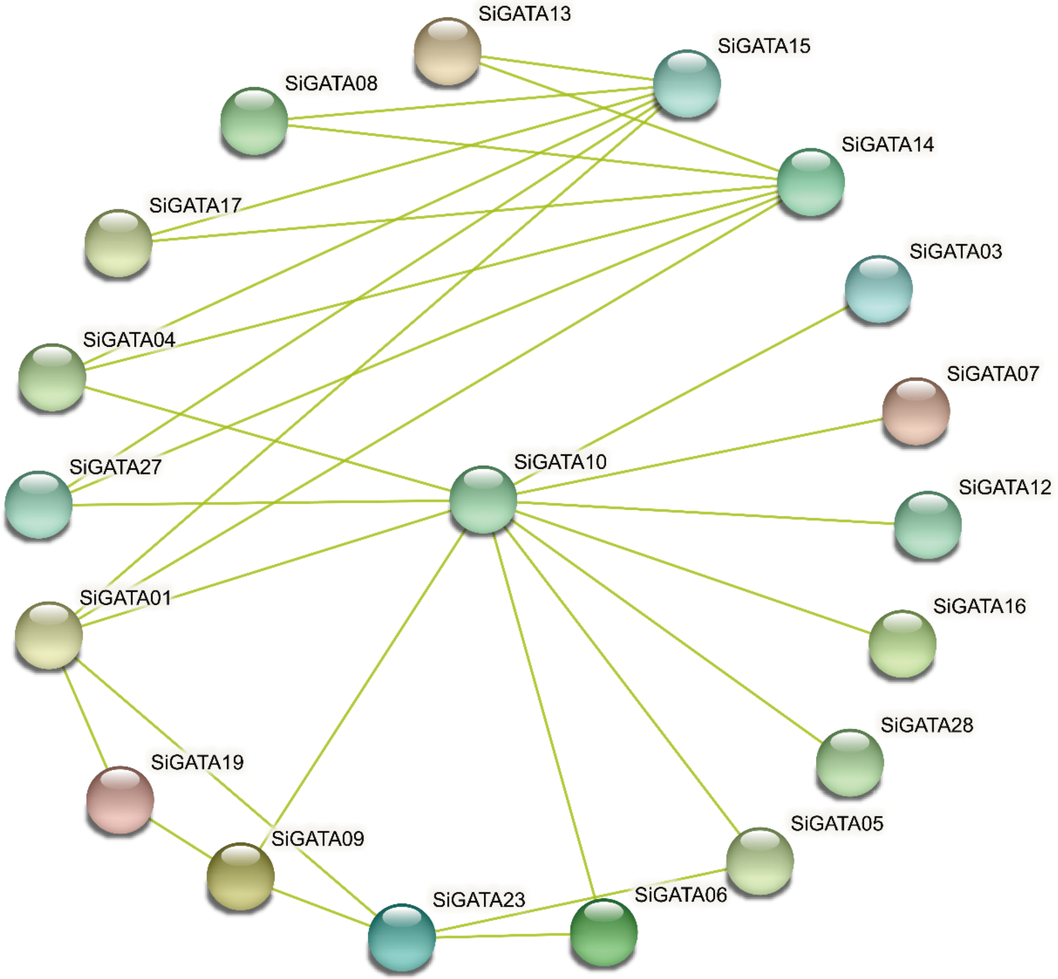

Table S1. List of the 28 *S. italica* GATA genes identified in this study.

Supplementary Table S1-1 List of the 28 *SiGATA* genes identified in this study.

Group	Gene	Gene ID	Transcript	Protein length	Intron number	Mw(KDa)	pI	Chr Location	Domain	Predicted Subcellular location(W)
Subfamily I	SiGATA16	SETIT_010126mg	transcript:KQK98295	442	2	46.32	7.26	VII	ZnF_GATA	chlo
Subfamily I	SiGATA03	SETIT_017219mg	transcript:KQL30710	442	2	45.84	8.80	I	ZnF_GATA	chlo
Subfamily I	SiGATA04	SETIT_017317mg	transcript:KQL32016	423	1	44.41	5.37	I	ZnF_GATA	nucl
Subfamily I	SiGATA07	SETIT_022327mg	transcript:KQL14695	384	1	39.37	5.63	III	ZnF_GATA	nucl
Subfamily I	SiGATA12	SETIT_001735mg	transcript:KQL06949	400	1	40.96	5.68	V	ZnF_GATA	nucl
Subfamily I	SiGATA02	SETIT_019572mg	transcript:KQL28931	317	2	33.99	7.09	I	ZnF_GATA	nucl
Subfamily I	SiGATA27	SETIT_036273mg	transcript:KQK89954	368	1	37.85	7.32	IX	ZnF_GATA	nucl
Subfamily I	SiGATA20	SETIT_028169mg	transcript:KQK93911	417	1	43.89	8.65	VIII	ZnF_GATA	nucl
Subfamily I	SiGATA08	SETIT_022727mg	transcript:KQL17328	313	1	35.24	8.78	III	ZnF_GATA	nucl
Subfamily I	SiGATA25	SETIT_039011mg	transcript:KQK88841	292	1	31.18	9.00	IX	ZnF_GATA	nucl
Subfamily I	SiGATA28	SETIT_040064mg	transcript:KQK89964	325	3	34.63	9.33	IX	ZnF_GATA	nucl
Subfamily I	SiGATA19	SETIT_010130mg	transcript:KQK99540	440	0	47.38	9.73	VII	ZnF_GATA	nucl
Subfamily I	SiGATA26	SETIT_034871mg	transcript:KQK88842	580	1	61.68	10.12	IX	ZnF_GATA	nucl
Subfamily I	SiGATA18	SETIT_010259mg	transcript:KQK99539	403	0	42.78	9.56	VII	ZnF_GATA	plas
Subfamily II	SiGATA06	SETIT_024214mg	transcript:KQL14269	231	1	23.56	8.76	III	ZnF_GATA	chlo
Subfamily II	SiGATA09	SETIT_006733mg	transcript:KQL11659	363	2	38.55	8.99	IV	ZnF_GATA	nucl
Subfamily II	SiGATA05	SETIT_022366mg	transcript:KQL14176	379	2	39.47	9.28	III	ZnF_GATA	nucl
Subfamily II	SiGATA13	SETIT_002983mg	transcript:KQL08815	199	1	21.96	9.40	V	ZnF_GATA	nucl
Subfamily II	SiGATA01	SETIT_019058mg	transcript:KQL28093	325	3	34.70	9.41	I	ZnF_GATA	nucl
Subfamily II	SiGATA11	SETIT_004417mg	transcript:KQL05071	207	2	22.54	9.58	V	ZnF_GATA	nucl
Subfamily II	SiGATA17	SETIT_011269mg	transcript:KQK99264	141	2	15.36	9.82	VII	ZnF_GATA	nucl

Subfamily II	SiGATA22	SETIT_037892mg	transcript:KQK86338	152	2	16.56	9.88	IX	ZnF_GATA	nucl
Subfamily III	SiGATA23	SETIT_036747mg	transcript:KQK87242	309	7	33.00	8.66	IX	GATA/CCT/tify	chlo
Subfamily III	SiGATA21	SETIT_027518mg	transcript:KQK95022	160	4	17.64	10.18	VIII	GATA/CCT	chlo
Subfamily III	SiGATA10	SETIT_006750mg	transcript:KQL11783	358	6	37.90	5.01	IV	ZnF_GATA/CCT/tify	mito
Subfamily III	SiGATA24	SETIT_036492mg	transcript:KQK87704	340	8	36.00	4.77	IX	GATA/CCT/tify	nucl
Subfamily IV	SiGATA15	SETIT_011929mg	transcript:KQK97873	247	1	26.48	6.66	VII	GATA	nucl
Subfamily IV	SiGATA14	SETIT_010863mg	transcript:KQK97872	263	2	28.37	7.11	VII	GATA	nucl

Supplementary Table S1-2 Twenty-eight SiGATA protein sequences in this study

Protein	Sequences
SiGATA01	MDAPGEQDQGLFPAFHISKDPPILFPFMINNPVDQLQGQSSYGDQHLRQQVLAESTQQFTDRMMMSGSDIFPRPPLFRPTIQSIDGDMIQRSAYDPYDIESKRADGWAVAPPAAKMKIMR<br>KATSEYPEGGTARKPRRRAQAHQDESQQQLQQQQAMGVVRVCSDCNTTKTPLWRSGPCGPKSLCNACGIRQRKARRAMAAAAAAAAAAASNGGTPQAASVAVQAKAPKKEKRAD<br>VDRSLPFKKRCKMVAVDHAVTAAKATPAVAASTKDQDHVSSDKVAAAATSLQSKAASPPPAAALHVFPAADEVTDAAMLLMTLSCGLVRS*
SiGATA02	MREHRFLYDFPDVADCEGSCVGGGGPPCLLHAGLCCPDDPLETIMDNPDVFSDDLLEDLFIAAALPPPHRGWLPITIGGGGERVVDVDGADVGYRDRGGASSDDGAPRLPAAHAAMSA<br>DDSSWTTSSVATKSPPSLETESSPPSVSRLVVPRKKRDSSVKRGKRLWSLDIPNVPASSDNSSGRDDGDQDGRRNIIHGGGGGVRQRLLIARPPRNSRTQRACSHCASTETPQWRAGPDG<br>PGTLCNACGIRYKMNKLLPEYRPSTSPSFRSDKHSNRHRKVVKLREKVKVEKIVNMPLAPADCGGDFMDTHGCLIVIL*
SiGATA03	MTHQTLISSAPAFSPASHSLHASTPLLFTRSSPSSSSCSPSAAAAAPAMASFVAHHHGSLVEREGRMAALRSSLRPCEAAEEVDVAPAACGAADRGAGLFGDGFSVEDLLDLEDLCEVDK<br>DCAELGEATPAPAAVEEDKLSSDSHGSSVVSYELMALPPPVIDLSLPAHDAEELEWVSRIMDDSLAELPPPPRLPAAPLAAAARRPPLERAVPAPAAPAPMRSPTICALSTEALVPVKARR<br>SKRSRASVWSLSGGAPPSDSTSSSSTTTTSSCSSSASFSSFLFLPAESPPPFWAAHLLGEAPPRGSKKSKHGKHGKNNGKPKKRGRKPKHHPAASHFAGGAAASPPVPGDRRCSHCGVQK<br>TPQWRAGPEGAKTLCNACGVRYKSGRLLPEYRPACSPTFVSSIHSNSHRKVLEMRRKKEGVVLIPGPAPLPAAAPAVASF*
SiGATA04	MASGGFVEEMMREQSLLEATCGDLFDHIDDLLDFPKEESAADVLLLDAPAPGSPLSARIIDVGARGNNAQSAPAPPAMEPLMALPPPAAQDDATAFFAAAGAPVFDAKDVGGHIGSCEE<br>LDMDMAQLEWLLFDDASIPHEPAFPGINCPAPIKSSALAANNAGVALLPPEKMEDAIFRSSSPISVLEHNSFNANNNVGSASSSSSSASSSSESFSGSGGHAWSAPVSPRPEPPVLVIPARAR<br>SKRSRPSAFTGAARAAEAPTILVPTPMYSSTSSHSDPESIAESNPHQAPPMKKKKKAKKPAPPAPAASDAEGDNDGDADYEEGGERSQPQGGAVRRCTHCQIEKTPQWRAGPLGPKTLC<br>NACGVRYKSGRLFPEYRPAASPTFVPSIHSNSHKKVVEMRQKAVRTGDPSCDLLQFIRRRD*
SiGATA05	MRDMRGRPGPRVGDCSAPRGHMTRPPPAFDRPIARDAVDTSRVDLAISATAREALSKLLHCRPSPSSIATSSVPHLLLGSSARRLVFNSRTVASLRTMLRHCTGNSHHQSVATAAPGKT<br>MASTTFSLFFPLPTKGPWPPAAADEAGAFDDDRSSITTSPSSPSSSSSAGSVDCTLSLGTPSSRRAAAEPAEQAKRAAAQPAYPSVSASASASAVSWDVAADQPYYYCCHGSKPAAAGAA

KGAVARAEHDQLLVDRRCANCGTSSTPLWRNGPRGPKSLCNACGIRFKKEERRAAATAMDQGQGACGYVAQRAQYGTKQAPADAVPYYGEAAFPCGGGDVADAEAAPFLAWRLN
VVAPSPAPAPAFAVWPERTSLFQYN*

SiGATA06
MLQELALCACGRFYAGGCGGRCAGAAAASSAFSMLFPVAVEQCYYSKEDDRSPYVGGGAVDCTLSLGTPSTRRAEAGGRARAAGGGGGGAAPSCKESADRGSPPARRCANCDTTST
PLWRNGPRGPKSLCNACGIRYKKEERRAAAAVAPAPAPQDGSYACGGYARQPPPPQWGCYGPVAAAKSASFGMYGGGDVVDAAADGPCLSWMLNVMPSSPAFAVRERPTLFQYY*

SiGATA07
MEVAAECAGGARVKKEADLFLVDDLLDLPCDEEEEVQEAVVEEGEGEGGKAGVCGSGAGGEEGAAGNASNDSSTVTVLDSCSNSLSGLADGDFSGGLVEPYDQLAELEWLSNYMGE
DNFPTEDLKKLQLITGIPPASSSAAATASVPAPAAAAAPPAGGVLPPEAPVPGKARSKRSRIAPCSWASRLLVLPPPPASPPSPASAAISPSESGTAAPAFPAKKPSKPAKKKEPLTTPAPNA
AAAAAAAASAGEGRRCLHCETDKTPQWRTGPLGPKTLCNACGVRYKSGRLVPEYRPAASPTFVVSKHSNSHRKVLELRRQKEAHLHPHHQYQPQPQAMGHVGAGAAGGLMHAPSPL
LFDGPTGPLIGDDFLIHNRIGPDFRQLI*

SiGATA08
MDVHSANAAADSLGDLFPHQAAALESDESNLEWLSGYVEDCFSSSTSYTNPVFARPAPTMANQGAGKPKLPPPPPSNGRRKRRSLASVMGNDDDQQYIIPLYVEPPLLLIDQKHWMAE
SELILPKKDKDQEVCQQQEQEQEEEKCEKGALVPRQERLVKRCSNCLSCETPRWRNGPSGIQMLCNACGLRLKPENRFATISEEHYSQETKKEQEPGKRLDKKKKMIKKTYVSKELSSE
QAEKRCTHCMSSKTPQWRSGPLGPKTLCNACGVRYKSGRLLPEYRPANSPTFVSCLHSNSHKKVMQMRQAIAYKE*

SiGATA09
MSAIYMSQLSTALPLMEGDHHQDHHHHQGHFQAFNLPKDPPILFPFVISNSSASDSSLSYGSGDHHHLFRQQNQAMLEPQHMIGVSSAASVFATPFPTVESIRDDMIEPSSYDPYDMGRL
QVGGSLETAGSWTTPPAKMRITRKASTDPGAAKKPRRKVQGYEDMMGTSGQPNLGVNRVCSDCNTTKTPLWRSGPCGPKSLCNACGIRQRKARRAMMASAGGSGPAPADGAKAAT
ATPRDAMAASVHHPKVKKEKRIDVDRSLPFKKRCKVVQDHAPVAAPPAAAHKAAAVPPTGEVVDASGLSSRDLVDDIGLISWSRSPAPPSSAASCSFRSSPGLPVQQDEVTDAAMLLM
TLSCGLVRS*

SiGATA10
MSHHDGSKPYQPRRGPERHPQPADEVAAPPPAAVAPTVDHLAAVAAEAEALNRYTEEQQQQQMLQGHEQAGEDEEEEDGEEEEMEEDEDEQEGGQDGGVGAEHVPMDADAAAAA
AAAAAAGAQMDPHGSMVPGAVPPMANNQLTLSFQGEVYVFDSVSPDKVQAVLLLLGGRELNHPGLGAGSSSGAYNKRLNFPHRVASLMRFREKRKERNFDKKIRYSVRKEVALRMQ
RNRGQFTSSKPKPDEIAASEMVTADGSPNWGSVEGRPPSAAECHHCGTSATATPMMRRGPDGPRTLCNACGLMWANKGLLRDLSKSPVPLQAVQSAPVLDGGNGSVIAAPGSELENP
AAAMANGHES*

SiGATA11
PPPPPSPTHRIHSFLYPPFPSPQLLSALYYTLPRNRATHADQTESQNSPSDPVANPATDRGCRLFPPPPLPLPMGSADRSEIDGVVVAEKGARSCVECRATTTPMWRSGPTGPRSLCNACGI
RYRKKRRQELGLDHKQQQQQNQQQRQQHNGEAKTEVKDSSSNSSSSGSSNLQAVQKRRLLMGVEEAALLLMTLSSSPTSTLLHG*

SiGATA12
MEAAAAAEYGYYGGGGAGPRERKGAAGCGDHFVVDDLLVLPYDDDEEGDGEAAAAGDGGAPPCLQAAGAGGGVKEEGGLGNFSADSSTVTALDSCSNSFSGLADGDFPGEFCEPY
DQLAELEWLSNYMGEGDDAFATEDLQKLKLISGGYSPAVNVPPAPLAPAAAASAVSAAQPGMFIPEAPVPAKARSKRSRAAPGNWSSRLLVLPPPPASPPSPASMAISPAESGVSAQAFP
AKKPSKPSKKKDAPPAPQALPSSASAVQSAGSAASAEGRRCLHCETDKTPQWRTGPMGPKTLCNACGVRYKSGRLVPEYRPAASPTFVMSKHSNSHRKVLELRRQKEVQHQPPHQAH
VIAGGGPGGLMHMQSSLLFDGPAAAPIVAGDDFLIHHHLGADYRQLI*

SiGATA13	MEVEVEVETDPNTSPDEATASGEPKACADCHTTKTPLWRGGPEGPKSLCNACGIRYRKRRRQALSLDAAQQQDQQPSKKAAADPQQDDQQPQPKKKPAADSQEDQQPQPKKKPAAD<br>SQEDQQLQPKKPAAAASSTTTNKKDKKKKKDRQVTVELRVVGFGKEVMLKQRRQMRRKKCMSEEERAAVLLMAMSSGVIYAS*
SiGATA14	MGKQGPCRHCGVTSTPLWRNGPPDKPVLCNACGSRWRTKGSLANYTPMHRKDDIDDDEPRVSKLKPPTSKMKSQKKKTNHITTENGPFSGQSFQKMGDADPSSRSSSGSAISYSESCV<br>PYGAVDASEMSGSAQSHAWESLVPSRKRSCATRLKPSPVEKLVKDLNSIMHEEQLYYLSGSSEEDLLYHSDTPVGSFETGSGSVLLRHPNSKSPEEESEASSIPADDKSHITSESYSGSTMF<br>VLHSGNKATVNLKAATASPPEY*
SiGATA15	MGNAGTGTPLWRNGPPDKPVLCNACGSRWRTKGSLANYTPMHRKDDIDDDEPRVSKLKPPTSKMKSQKKKANLIITENGPFSSQSFRKMGDADPSNRSSDGSAISYSESCAPYGAVDA<br>SEISGSAQSHAWESLVPSRKRSCVTRLKPSPVEKLAKDLNSIMHEEQLSYLSGSSEEDLLYHSEAPVGSFETGSGSVLLRHPNSKSPDEESEASSIPADDKSHITSESYSGSASVVVHSGNKA<br>TVNLKA
SiGATA16	MLHQTLIPSFFAPYVSASSSPPFLHASDAATAPPAGSSVPVLLRGPAMPSLAHHHSPLDDGRTDALKCNNSFAPEETAEDAAAAAAAGALVEKDGFSVEDLLDLEEFGEPDKDGADNEE<br>APLPPPPAAAAEEKSNGDSQPLSVVTYELPPPPPEMVDLPAHDVEELEWVSRIMDDSLSELPPQPHPPAALVASLAARPPLAQQRRVPQPHVHDGAYRALPPAPGPLRTPTICALSTEALV<br>PVKAKRSKRSRAPGWSLSGASFLSDSASSSSTTTTSSCSSSGSFSPFLFLDSAPFSSGLELAEGYYNHFLPAPASKKSKHGGGKGSKHKPKKRGRKPKHLPPNPSAAGAVASQPAPGDRRC<br>SHCGVQKTPQWRAGPEGAKTLCNACGVRYKSGRLLPEYRPACSPTFVSTIHSNSHRKVLEMRRKKESGMVATAAPAVASF*
SiGATA17	MMDSSEQKVIGIAPAPAVAEAGRPCCVECRTTATPMWRGGPTGPRSLCNACGIRYRKKRRQELGLDNNQKPQQNHQQPPPQQQPQQQQHQDHSQAPNAVKDNKSSGLQVVKKRRVL<br>MGVEEAAILLMALSSSSRSTLLHG*
SiGATA18	MTPLSPSEQERHRETASPVPNAPPPPPQGSGPSKTQRPAVATAMMPGGGGTERAREGRLAGAVSWMAALLLAAVVAGRDAAVPRKKRGPRVRAETSRWWSFRLPVPPPPATPRAGTC<br>GDCGVTKTSQWRTGPMGPRTLCNTCGRRRWAAGEQWGEPRRRRRATTPTTVSDQPPPPPPDGPVWEGPLPEGYRTARRNAAKGSSPSPAPATATTDSDKPAPQNKKKNKAAAAAASE<br>KQCVHCGSSETPQWREGPEGPATLCNACGLRYRQRRLLPEYRPQASPTFDKENHASMHSEVLELRQQSKNKQKQQQPPALAQPQPMDDTQQDVDHLMPPPLPRCVANDLRVGATDG<br>DSANKASGSGAAVASTDPVGEASSLDPFLLDGPAAPMVVDEPCWMIAGSSTP*
SiGATA19	MAGGAGEGEREGPLGYVLSLPAASLPLPVAVSCLDATVPRKARSRLRLRVQPCAWWAFKLPVPAPEEAKSPAPPASMATNPTEEARSPRPQRLRVRHGPPPSPDDPHTPSPAMERPAKR<br>ARRCLQCGAVETPQWRSGPMGSGTLCNACGVRLKAAGALREQVHRPPPASARTVAEPPPESPVSDSSPDGPIWEPGSVPDVYLLRKKPPKQGKPPPPRMEPASPPAPAPAVYLLKKKKK<br>KPPKASKKKPWRPRKSSKRCLHCGSSSTPQWREGPMGRSTLCNACGVRYRQGRLLPEYRPLASPSFEPSEHANRHSQVLQLHRQRKGQKNQPPLPTEQPRLMDDLTGALACSGDGDDP<br>MNVLLPRRWHDKDEYPRTPLHQPLPQPADSLPGDPRVGGIDATAQGRGGRGNDPNGAPSSLDSLLLEGPSAPLIDDGDESLIE*
SiGATA20	MRTQSHLSLLDVPDDLPDCDGGCFRPGGGLCCPDDPLDLVLQLFPAAAPAPHEVSLTALGIGGSPRRQDQPPPFGWAQENGFGDVTVLGAGSAPGGGGVWERDSCGLSGRVPEHMEPL<br>DVDKYLVDHAPDDGGGEVTVCNPSRDTRGMPASGVRACGALGGVVSNYAPPLLAPMSAGALHPYASGDGLHACGAQRGAVSNDAPPMPAGALHACRALVHVVSDDAPPLRAHAPP<br>SARSLPASRTSSGSLTPTTSETSSPAPVWRPLAWPVPKKQRRPPVKCRKRPWSLDFPLHAVPVAPPDNPGDSNGNGDARNSFDNAGGGGIRRRRPVPRQRNRQAQRVCSHCHSPDTPQW<br>RAGPDGPGTLCNACGIRYAANKLLPEYRPSTAPSFRSDQHSNRHRKVVKLREQKAKETLKAMPDP

SiGATA21	VETILMLLNGYELVPQSAKPQLTHLVQPIVVPRDFDRTAAVSRYREKRKSTLKFDVKADYSIRREIASRIARRRGKFVSSDKSSDNSVAAAARRRQRESCAKCGESSEATPMMRRGPNG<br>YRTFCNACGLMWAKTNKIRKLTNPDCGAAAAGVAVAVAAEA
SiGATA22	MDSSVEKGSGSPDPDERPAAGEPKACTECHTTKTPLWRGGPCGPMSLCNACGIRYRKKRREAMGLDANKAAGGEQQQQQRKKKAAAAAASKREREKGAEADEVTVELRTVGFGK<br>EVVLKQRRRMRRRRRLGEEERAAILLMALSSGVVYA*
SiGATA23	MCTVHVRGSCPSLRTCAQTQPVRASPPSDPQPMAAEPAADDHDPRPTPADGPAAAGGGDASASAAAEALMSAASEQLTMVYQGDVFVFDPVPPQKVQAVLLVLGGYEVPSGLVNMT<br>VPTANDEKSTTVAARRVASLMRFREKRKERCFDKRIRYNVRKEVAQKMKRRKGQFAGRSDFGDGACSSVACGSPPNGEDDHFRETHCQNCGISSRLTPAMRRGPAGPRSLCNACGLM<br>WANKGTLRSPINAPKMTLQHPANPSKTGDTDDKNSTVLPVEHNQATVKTDSGMMLEQEQKLDILPPTKEDIKSVS*
SiGATA24	MDRTTLETSLDPSYTTTAPATATASRPSIPQSRVASSPPQPAMPDADVDAEMRDAAAAAAAPAGDDGEDDTGEEEDDEDDIDDEDEEEPTAPVPVPPVEPPVPAPVSVLPGNPNQLTLLF<br>QGEVYVFESVTPDKVQAVLLLLGRGELPPGSTGMVLPNQNENKGYDDILRRTDIPAKRVASLIRFREKRKERNFDKKIRYAVRKEVALRMQRRKGQFAGRASLEGESPAPGCDPGSQSS<br>GLDFVSRESKCQNCGTSEKMTPAMRRGPAGPRTLCNACGLMWANKGTLRSCPKAKVESPAVAIEQGGSDNKALMAPKNDNVSASNSEATSAAETGAPKAP*
SiGATA25	MVVENAPHGDSVAAVADELFFSAGAGVDLETFFDHAVRVAAAPCFRVFAVLGWEMRGLTQGRFLQALEVAAAGSSGAKGEEEELEWLSNKDAFPAVETMLPAAAPRPPTKGARRRR<br>RVVAKKAMAGRRCRHCGTEETPQWREGPEGAATLCNACGLRYRSGRLVPEYRPASSPTFSPEMHSNRHNRVVEMRRQRQVAAVASPGAAFGEKALGAVSSEALPKGERPAKRLRFR<br>QQSPANPALRSPFEEAEPKNLAPAAPPRLFPTLTLEEGPQAVEATAGRSGGCDGDGGGAQ
SiGATA26	MVVVEVLQGDSAAAVADDLFGSGADLQAFFDHAALDVKASGGGEEGEEELEWLSNKEAFPAVETMASSAPRQRTKGVPPPRWEVGWSPRQAPAVARPPAAGWRCRHCGTDKTPQR<br>REGPEGRSTLCNACGVRYRSGRLVPEYRPASSPTFSPELHSNRHSRVVEMRRRREAAAGASLAAAGKGEEKGNEKLECLSNKGEFLAVQMMAAARPRTEGTRRPRKAVDWPAIAWRP<br>PPPPRAPAVAARRPSQGGGVGVAVDQGRAPGGGDDGVGGCAAAYQGRTAVPADGGLNPAAPLAPAAAPGQQFGTEKTLQRLGGAEGRSTLCNACVVQYRSGCMVPVLPPACSPAFS<br>PELRFDWHNRVEMHRRRERSAKLPPATARAGEKGKEELEWPSNKGVFPAAQAMSPAAAGARPQTKGVRRQRQRQQLRRRRVVELSPPRTPPPLRRRSRCGGEAAAVEQGRVRDGGA<br>AADEVLAAAPAGGGKDPAAPAAAPRTPAVSRRRCRHCGTEKTPQWREGPEGRQTLCNACGVQYKSGRLVPEYRPASSPTFSPGLHSNCHRQVVQLRRRREESAEVSPAAAAVGDK*
SiGATA27	MASEWEMAMGVELGMGMGTYHGHHNASSITTAPMSSHHSGGASYSTAHHHHYYGMPPMGDAMRVDELLDLSTGAGAHDFFPTAAGAAAADNGHHSGAMGEPSPTANSSDHQTSL<br>LSFADEFYIPSEEAAELEWLSKFVDDSYSDMPNYSSHAAMAAAAAANAAGNGGGNSGGQDSCVTAAPGRGARSKRSRATAAAAAAWHSLVPRPPSQSSPSSSCSSSDFPSSNKPGRPN<br>GANGSRGKKSPGPGGAAGAEVGMEGGVRRCTHCASEKTPQWRTGPLGPKTLCNACGVRFKSGRLMPEYRPAASPTFVLTQHSNSHRKVMELRRQKELILIRGSHRDAAAAAAAASAA<br>AGSAGPRPELMFRDYGVC*
SiGATA28	MPAGESSSTERMGGRRETGSGARLRLMPVPAPNRKPSHDAQGSSQEKPTSSTDMWPASTKNLGSMSHERWSPYDQLAELEWLSNYMGKDNFPTEDLKKLQIITSVPPASSTDTAMVFV<br>SAPVVLPDGGVLPPEAPVPGKASRLLVLPPPPVSPPSPAFAAISPSESGTTAPAFPAKKPLKPAKKKEPSTTPEPNATTVATAASAGEGRKCLHCETNKTPQWRTGPLGPKTLCNACGVWY<br>KSGRLVLEYRLAASLTFVVSKHSNSHRKVLELHRQKEAMGHMGAGATGGLMHTPSPLLFDEPTGPLIGNDFLIHNRIGLDFRQLI*

Table S2. GATA protein sequence information from six representative species for phylogenetic tree analysis.

Table S2-1 Sequence and information of GATA proteins in *Arabidopsis thaliana*.

Gene id	group	Sequences
At1g08000	Subfamily I	MNWLPEAEAEEHLKGILSGDFFDGLTNHLDCPLEDIDSTNGEGDWVARFQDLEPPPLDMFPALPSDLTSCPKGAARVRIPNNMIPALKQSCSSEALSGINSTPHQSSAPPDIKVSYLFQSLTPVSVLENSYGSLSTQNSGSQRLAFPVKGMRSKRRRPTTVRLSYLFPFEPRKSTPGESVTEGYYSSEQHAKKKRKIHLITHTESSTLESSKSDGIVRICTHCETITTPQWRQGPSGPKTLCNACGVRFKSGRLVPEYRPASSPTFIPSVHSNSHRKIIEMRKKDDEFDTSMIRSDIQKVKQGRKKMV
At1g08010	Subfamily I	MNWLPEAEAEDDFKGLLSGDFFDDLINHLDVPLDDIDTTNGEGDWVDRFQDLEPPPMDMFPTLPSDLTSCGSGMAKAPRVDIQRNIPALKQSYSSEALSSTLHQSSAPPEIKVSKLFQSLSPVSVLENSYGSLSTHNNGSQRLAFPVKGMRSKRKRPTTLRLSYLFPSEPRKPEKSTPGKPESECYFSSEQHAKKKRKIHLTTRTVSSTLEASNSDGIVRKCTHCETTKTPQWREGPSGPKTLCNACGVRFRSGRLVPEYRPASSPTFIPAVHSNSHRKIIEMRRKDDEQFDSSMIRAVISRG
At1g51600	Subfamily III	MDDLHGSNARMHIREAQDPMHVQFEHHALHHIHNGSGMVDDQADDGNAGGMSEGVETDIPSHPGNVTDNRGEVVDRGSEQGDQLTLSFQGQVYVFDSVLPEKVQAVLLLLGGRELPQAAPPGLGSPHQNNRVSSLPGTPQRFSIPQRLASLVRFREKRKGRNFDKKIRYTVRKEVALRMQRNKGQFTSAKSNNDEAASAGSSWGSNQTWAIESSEAQHQEISCRHCGIGEKSTPMMRRGPAGPRTLCNACGLMWANKGAFRDLSKASPQTAQNLPLNKNEDANLETDHQIMITVANDISNSQ
At2g18380	Subfamily II	MMGYQTNSNFSMFFSSENDDQNHHNYDPYNNFSSSTSVDCTLSLGTPSTRLDDHHRFSSANSNNISGDFYIHGGNAKTSSYKKGGVAHSLPRRCASCDTTSTPLWRNGPKGPKSLCNACGIRFKKEERRATARNLTISGGGSSAAEVPVENSYNGGGNYYSHHHHHYASSSPSWAHQNTQRVPYFSPVPEMEYPYVDNVTASSFMSWN
At2g28340	Subfamily I	MNNDLWLPEEDFKGLPDNFLDNLVDPTNDVSVEDIETGDDEGDWDAKFQKLVPPPLDELMSLSYEFTCNGQRVQVQKHVPILKQSSSSEVFSTVDNSPPNVKVSKLLQSLSPVSVLKNTNGSGSPQNPNGDQKLAFLVKGIRSKRKRPTLLRVTFLKSFLLEMSQQFAPDESESSEISALKKRKKNKSRRLKCTHCETTTTPQWREGPNGRKTLCNACGIRFRSGRLVLEYRPAASPTFIPTVHSNLHKKIIYMRMKDNDQFDTRKIRAETSGPETRSRLRNFGRPMSYGQ
At2g45050	Subfamily I	MDVYGLSSPDLLRIDDLLDFSNEDIFSASSSGGSTAATSSSSFPPPQNPSFHHHHLPSSADHHSFLHDICVPSDDAAHLEWLSQFVDDSFADFPANPLGGTMTSVKTETSFPGKPRSKRSRAPAPFAGTWSPMPLESEHQQLHSAAKFKPKKEQSGGGGGGGGRHQSSSSETTEGGGMRRCTHCASEKTPQWRTGPLGPKTLCNACGVRFKSGRLVPEYRPASSPTFVLTQHSNSHRKVMELRRQKEVMRQPQQVQLHHHHHPF
At3g06740	Subfamily II	MLDPTEKVIDSESMESKLTSVDAIEEHSSSSSNEAISNEKKSCAICGTSKTPLWRGGPAGPKSLCNACGIRNRKKRRTLISNRSEDKKKKSHNRNPKFGDSLKQRLMELGREVMMQRSTAENQRRNKLGEEEQAAVLLMALSYASSVYA
At3g16870	Subfamily II	MSEGSEDTKTKLDSAGELSDVDNENCSSSGSGGGSSSGDTKRTCVDCGTIRTPLWRGGPAGPKSLCNACGIKSRKKRQAALGMRSEEKKKNRKSNCNNDLNLDHRNAKKYKINIVDDGKIDIDDDPKICNNKRSSSSSSNKGVSKFLDLGFKVPVMKRSAVEKKRLWRKLGEEERAAVLLMALSCSSVYA

At3g20750	Subfamily II	MEPELDLTLKLGLPNSTVETHLTLSPPTTTTDQGTNVVDGGEVINHRRGLLGDDEVIHNEPTRNNVEFNIRIYNYVFQQFVGAPNTLNFAPYPMPPSPAPAPETPPVSDEYV<br>LIDVPARRARRNNSTVMTNSWKENATPKRIRGCGGFCGGRIEGMKKCTNMNCNALNTPMWRRGPLGPKSLCNACGIKFRKEEERKAKRNVVIVLDD
At3g21175	Subfamily III	MDDLHGRNGRMHIGVAQNPMHVQYEDHGLHHIDNENSMMDDHADGGMDEGVETDIPSHPGNSADNRGEVVDRGIENGDQLTLSFQGQVYVFDRVSPEKVQAVLLLL<br>GGREVPHTLPTTLGSPHQNNRVLGLSGTPQRLSVPQRLASLLRFREKRKGRNFDKTIRYTVRKEVALRMQRKKGQFTSAKSSNDDSGSTGSDWGSNQSWAVEGTETQKP<br>EVLCRHCGTSEKSTPMMRRGPDGPRTLCNACGLMWANKGTLRDLSKVPPPQTPQHLSLNKNEDANLEADQMMEVTGDISNTQ
At3g24050	Subfamily I	MEMESFMDDLLNFSVPEEEEDDDEHTQPPRNITRRKTGLRPTDSFGLFNTDDLGVVEEEDLEWISNKNAFPVIETFVGVLPSEHFPITSLLEREATEVKQLSPVSVLETSSHS<br>STTTTSNSSGGSNGSTAVATTTTTPTIMSCCVGFKAPAKARSKRRRTGRRDLRVLWTGNEQGGIQKKKTMTVAAAALIMGRKCQHCGAEKTPQWRAGPAGPKTLCNAC<br>GVRYKSGRLVPEYRPANSPTFTAELHSNSHRKIVEMRKQYQSGDGDGDRKDCG
At3g45170	Subfamily I	MSGREDEEEDLGTAMQKIPIPVNVFDKEPMDLDTVFGFADGVREIIEDSNLLLEESREFDTNDSKPSRNFSNLPTATRGRLHAPKRSGNKRGRQKRLSFKSPSDLFDSKFGIT<br>DKSCSHCGTRKTPLWREGPRGAGTLCNACGMRYRTGRLLPEYRPASSPDFKPNVHSNFHRKVMEIRRERKSSPPNSFGFSESYHSTRKLGF
At3g50870	Subfamily II	MMQTPYTTSTQGQYCHSCGMFHHHSQSCCYNNNNNSNAGSYSMVFSMQNGGVFEQNGEDYHHSSSLVDCTLSLGTPSTRLCEEDEKRRRSTSSGASSCISNFWDLIHTK<br>NNNSKTAPYNNVPSFSANKPSRGCSGGGGGGGGGGGGDSLLARRCANCDTTSTPLWRNGPRGPKSLCNACGIRFKKEERRTTAATGNTVVGAAPVQTDQYGHHNSGYN<br>NYHAATNNNNNNGTPWAHHHSTQRVPCNYPANEIRFMDDYGSGVANNVESDGAHGGVPFLSWRLNVADRASLVHDFTR
At3g51080	Subfamily I	MESVELTLKNSNMKDKTLTGGAQNGDDFSVDDLLDFSKEEEDDDVLVEDEAELKVQRKRGVSDENTLHRSNDFSTADFHTSGLSVPMDDIAELEWLSNFVDDSSFTPYS<br>APTNKPVWLTGNRRHLVQPVKEETCFKSQHPAVKTRPKRARTGVRVWSHGSQSLTDSSSSSTTSSSSSPRPSSPLWLASGQFLDEPMTKTQKKKKVWKNAGQTQTQTQT<br>QTRQCGHCGVQKTPQWRAGPLGAKTLCNACGVRYKSGRLLPEYRPACSPTFSSELHSNHHSKVIEMRRKKETSDGAEETGLNQPVQTVQVVSSF
At3g54810	Subfamily I	MIGTSFPEDLDCGNFFDNMDDLMDFPGGDIDVGFGIGDSDSFPTIWTTHHDTWPAASDPLFSSNTNSDSSPELYVPFEDIVKVERPPSFVEETLVEKKEDSFSTNTDSSSSHS<br>QFRSSSPVSVLESSSSSSQTTNTTSLVLPGKHGRPRTKRPRPPVQDKDRVKDNVCGGDSRLIIRIPKQFLSDHNKMINKKKKKKAKITSSSSSSGIDLEVNGNNVDSYSSEQY<br>PLRKCMHCEVTKTPQWRLGPMGPKTLCNACGVRYKSGRLFPEYRPAASPTFTPALHSNSHKKVAEMRNKRCSDGSYITEENDLQGLIPNNAYIGVD
At3g60530	Subfamily I	MDVYGMSSPDLLRIDDLLDFSNDEIFSSSSTVTSSAASSAASSENPFSFPSSTYTSPTLLTDFTHDLCVPSDDAAHLEWLSRFVDDSFSDFPANPLTMTVRPEISFTGKPRSRRS<br>RAPAPSVAGTWAPMSESELCHSVAKPKPKKVYNAESVTADGARRCTHCASEKTPQWRTGPLGPKTLCNACGVRYKSGRLVPEYRPASSPTFVLTQHSNSHRKVMELRR<br>QKEQQESCVRIPPFQPQ
At4g17570	Subfamily IV	MIRTNGLGNKDMILGSEFVFMIRNCYTPLWRNGPPEKPVLCNACGSRWRTKGTLVNYTPLHARADGDENDDHHRFQRMKSISLGNKNKEIKMLKRKAIQENIIIKRPVFE<br>FSYGLKAAVIEEDASNRSSSGSAVSNSESCAQFSSADGSELTGPSQSNAWDTTVPCKRRTCVGRPKSSSVEKLTKDLYNILQEQQSSCLSVSSEEDLLFENEMSMVSVEIGH<br>GSVLMKNPHSFAREEESEASSLSSIENKSSISDAYSHSVKRVEIGAVRGSYYGGQTIKQEQFKRTKSQTERVHVLGSHGSPLCSIDLKDVFNFDEFIEQFTEEEQKKLMNLLP

		QIDSDDLPHSLRMMFESAQFKDNFSLFQQLIADGVFDVSSSSGAKLEEIRTFKKLALTDFNKSRLVESYNLLKEREKGTGDSVTTTSKSSIPNVPKNIVTIKRRYENQIQVKS<br>ESRGLMRSPKRVMKMKASHETENNVSCFRPRSLASVFAQEGGSAVFSYEGNCSSDQDLLLLDLPSNGSFPQAELLHQL
At4g24470	Subfamily III	MFGRHSIIPNNQIGTASASAGEDHVSASATSGHIPYDDMEEIPHPDSIYGAASDLIPDGSQLVAHRSDGSELLVSRPPEGANQLTISFRGQVYVFDAVGADKVDAVLSLLGG<br>STELAPGPQVMELAQQQNHMPVVEYQSRCSLPQRAQSLDRFRKKRNARCFEKKVRYGVRQEVALRMARNKGQFTSSKMTDGAYNSGTDQDSAQDDAHPEISCTHCGIS<br>SKCTPMMRRGPSGPRTLCNACGLFWANRGTLRDLSKKTEENQLALMKPVSSYKYHPDDGGSVADAANNLNTEAASVEEHTSMVSLANGDNSNLLGDH
At4g26150	Subfamily II	MGSNFHYTIDLNEDQNHQPFFASLGSSLHHHLQQQQQQQHFHHQASSNPSSLMSPSLSYFPFLINSRQDQVYVGYNNNTFHDVLDTHISQPLETKNFVSDGGSSSSDQM<br>VPKKETRLKLTIKKKDNHQDQTDLPQSPIKDMTGTNSLKWISSKVRLMKKKKAIITTSDSSKQHTNNDQSSNLSNSERQNGYNNDCVIRICSDCNTTKTPLWRSGPRGPKS<br>LCNACGIRQRKARRAAMATATATAVSGVSPPVMKKKMQNKNKISNGVYKILSPLPLKVNTCKRMITLEETALAEDLETQSNSTMLSSSDNIYFDDLALLLSKSSAYQQVF<br>PQDEKEAAILLMALSHGMVHG
At4g32890	Subfamily I	MEKIAPELFLVAGNPDSFVVDDLLDFSNDDGEVDDGLNTLPDSSTLSTGTLTDSSNSSSLFTDGTGFSDLYIPNDDIAELEWLSNFVEESFAGEDQDKLHLFSGLKNPQTTG<br>STLTHLIKPEPELDHQFIDIDESNVAVPAKARSKRSRSAASTWASRLLSLADSDETNPKKKQRRVKEQDFAGDMDVDCGESGGGRRCLHCATEKTPQWRTGPMGPKTLC<br>NACGVRYKSGRLVPEYRPASSPTFVMARHSNSHRKVMELRRQKEMRDEHLLSQLRCENLLMDIRSNGEDFLMHNNTNHVAPDFRHLI
At4g34680	Subfamily I	MELWTEARALKASLRGESTISLKHHQVIVSEDLSRTSSLPEDFSVECFLDFSEGQKEEEEEVVSVSSSQEQEEQEHDCVFSSQPCIFDQLPSLPDEDVEELEWVSRVVDDCSS<br>PEVSLLLTQTHKTKPSFSRIPVKPRTKRSRNSLTGSRVWPLVSTNHQHAATEQLRKKKQETVLVFQRRCSHCGTNNTPQWRTGPVGPKTLCNACGVRFKSGRLCPEYRPA<br>DSPTFSNEIHSNLHRKVLELRKSKELGEETGEASTKSDPVKFGSKW
At4g36240	Subfamily I	MECVEAFLGDFSVDDLLDLSNADTSLESSSSQRKEDEQEREKFKSFSDQSTRLSPPEDLLSFPGDAPVGDLEDLEWLSNFVEDSFSESYISSDFPVNPVASVEVRRQCVPVKP<br>RSKRRRTNGRIWSMESPSPLLSTAVARRKKRGRQKVDASYGGVVQQQQLRRCCSHCGVQKTPQWRMGPLGAKTLCNACGVRFKSGRLLPEYRPACSPTFTNEIHSNSHR<br>KVLELRLMKVADPARV
At4g36620	Subfamily II	MGFSMFFSPENDVSHHSSPYASVDCTLSLGTPSTRLCNEDDERRFSSHTSDTIGWDFLNGSKKGGGGGGHNLLARRCANCDTTSTPLWRNGPRGPKSLCNACGIRFKKEE<br>RRASTARNSTSGGGSTAAGVPTLDHQASANYYYNNNNQYASSSPWHHQHNTQRVPYYSPANNEYSYVDDVRVVDHDVTTDPFLSWRLNVADRTGLVHDFTM
At5g25830	Subfamily I	MEDEAHEFFHTSDFAVDDLLVDFSNDDDEENDVVADSTTTTTITDSSNFSAADLPSFHGDVQDGTSFSGDLCIPSDDLADELEWLSNIVDESLSPEDVHKLELISGFKSRPDP<br>KSDTGSPENPNSSSPIFTTDVSVPAKARSKRSRAAACNWASRGLLKETFYDSPFTGETILSSQQHLSPPTSPPLLMAPLGKKQAVDGGHRRKKDVSSPESGGAEERRCLHCA<br>TDKTPQWRTGPMGPKTLCNACGVRYKSGRLVPEYRPAASPTFVLAKHSNSHRKVMELRRQKEMSRAHHEFIHHHHGTDTAMIFDVSSDGDDYLIHHNVGPDFRQLI
At5g26930	Subfamily II	MDPRKLLSCSSSYVSVRMKEEKGTIRCCSECKTTKTPMWRGGPTGPKSLCNACGIRHRKQRRSELLGIHIIRSHKSLASKKINLLSSSHGGVAVKKRRSLKEEEQAALCLLL<br>LSCSSVLA

At5g47140	Subfamily IV	MGKQGPCYHCGVTSTPLWRNGPPEKPVLCNACGSRWRTKGSLVNYTPLHARAEGDETEIEDHRTQTVMIKGMSLNKKIPKRKPYQENFTVKRANLEFHTGFKRKALDEE<br>ASNRSSSGSVVSNSESCAQSNAWDSTFPCKRRTCVGRPKAASSVEKLTKDLYTILQEQQSSCLSGTSEEDLLFENETPMLLGHGSVLMRDPHSGAREEESEASSLLVESSKS<br>SSVHSVKFGGKAMKQEQVKRSKSQVLGRHSSLLCSIDLKDVFNFDEFIENFTEEEQQKLMKLLPQVDSVDRPDSLRSMFESSQFKENLSLFQQLVADGVFETNSSYAKLED<br>IKTLAKLALSDPNKSHLLESYYMLKRREIEDCVTTTSRVSSLSPSNNNSLVTIERPCESLNQNFSETRGVMRSPKEVMKIRSKHTEENLENSVSSFKPVSCGGPLVFSYEDND<br>ISDQDLLLDVPSNGSFPQAELLNMI
At5g49300	Subfamily II	MLDHSEKVLLVDSETMKTRAEDMIEQNNTSVNDKKKTCADCGTSKTPLWRGGPVGPKSLCNACGIRNRKKRRGGTEDNKKLKKSSSGGGNRKFGESLKQSLMDLGIRK<br>RSTVEKQRQKLGEEEQAAVLLMALSYGSVYA
At5g56860	Subfamily II	MDSNFHYSIDLNEDQNHHEQPFFYPLGSSSSLHHHHHHHHHQVPSNSSSSSSSISSLSSYLPFLINSQEDQHVAYNNTYHADHLHLSQPLKAKMFVANGGSSACDHMVPKK<br>ETRLKLTIRKKDHEDQPHPLHQNPTKPDSDSDKWLMSPKMRLIKKTITNNKQLIDQTNNNNHKESDHYPLNHKTNFDEDHHEDLNFKNVLTRKTTAATTENRYNTINENG<br>YSNNNGVIRVCSDCNTTKTPLWRSGPRGPKSLCNACGIRQRKARRAAMAAAAAAGDQEVAVAPRVQQLPLKKKLQNKKKRSNGGEKYNHSPPMVAKAKKCKIKEEEE<br>KEMEAETVAGDSEISKSTTSSNSSISSNKFCFDDLTIMLSKSSAYQQVFPQDEKEAAVLLMALSYGMVHG
At5g66320	Subfamily I	MEQAALKSSVRKEMALKTTSPVYEEFLAVTTAQNGFSVDDFSVDDLLDLSNDDVFADEETDLKAQHEMVRVSSEEPNDDGDALRRSSDFSGCDDFGSLPTSELSLPADDL<br>ANLEWLSHFVEDSFTEYSGPNLTGTPTEKPAWLTGDRKHPVTAVTEETCFKSPVPAKARSKRNRNGLKVWSLGSSSSSGPSSSGSTSSSSSGPSSPWFSGAELLEPVVTSER<br>PPFPKKHKKRSAESVFSGELQQLQPQRKCSHCGVQKTPQWRAGPMGAKTLCNACGVRYKSGRLLPEYRPACSPTFSSELHSNHHRKVIEMRRKKEPTSDNETGLNQLVQ<br>SPQAVPSF

Table S2-2 Sequence and information of GATA proteins in *Glycine max*

Gene	Gene id	group	Sequences
GmGATA1	Glyma01g10390	I	MVIANYGFLEHPLCVPQDSLECLGMMNWEGMDSIDSMFSTPWESEKERLEQPEKDKTERKSFTDSGRDAKIWEKRCGHKDARIWERRCSHCDAIKTPQ<br>WRTGPFGRNTLCNACGIRFKAGKLYPEYRPADSPTFDVSKHSNVHKEIMKMRNHLS
GmGATA2	Glyma01g37450	I	MLYQTPYPQPFQFHHPLPSSFSPLLAVPTTPPPLYLPFPQAEKEMECVEAALKSNYRKEMTLKLSPQTFTEEVSVQNGTTCDDFFVNDLLDFSHVEEEPEQ<br>QEDTPCVSLQHENPSHEPCTFKDDYASVPTSELSVLADDLADLEWLSHFVEDSFSEFSAAFPTVTENPTACLKEAEPEPEIPVFSFKTPVQTKARSKRTRNG<br>LRVWPFGSPSFTDSSSSSTTSSSSSSSPSSPLLIYTQSLDHLCSEPNTKKMKKKPSSDTLAPRRCSHCGVQKTPQWRTGPLGPKTLCNACGVRFKSGRLLPE<br>YRPACSPTFSSELHSNHHRKVLEMRQKKETVSVDETGFAPAHVVPSF
GmGATA3	Glyma01g41370	IV	MGKQGPCYHCGVTSTPLWRNGPPEKPVLCNACGSRWRTKGTLAKYTPLHARAETDDYDDQRVSRVKSISINKKKEVALLKRKQNHDNVVSGGFAPDY<br>NQGYQKVVDEDISNRSSSGSAISNSESCAQFGYGGMDASDLTGPAQSVVWDAMVPSRKRTCVGRPKPSSVEKLTKDLCTILHEQQSYFSVSSEEDLLFES

			DTPMVSVEIGHGSILIRHPSYIAREEESEASSLSVDNKQCPMSEAYSFSGAIAMHNDSSRLKSSSLEVEKIGNSTGQGMQQEQLKSDKSQLERVQILGNHESPLCSIDLNDVVNYEEFLRILTNEEQQQLLKLLPVVDTAKLPDSLEVMFSSSQFKENLTYFQQLLAEGVFDISLLGAKSEDCKILKRLALSNLSKSKWVAHHNFLKKCKNKAGKSNTMGSTGTTSTNVLNNRASTDVANIKRMRDSRNQNLPEIKTIMRSPKRTIAKASCEGKEAVEDGACYSPKHLFALPPDASFLLLDSLNFVQESSDQDLLLEVSSNTSFPQAELLQPTLSLGAQASTSSSSIYSNLVHH
GmGATA4	Glyma02g05710	I	MKERGTFQPLFNALPNSLIILQSFNFIPSLLSTTPSSFPSFLLSQAEKEMECLEAALKSSFRKDMALKQTLFLEEFSSASNVQNVVASSDDLFVDDLLNFSLLENNTNNNNNNNEEPDQQLNNHDSTTPQNNQENYNYNPSFNDNNFNTELTVPAEEEVADLEWLSRFVEDSNFSEYSLPFPATVTEKVKVKSPEPGNTAFTFKTPVPAKARSKRTRTGVRVWPLKSPSLAAASSTTTSSSSSSSPSSPQRADSRAKKRAAADGGAARRCSHCGVQKTPQWRTGPLGAKTLCNACGVRYKSGRLLPEYRPACSPTFSSELHSNHHRKVLEMRRKKEDVPEPDTASPPSLPGF
GmGATA5	Glyma02g06320	II	MMHHCCGSSQGHVMGTCTCGMYHNHNSSEASSYGSMLFSMPNNNEYYQEHDIYSSFTPSHSSVDCTLSLGTPSTRLTQDEDDNKRHRHQRRSGVTSFCWDLLHSNHNNNNITQSQSKSSSRGSNNNNDSLLARRCANCDTTSTPLWRNGPRGPKSLCNACGIRFKKEQRRASAAGATSASAAVPGGAMESARVYGHHHNNSWYAHSQSQKMMGNELRFMDDSDDRDSDNNGIPFLSWKLNIPDRTSLVDERW
GmGATA6	Glyma02g07850	I	MDVCRNVSVSSSECQQELPTLDDLFSHQNTEVDFGLEWLSVFVEDCFSSRPSCLLAPGGVQTTSTSTSTKPSSGTILQRPQQLSHHCPLQNFAVPGKARSKRKRKRLSAPRTTKHTLSTWSQHFSTQNDGVSSDPPLLKQAYWLADSELIVPKKKDVEQEEEEGVVVVVKKEKLGDYCDHDEGDEINNNNSNNDDNVQHPIPRRCTHCLAQRTPQWRAGPLGPKTLCNACGVRFKSGRLLPEYRPAKSPTFVSYLHSNSHKKVMEMRMGVVGVFSTDNNK
GmGATA7	Glyma02g08145	I	MIGNFIDDIDCGSFFDHIDDLLEFPDDNAAPVAPPANFWSAESDSLPASYTVFSDNSVTDLSAELSVSYDDIVQLEWLSNFVEDSFSGGSITMKKEEEPQCTTTTKEDIAHAQFQTASPVSVLESSSFCSGEKAASRGPEIYIPVPCGRVRSKRPRPATFNPHPVMQLISPASSTGENVQHNATTTSKAASSDSENFAESVIKGPKQASGEHKNKRKIKVTFSSGQEQQNAPSQAVRKCLHCEITKTPQWRAGPMGPKTLCNACGVRYKSGRLFPEYRPAASPTFCAAVHSNSHKKVIEMRNKTGTKSGFATDSAASPELIPNTNNSLTLEYM
GmGATA8	Glyma02g37980	III	MEPSAMYGPSQPLNIPSRIGAGERDDGSGNEPAVDGHHHHIQYETHALDDGAAGGAVVVEDVTSDAVYVSGGGGPEESSQLTLSFRGQVYVFDAVTPDKVQAVLLLLGGCELSSGGSPCVDPGAQQNQRGSMEFPKCSLPQRAASLDRFRQKRKERCFDKKVRYSVRQEVALRMHRNKGQFTSSKKQDGANSYGTDQDSGQDDSQSETSCKHCGTSSKSTPMMRRGPSGPRSLCNACGLFWANRGALRDLSKRNQEHSLPPVEQVDGGNDPDCRTAAADPAQNNLAAFSEPVNPALVADRKVFQSQKMLE
GmGATA9	Glyma03g27250	I	MDLYGSFSTPSDCLHIDDFLDFSNITTTTTDTHHHFPPPQNSPSISHDPNFFLNFPSVPSDEAVELEWLSQFVNDEATSFHNIPPPASIGSHTTPFLSNNNRNDNNNEYPKSSSSSPVLAGKSRARREGSVTGDGVRRCSHCATDKTPQWRTGPLGPKTLCNACGVRFKSGRLVPEYRPAASPTFVMTQHSNSHRKVMELRRQKELLRHQQQEQCYRHTHHDFKVC
GmGATA10	Glyma03g39220	II	MMDLNVNEKKKCCADCKTTKTPLWRGGPAGPKTLCNACGIRYRKRRACSRKREEQRWKMLGEEEQAAVCLMALSSGFVFA

GmGATA11	Glyma04g01090	I	MEVAVAKALKPSLRREFIVQQMLCEDIFSLNANTVAAGEDFSVDDLFDFSNGSLHNEQQQEYDEGKQSLSASEDRGEDDCNSNSTGVSYDSLFSTELAVPAGDLEDLEWVSHFVDDSLPELSLLYPVRSEEANRFVEPEPSAKKTPCFPWEMKITTKARTVRNRKPSNSRMWSLGSPLLSLPSSPSSPSSCSSSVREPPAKKQKKQAQAQPVGAQIQRRCSHCHVQKTPQWRTGPLGAKTLCNACGVRYKSGRLFSEYRPACSPTFCSDIHSNSHRKVLEIRKRKEVAQPDTGLAQAQTQMVPTC
GmGATA12	Glyma04g05431	II	MTSVSLNPNPPCPTIQDQSQLFISANNHESTSLSCCTFFHILDQSQTKDIRDLRHGHQQDGKLVFHIGPSNNNNQVCNSSSVKLQPKPVKADSSSECGHHNVSLYKIEDEENKRDHDYEKWMSSTARLTRKMMRLPSTSSDLATKKALNNITRVCADCNTTSTPLWRSGPNGPKSLCNACGIRQRKARRAMAEAVNGFAPSVNSSSTKIRVHHKEKKSRTNHFARFRLKCKLATTSTAEGTSQQENVKIDLNDFGLSLRDSSALKQQVFPIMDEVAQAAMLLMDLSCGFVYC
GmGATA13	Glyma04g08990	I	MEAQEFFQNTFCPQFPSDSNITPSNANPSAATTDHFLVEDFFDFSNDDDATFDSLPTDVDSPTVTPVDTTTNSNFPASADAHFPGDLSVPYDDLAELEWLSKFADESFSSEDLQKLQLITGVRAQNDAASSETRDPNPVMFNPQVSVRGKARSKRTRGPPCNWTSRLVVLSPNTKSSSSSHSGAEGGSEGRKCLHCATDKTPQWRTGPMGPKTLCNACGVRYKSGRLVPEYRPAASPTFVLTKHSNSHRKVLELRRQKEMVKVQHQQHQFLQLQHQQNMMFDVPSSNGEDFLIHQHVGPNFTHLI
GmGATA14	Glyma04g10330	III	MFGSINQIVSAEDTDGPVSDHHIHYSSHTIEDDGAASDHHIHYSSHTIEEDGAASDHHIHYSSHTIEDDGAAVEDVSAVPGPEISIDNSSQLTLSFRGQVYVFDAVTPDKVQAVLLLLGGNELTSGSQCAELSSRNQTGEEEYPAKCSLPHRAASLNRFRQKRKERCFDKKVRYSVRQEVALRMHRNKGQFTSSKNQDGTNSWGSDQESGQDAVQSETLCCTHCGISSKSTPMMRKGPSGPRSLCNACGLFWANRGTLRDLSKRNLEHSLTPPEQVDEGSNNNALDIRSGIPAQHNNLVNDSKALVSDR
GmGATA15	Glyma04g10340	III	MATVNPQPLQFEDPAIPVDDDDDDDDDGGDDDAMDELEDANVNSVNVTNAASVNHEAVVAMPSRTSELTLSFEGEVYVFPAVTPQKVQAVLLLLGGRDVQAGVPAVEPPFDQSNRDMGDTPKRSNLSRRIASLVRFREKRKERCFDKKIRYSVRKEVAQRMHRKNGQFASLKESPGSSNWDSAQSSGQVGTSHSESVRRCHHCGVGENNTPAMRRGPAGPRTLCNACGLMWANKGTLRDLSKGGRNLSVEQSDLDTPIDVKPTSVLEGELPGIHDEQGSSEDPSKSNAADGSSNHAVNPSDEELPETAEHFTNVLPLGIGHSSTNDSEQEPLVELSNPSDTDIDIPGNFD
GmGATA16	Glyma05g05320	IV	MGKQGPCYHCGVTSTPLWRNGPPEKPVLCNACGSRWRTKGTLANYTPLHARAENVDYEDQKVSRVKSISLNKNKEVKLAKRKQNYDNAASGGFVPDYSQGYQKVVDEDTSNRSSSGSAVSNSESCAQFGGTDASDLTGPAQSVVWDAMVPSKKRTCAGRPKPSSVEKLTRDLCTILHEQQSYFSASSEEDLLFESDTPMVSVEIGHGSILIRHPSSIARDEESEASSLSVDNKQCLMNEAYSFSSTIPMYSDRSGMNFSSHGVEKIKNSAGQIMKQEKLERDKSQLEKLQVPGNHDSPLCSIDLNDVVNYEEFMRNLTNEEQQQLLKYLPVVDTAKFPDSLRNMFNSFQFKENLIYFQQLLGEGVFDISLLGAKPEEWKTLKRLALSNLSKSKWVEHYNFLKKCENKSGKSIGLGSTAMESSYVTTAKRMREHDSQNQNFPELKTTMRSPKRVFIKPSCEVKEVVEEGSSFSPKSLFALPHGVGSLHMLDSFNFVGESSEDLLLEVPSNSSFPQAELLHPSLSYGAQVSTTSSSVHSLVTHP

GmGATA17	Glyma05g30385	IV	MAKRNGPCFHCGIKSSPHWRSGPEDKSVLCNACGLRYTKWGSIGLQNYFPNHFKPEYLDNLKNLEGRNNVLQGSSYATDSSGKIHVMWNPYVPSRKRS RVVRMTTSIQRFHEQLLMMWKNEENSNDQSSQESEEVLLIDNVNNFIPCNEIGLGCILLKPEDASA
GmGATA18	Glyma05g30390	IV	MGRKHGPCFHCKIHITPLWRNGPEDKPVLCNACGSRYRKCGSLENYLPNHFQPEYPDNLKMLKRRKTLKGGKGRYLCSPKIPTRKRSPLVRKKITPMKR FYMQLQNMWEDYGNSNESSSEEVLIFNNVNNFIPSNEIGLGCIPLKLDDASA
GmGATA19	Glyma06g01110	I	MEVAVAKALKPSLRSEFIVQKMHCEDIFSLNANTVAVGEDFSVDDLFDFSNGSLHNEHQQECDEEKQSLSASSQSQDRGEDDSNSNSTGVSYDSLFSTEL AVPAGDLEDLEWVSHFVDDSLPELSLLYPVRSEEANRFVEPEPSVKKTPRFPWEMKITSKARSVRNRKPNTRVWSLGSTLLSLPSSPPAKKQKKRAEAQV QPVGVQIQRRCSHCQVQKTPQWRTGPLGAKTLCNACGVRYKSGRLFSEYRPACSPTFCSDIHSNSHRKVLEIRKRKEVAEPDTGLAQTQMVPTC
GmGATA20	Glyma06g09080	I	MEAQEFFQNTFCPQFPSGTNITPSNANPSAATADHFLVEDFFDFSNDDNDATAVTDATFDSLPTDVDSPNVTPLDSTTKNSNLPSSSSADAHFSGDLSVPY DDLAELEWLSKFAEESFSSEDLQKLQLISGVRAQNDAASSETRDPNPVMFNPQVSVRGKARSKRTRGPPCNWTSRLVVLSPNTTSSSSNSDAGKKPATPR RREAAFAEGGSEGRKCLHCATDKTPQWRTGPMGPKTLCNACGVRYKSGRLVPEYRPAASPTFVLTKHSNSHRKVLELRRQKEMVKVQQHQFLQLHQQ NMMFDVPSSNGEDYLIHQHVGPDYTHLI
GmGATA21	Glyma06g10280	III	MFGSMNKIVSAEDTDGSVSDHHIHYSSHTIEDDGAASDHHIHYSSHTIEEDGAVSNHHIHYSSHTIEEDGGATVEEVSAVPPLEISINDSSQLTISFRGQVYV FDAVTPDKVQAVLLLLGGNELTSGSQCAELSSQNQTGEEEYPAKCSLPQRAASLNRFRQKRKERCFDKKVRYSVRQEVALRMHRNKGQFTSSKNQDGT NSWGSDQESGQDAVQSETLCTHCGISSKSTPMMRRGPSGPRSLCNACGLFWANRGTLRDLSKRNQEHSLAPPEQVDEGSNNNDFDCRSGIPAQHNNLV NDNKA
GmGATA22	Glyma06g10290	III	MATVNPQPLQFEDPAIPVDDDDDDDDGGDDDAMDDLEDANVNSVNVAANAAASVNHEAVVAMPSRTSELTLSFEGEVYVFPAITPQKVQAVLLLLGG RDVQARVPAVEQPFDQSNRGMGDTPKRSNLSRRIASLVRFREKRKERCFDKKIRYSVRKEVAQRMHRKNGQFASLKESPGSSNWDSAQSSGQDGTSHS ESVRRCHHCGVSENNTPAMRRGPAGPRTLCNACGLMWANKGTLRDLSKGGRNLSVEQSDLDTPIDVKPTSVLEGELPGIHDEQDSSEDPSKSNAADGSS NHAVNPSDEELPETAEHFTNVLPLGIGHSSTNENEQEPLVELSNPSDTDIDIPGNFD
GmGATA23	Glyma07g01960	I	MREKFHLCHSFTPQNPTISVSHSKPKQRNPHSFHQNGALRSLSLKPTHTTSLQLLPLKHTIYMEVPEYFVGSFFGTGGAEQFCPPEKRHSDQKTGEPFAIDD LLDFSHADAIMSDGFFDNVAGNSTDSSTVTAVDSCNSSISGSDNRFATTIVPRGFPSDPQFSGELCVPYDEMAELEWLSNFVEDSFSAEEELKTLQLLSGA AAASTAIGAKPQTPESSSSTDTLPPFASDDTLRNAPFLHSETPLPGKARSKRSRAAPGDWSTRLLHLVATEQEKLPQLKAEPAKKREGTNAECSGRKCLH CGTEKTPQWRTGPMGPKTLCNACGVRFKSGRLVPEYRPAASPTFMSTKHSNSHRKVLELRRQKELQRQQHQQLMSQSSIFGVSNGGDEFLIHHHHQHC GPDFRHVI

GmGATA24	Glyma07g14750	I	MDLYGSFSTPSDCLHIDDFLDFSNITTDTHHHLPPPQNSPLISHDDANLFFNFPSVPTDEAAELEWLSQFVDDDATSFHSFPATASIGSHSTSFLSNNNNRNDNNEYPKSSLSSNIPCSSAVAGKSRARREGSVTGDGGVRRCSHCASEKTPQWRAGPLGPKTLCNACGVRFKSGRLVPEYRPAASPTFVLTQHSNSHRKVMELRRQKELLRHQQQQQLQQEQCHRHTHNHHDFKVC
GmGATA25	Glyma07g30140	III	MDNSLNNPSDNGEDPPPPPPVPMQVDGFQPFHYANCSDEGEEAVPVTNASSAMHARASELTISFEGEVYVFPAVTPEKVQAVLLLLGAQEMTNSAPTSDILLQQNYQDIREINDPSRSSKLSRRFASLVRFREKRKERCFEKKIRYSCRKEVAQRMHRKNGQFASMKEDYKSPAENWDSSNGTPCPESTERRCQHCGISEKSTPAMRRGPAGPRSLCNACGLMWANKGTLRDLSKAARIAFEQNELDTSADIKPSTTEAEHSFAKQDKEGSPEETKPVQMDSSRSPEKTNDQFIIGTAESVTDNLSIQVENHALSLHEQDTLEDLADASGTEFEIPAGFDDQVDIDDSNMRTYWL
GmGATA26	Glyma07g37190	II	MVDPTGKGSEIEVEDSNSNPNAPSSGNSPSSNNEQKKTCADCGTTKTPLWRGGPAGPKSLCNACGIRSRKKKRAILGINKGSNEDGRKGKRTGGALGKEVLLHRSHWKKLGEEEKAAVLLMSLSYGSVYA
GmGATA27	Glyma08g07170	III	MDNSTLNNPGDNAEDPPPPPAPMQVFDSFQPFHYANGSDEGEEAVPAPVANASSAMRARASELTISFEGEVYVFPAVTPEKVQAVLLLLGAQEMPNSAPTSDFLLQQNYQDIREINDPSRSSKLSRRFASLVRFREKRKERCFEKKIRYSCRKEVAQRMHRKNGQFASLKEDYKSPAENWDSSNGTPCPDSTERRCQHCGISEKSTPAMRRGPAGPRSLCNACGLMWANKGTLRDLSKAGRIAFEQNELDTSADIKPSTTEAKHSYAKQGKEGSPEETKPVQMDSRRSPEKTNEQFIIGTAESVTNNLSVRLENHALILHEQDTLEDLADASGTEFEIPAGFDDQVDIDDANMRTYWL
GmGATA28	Glyma08g15061	II	MWYVSQPNHQLLRHVFLHAQPQTTTLHDSDNIYDYSSFTPSSFSSVDCNLSLGTPSTCVSEDEEKRSRHECHSVSNFCWDLLQSKHNNPQSHSKSSGTTNTTDPLLARRCANCDTTSTPLWRNAPVALRYVNNYNI
GmGATA29	Glyma08g19681	IV	MKKKGPCSHCRISYTPLWPNGPADKPVLCNACGSRYKTRGHLDNYLPKNVHPQPHHKKFKNVNSGGSNLNVEPELESGNQLLNHVSPRSTTNGDSDKLTLDVHHISPQDFGKKIPSKKRSPMVYKRMIPMEKFQKQLVKLYKSERQPEESVLVDNMMNFIPENEIGLGTILLKTNDDDASSTDKCGSSTSAP
GmGATA30	Glyma08g21630	I	MEAPEYFVGGYFGAGGAEQFSLSEKRHSDQKTGEPFAIDDLLDFSHADAIMSDGFFDNVTGNSTDSSTVTAVDSCNSSISGSDNHFATAIVPRCYHSDPQFSGELCVPYDEMAELEWLSNFVEDSFSAEEELKTLQLLSGGGAASTAIGAKPQTPESSSSTDTLPPFASRRTLRNAPFLHSETPRPGKARSKRSRAAPGDWSTRLLHLVAPEKEKPPQAKKREGTNVECSGRKCLHCGAEKTPQWRTGPMGPKTLCNACGVRFKSGRLVPEYRPAASPTFMSTKHSNSHRKVLELRRQKEMQRQQHHQQLMSQSSIFGVSNGGDEFSIHHHHHNHHLHCGPDFRHVI
GmGATA31	Glyma08g23720	III	MDGIHGGDSRIHITDGQHPIHVPYVQEHEHHGLHHISNGNGIDDDHNDGGDTNCGGSESMEGEVPSNHGNLPDNHAVMMDQGGDSGDQLTLSFQGQVYVFDSVSPEKVQAVLLLLGGREIPPTMPAMPVSPNHNNRGYTGTPQKFSVPQRLASLIRFREKRKERNYDKKIRYTVRKEVALRMQRNKGQFTSSKSNNDESASNATNWGMDENWTADNSGSQQQDIVCRHCGISEKSTPMMRRGPEGPRTLCNACGLMWANKGILRDLSRAAPLSGTIKNENKSLEANQIVHRVAGEADDSS

			MALVGEQKHSLMVKKGPCSHCGVTHTPLWHDGPTEKPVLCSDCGSQYKLKGNLDNYFPKNPVVQSFHNKFTNVNGGKHLNVDVDQLSNYVPPTDED
			NNMSTPNVHCISAQATPLWRNGLVDKSVLHKACELSPQETPASAINAPKKLQLQPLIHKNFINVNGGSSLNVEDEDQLSNHITPASDGDNNKSTPNVQHIS
GmGATA32	Glyma08g45835	IV	PQDFGSKIPSRKRSRVVYLTPLKECMEELWKLHRNYGRHAEERILEDNVNNFIPENEIAGLGAILLKTDHDVAASADTCESSTDD
			MIPTYRYSVSSPMPIDLNEDHTHHVFSTSHQASSSSSSLSFSILFNPDHQGQGGSCCHWESKHLQSDEEAQKIVPSSESWEHPVSEKDENRSDLKLRVWKK
			EDKCENFQVEDNSTKWMPLKMRMMRRMMVSDQTGFDTEGMISNSKQIKNEEKNPPLTPLGTDDSNNYNSSANHSKITVRVCSDCHTTKTPLWRSGPK
			GPKTLCNACGIRQRKARRAIAVAATANGMNPVEAEKSQVKKGNKLHSKGMKSKTKGAPHMKKKRKLGAKYRKRFGAFEDLTVRLSKNLALQKVFPP
GmGATA33	Glyma09g07090	II	DEKEAAILLMALSLWPSSWLSHRSLRQLLRFMRYIGGKTIIIV
			METIGSVDDLLDFSSDIGEEDDYDDKPRKACPSLNSKCAGPSLFNPLVQVDPNHSFSEFAEEELEWLSNKDAFPSVETFVDLSSIQPGTTKNQKSAPVLECS
			TGSSNSNNSTNSISLLNSCDHLKVPVRARSKSRSRHRPGLAENSSQQVWWRQPSNGTSKADEGMKISSIGRKCQHCGAEKTPQWRAGPSGPKTLCNACG
GmGATA34	Glyma10g25480	I	VRFKSGRLVPEYRPASSPTFHSDLHSNSHRKIVEMRRQKQMGMG
			MVGPNFMDEIDCGSFFDHIDDLLDFPVEDVDGGAATLPSVSAGNSNSLASIWPSESDSFPASDSVFSGNSASDLSAELSVPYEDIVQLEWLSNFVEDSFCG
			GSLTMNKVEEPSCTTKEDSVNTQFHTSSPVSVLESSSSCSGGKTLPPRSPEIYIPVPCGRARSKRPRPATFNPRPAMNLISPASSFVGENMQPNVISSKASSD
			SENFAESQLVPKMPKLASGEPKKKKKVKVPLPVAPADNNQNASQPVRKCMHCEITKTPQWRAGPMGPKTLCNACGVRYKSGRLFPEYRPAASPTFCPS
GmGATA35	Glyma10g35470	I	VHSNSHKKVLEMRCRGFDKSGFAINSAASPELIPNTNSSLTLEYM
			MGKQGPCYHCGVTSTPLWRNGPPEKPVLCNACGSRWRTKGTLANYTPLHARAETDDYDDQRVSRIKSISINKKKEVALLKRKQNHDNVMSGGFAPDY
			NQGYQKVVDEDISNRSSSGSAISNSESCAQFGYGGMDASDLTGPAQSVVWDAMVPSKKRTCVGRSKPSSVEKLTKDLCTILHEQQSYFSASSEEDLLFES
			DTPMVSVEIGHGSVLIRHPSYIAREEESEASSLSVDNKQCPMSEAYSCSGGILMHNDSSRLKSSSLEVEKIGNSTGQGVLQEQLKSDKSQHERVQILGNHES
			PLCSIDLNDVVNYEEFLRILTNEEQQQLLKLLPVVDTAKLPDSLKVMFNSSQFKENLTYFQQLLSEGVFDISLLGAKPEDCKTLKILALSNLSKSKWVEHH
			NFLKKYKNKAVKSNTMGSTGTASINVLNNRASTNVANIKRMCDSRNQNFPELKTIMRSPKRMITKASFECKEAVEDGACYSPKHLFALPPDASSLLLDSF
GmGATA36	Glyma11g04060	IV	NFVEESGDQDLLLEVPSNTSFPQAELLHPTLSLGAQASTGSSSVYSNLVHH
			MHRCCSGSQGHVMGPCTCGMFHSQTTSSFAMFFSMPNHKPPPYDDSDNIYDYSSFTPSSSSSVDCTLSLGTPSTRFSEDEEKRSRHERRSVSNFCWDLLQS
			KHNNPQSHSKSSRTTNTTDPLLARRCANCDTTSTPLWRNGPRGPKSLCNACGIRFKKEERRASAAAATPASAASGGVMESAQVYNNSWYAHQQSQKM
GmGATA37	Glyma11g07350	II	QCFSPGMGNEFRFVDDADRDAADNGIPFLSWRLNVTDRTSLVHDFTR
			MEVAAAKALKPSLRTEFIFPQAIYDEILCFNANNVVADEDFSMDDLLDFSNGEFQVGKDFDDYEEEEDEEKNSTSGSLQSQDRAEDDNNSNSTAGGGGH
GmGATA38	Glyma11g11930	I	DYVFAGELSVPADDVADLEWVSHFVDDSLPELSILYPIHCSKKTRVWAEPESRLSPAQTVSKVPRKSRTEKPRKPNTRVWSSFTVFAGSVGFGELVTKKQ

			KKKVEAQSGGAQSLRRCSHCQVQKTPQWRIGPLGPKTLCNACGVRFKSGRLFPEYRPACSPTFCGHIHSNNHRRVLEMRWKKQIAESVTGSDRKQLIPNY
GmGATA39	Glyma11g20480	I	MKDCWFFDNNFNGLSDESLDDVMDMELLDLPLDFEDVETDAVEEQDWDAQLKLLEDPPPPLGVFPLQQSSAFCGQTRNENAKLGSKSFSASLAKTVRPAYGKTIPVQKVSLKGKDLLQFQTNSPVSVFESSSSSPSVENSNFELPVIPTKRPRTKRRRLSNISLLYSIPFILTSPAFQKFQRMDFSKSDIQTQPSGELLCKFKKKQRKKDIPLPTNKIEMKRSSSQESVAPRKCLHCEVTKTPQWREGPMGPKTLCNACGVRYRSGRLFAEYRPASSPTFVASLHSNSHKKVLEIRNRATQVTVR
GmGATA40	Glyma11g25375	II	MTLITPSSSSSVDCTLSLGTPSTRFSKYEEKRSCHERRSVSNFCWDLLQSKHNNPQSHSKSSQITNTTDPVLVHRCANCDTTYNPLWRNGPHGPKSLCNACGIRFKKEERRASAATAKMQCFSPRMGNEFRFMDDADRVTADNGISFLSWRLNVTD
GmGATA41	Glyma12g04180	I	MEVAAAKALKPSLRTEFIFPQAIYDEILCFNANNVVAGEDFSVDDLLDFSNGEFQVGKDFDDYEEDEDEEKGSTSGSLQSQDRTEDDSNSNSTAGGGGDSVFAGELSVPADDVADLEWVSHFVDDSLPELSLLYPVRCSEQTRVCTEPEPRPGSVQTIPAVPRKPRTGKTRKPNARVWSSMSSLCSSVTAKKQKKKVEAQNGGAQSLRRCSHCQVQKTPQWRTGPLGPKTLCNACGVRFKSGRLFPEYRPACSPTFSDDIHSNSHRKVLEMRRKKEIVESDRIQLIPSC
GmGATA42	Glyma12g08131	I	MKDCWFFYNNFNGLSDESLDDVMDMEFLDLPLDFEDVETDAVEEQDWDAQFNKFLEDPPPPLGSFPLQSSEFCGQTQHENVKLGKSFRASLPKTVRPTYGKTIPIQNVSLKGKDLLQFQTNSPISVFESSSSSPSVENSNFELPVIPTKRPRNKRQRLSNISLLFSIPFILTSPTFQKCQRMIFSESDLQTQPAGELLCMVSKKLRKKDIPMLANRIEMKRSSSQESVALRKCLHCEVTKTPQWREGPMGPKTLCNACGVRYRSGRLFAEYRPAASPTFVSSLHSDSHKKVLEIRNRATQVTVR
GmGATA43	Glyma12g29730	I	MSKDIANMKDSWFFDNNFNGLSDEIFDDVINFFDFPLEDVDANGVEEDWDAQLKCLEDPRFDVYSASSAGLCAETQNEKPQLGMKLSASSNGISPIKQLAKAPGPAYGKTIPHQNVTSNGKDLHQFQTYTYSPVSVFESSSSSSVENSNFDRPVIPVKRARSKRQRPSNFSPLFSIPLIVNLPAVRKDQRTAASDSDFGTNVAGNLSNKVKKQRKKDLSLLSDVEMTRSSSPESGPPRKCMHCEVTKTPQWREGPMGPKTLCNACGVRYRSGRLFPEYRPAASPTFVASLHSNCHKKVVEMRSRVIQEPVRCSMLASSNLHGNSVG
GmGATA44	Glyma13g00200	II	MIPAYRHSVSSVMPLDLNEDQNHEFFSPIHHPSSSFSSLSSSYPILFNPPNQDQEARSYDWETTKHLPSHEEEAEKIIPTSGSWGHSVEESEHKVTVWRKEERNENLAEDGSVKWMPSKMRIMRKMLVSNQTDAYTSDNNTTHKFDDHKQQLSSPLGIDDNSSNNYSDKSNNSIVRVCSDCHTTKTPLWRSGPRGPKSLCNACGIRQRKARRAMAAAAAAALGDGAVIVEAEKSVKGKKLQKKKEKKTRIEGAAQMKMKRKLGVGAKASQSRNKFGFEDLTLRLRKNLAMHQVFPQDEKEAAILLMALSYGLVH
GmGATA45	Glyma13g40020	I	MSKDIANMKDSWFFDNNFNGLSDEIFDDVINFFDFPLEDVEANGVEEDWDAQLKCLEDPRVDVYTASSAGLCAKTQNEKPQLGMKFSASGNGISPIKQLGKATGPVYGKTITHQNVTSNGKDLHQFQTYTYSPVSVFESSSSSSVENSNFDRPVIPVKRARSKRQRPSSFSPLFSIPFILNSPAMQNHQRIAAADSDFGTN

			VAGNLSNKLKKQKKKDSSLLSDDVEMMRSSSPESGSPRKCMHCEVTKTPQWREGPVGPKTLCNACGVRYRSGRLFPEYRPAASPTFVASLHSNCHKKVVEMRSRAIQEPVRGSMLASSNLHGNAVG
GmGATA46	Glyma14g10830	II	MTPYSLNPPGPSIQAGQNQLFNISPNNQDCRTFFNIFDPRQTSIEIGGLRENYRQDDKMILHDGSSSNCNSSFNISPETVVMVDPLSSACDRRNLPSEEESKNNDHGSGNKWMSSKMRLMKKMMRPSISPTTDKAINSSPRFQNHQGLESRRYSQRSPRNNNGSSTPRVCSDCNTSTTPLWRTGPKGPKSLCNACGIRQRKARRAMAEAANGLVTPIACEKTRLHNKEKKSRMNHFAQFKNKYKSTTTTTTTTVGSSEGVRKLEYFNNFAISLRSNNSDFEQMFPRDEVAEAALLLMDLSCGFVHL
GmGATA47	Glyma14g22460	I	MESPNSSPIFPQFTFDTNKNNNNPDNFIVEDLLDFSNDDVVITDATFDSITTDSSTVTTVVDSCNSSSFSGSDPNTVPDVGSQNLSDGHFSGDLCVPYDDIAELEWLSNFVEESFSSEDLQQMQLISGMNARNYDVSEAREFHYEPTTRSGPHTPEPTTKSGGLHYEPTRNSPIFNSEVSVPAKARSKRSRGPPCNWASRLLVLSPTTSSSSDSEVTVPAPAEHGPAPAKKAAKAGPRKKDSGSDGNGSGGDGRRCLHCATDKTPQWRTGPMGPKTLCNACGVRFKSGRLVPEYRPAASPTFVLTKHSNSHRKVLELRRQKEMVRAQQHHQQHHQQQQQFLHHHHHNHNHHHHQHHQNMMFDVSNGDDYLIHQPVGPDFRQLI
GmGATA48	Glyma14g24201	I	CFSSPASCVLVPVGVKTTSTKSLSTSINPSLKRPQQQNEPHLQNFVVPGKPRSKRKRLSEPRTNKDPLSIWSHHLNPQIEALCSDPPLLKQAYWLVDSELIMPKPKDNKEQKEEVVIMTKKDEEKVIINRTPQWRVKPLGPKTLCKACGVRYKSGRLLPEYRPSKSPTFVSYLHSNSHKKSWR
GmGATA49	Glyma14g36150	III	MEPSAMYGHSQPLSMPSQIGGGESDDGSGNEHAVDGHHHHIQYETHALEDGAAVVVEDVTSDAVYVSGGGGPVESSQLTLSFRGQVYVFDAVTPDKVQAVLLLLGGCELSSGGSPCVDPGAQHNQRGSMEFPKCSLPHRAASLHRFRQKRKERCFDKKVRYSVRQEVALRMHRNKGQFTSSKKQDGANSYGTDQDSGQDDSQSETSCTHCGISSKSTPMMRRGPSGPRSLCNACGLFWANRGALRDLSKRNQEHSLPPVEQVDEGNDSDCRTATADPAHNNLPAFSEHDNPALVADHKVFQSQKMLK
GmGATA50	Glyma15g05065	II	MDFGKTNEASNSKLVHDFDLNIAYVEEFDHVNAENEFSSPILVNTTQQACKNSIENMNIEDAIAYNRETTIQANSAAKGATSEDTRQVEPKYIASVSFTPARISQYLRRRRHHRGAESKQSTDPDKLCTNFYCKTRKTPMWRKGPLGPKTLCNACGLQYLKMVKGTGSGLPAAVSDEGDASVPAETVERDSNL
GmGATA51	Glyma15g18380	II	MIPTYRYSVSSPMPIDLNEDHTHHLFSTNHQASCSSSSLSYSILFNPDQDQGGSCSDWKSKHLQSDEEAQKIVPSSGLSEKDENKSDLKLRVWKKEDKCENFQGEDNSTKWMPLKMRMMRRLMVSDQTGSDDTEGMISNSQKIKYEEKNSPLSPLGTDDSNYNSSSNHSNITVRVCSDCHTTKTPLWRSGPKGPKSLCNACGIRQRKVRRAIAAAATSNGTNPVEAEKSQVKKGNTLHSKGMKSKTEGAQQMKKNRKLGARYRKRFGAFEDLTVRLSKNFALQQVFPQDEKEAAILLMALSYGLLHGFPTDRYIT
GmGATA52	Glyma16g04670	I	MNMDMCQNVSVSGECQQVQVFAPSCSSSLDDLFSAQNTEVDVELEWLSEFVEDCFSSPPSCVLVPVGVKTTSTKSTSTSINPSLKRPQQQNEPPLQNFAVPGKARSKRKRLSAPRTNKDPLSIWSHHLNPQNEALCSDPPLLKQAYWLADSELIMPKPKDKEEQQEEVVIMAKEDEEKVIINVSKEISFGDSELDEGSNGQQQPMPRRCTHCLAQRTPQWRAGPLGPKTLCNACGVRYKSGRLLPEYRPAKSPTFVSYLHSNSHKKVMEMRMSVYSISSEQ

GmGATA53	Glyma16g24381	I	MGKVLGNIQFYVMATLFINTELTVPAEEEVADLEWLSHFVEDSNFSEYSLPFPATLAEKVKSPEPGNTGFTYKTSVPTKTRSKPTRTSVRVWPLTSSTVTT<br>TTPTTSSPSSSSPSSPLLAYAAADPRVKKHVVIDSAVAARRCNHCGVQKTPQWRIGPLGAKTLCNACGVRFKSGRLLPEYRPACSPTFSIKLHSNHHRKVL<br>EMRRKKEVTPEPDTSSPRSIPNF
GmGATA54	Glyma16g25370	II	MMHHCCGSSQGHVMGTCTCGMYHSETSAYGSMLFSVPNNSEYDMYSSFTPSPSSVDCTLSLGTPSTRLTQDDDHDNDNKRHPHQRRSGVANFCWDLL<br>HSKHNNNNTQSQSKSSSRGSSSNNNNNNDPLLARRCANCDTTSTPLWRNGPRGPKSLCNACGIRFKKEERRASAAAATSTAVPEGEMELARVYGHHHN<br>NSWYAAHSQNQKMMMMGNELRFMDDSEDRDSENNGIPFLSWKLNVPDRTSLVDERW
GmGATA55	Glyma16g26870	I	MDMDVCRNISVSSSECQQELPTLDDLFCHQNTEVDFGMEWLSVFVEDCFSSRPSCLLPPSGGGVQTTSTSTKPSSGTIMPRPQQSHHCPLQNFAVPGKAR<br>SKRKRLSAPRTTKHTLSTWSQHFSSQNDGVSSDPPLLKQAYWLADSELIVPKKKDVEQEEGVVVVVKKEKLGDYYDDDEGDEVNNNNTNNNNNDNVQ<br>HPIPRRCTHCLAQRTPQWRAGPLGPKTLCNACGVRYKSGRLLPEYRPAKSPTFVSYLHSNSHKKVMEMRMGVGVAVLSTDKK
GmGATA56	Glyma16g27171	I	MIGNFIDDIDCGNFFDHIDDLLEFPDDAAAADTSAAAPVPPPANFWSAESDSLPATDTVFSDNSVTDLSAELSVPYEDIMQLEWLSNFVEDSFSGGSMTM<br>KKEEPQCTTTKEDIAPAQFQTASPVSVLESSSFCSGEKAGTEINISVPCGRARSKRPRPATFNPNPVMQLISPASSTGENTQHNAANTSKASSDSENFAESVI<br>KAPKQASGEHKKKKKIKVTFPSGQERNAPSQAIRKCLHCEITKTPQWRAGPMGPKTLCNACGVRYKSGRLFPEYRPAASPTFCAAMHSNSHKKVLEMR<br>NKTGTKSGFATVSAASPELIPNTNSSLTLEYM
GmGATA57	Glyma17g03410	II	MVDPTGKGSEVEVEDSNSNPNAPSSGNSPSSNNEQKKTCADCGTTKTPLWRGGPAGPKSLCNACGIRSRKKKRAILGINKGSTEDGRKGKRTGGGGGIG<br>GIGGGALGREVLLHRSHWKKLGEEEKAAVLLMSLSYGSVYA
GmGATA58	Glyma17g06290	II	MIPAYRHSVSSVMPLDLNEDQNHEFFSPTHHPSSSFSSLSSYPILFNPPNQDQEARSYYWEPTKQYLPSHEEETEKIIPSSGSWDHSVAESEHNKATVWKK<br>AEERNENLESVAAEDGSLKWMPAKMRIMRKMLVSDQTDTYTNSDNNTTHKFDDQKQQLSSPLGTDNSSSNNYSNHSNNTVRVCSDCHTTKTPLWRSG<br>PRGPKSLCNACGIRQRKARRAMAAAAASASGNGTVIVEAKKSVKGRNKLQKKKEKKTRTEGAAQMKKKRKLGVGSAKASQSRNKFGFEDLTLRLRKN<br>LAMHQVFPQDEKEAAILLMALSYGLVH
GmGATA59	Glyma17g15610	IV	MGKQGPCYHCGVTSTPLWRNGPPEKPVLCNACGSRWRTKGTLANYTPLHARAENIDYEDQKVSRVKSISLNKNTEVKLVKRKQNYGNAASGGFVPDY<br>SQGYRKVVDEDTSNRSSSGSAVSNSESCAQFGGPDASDLTGPAQSVVWDAMVPSKKRTCAGRPKPSSVEKLTRDLCTILHEQQSYFSASSEEDLLFESDT<br>PMVSVEIGHGSILIRHPSSIARDEESEASSLSVDNKQCLMNEAYSFSSTIPIYSDRSSMNFSSHGVEKIKNSAGQIMQQEKLERDKSQLEKLQVHGNHDSPL<br>CSIDLNDVVNYEEFMRNLTNEQQQQLLKYLPVVDTAKFPDSLRNMFNSFQFKENLIYFQQLLGEGVFNISLLGAKPEEWKTLERLALSNLSKSKWVEHY<br>NFLKKCENKSGKSIGLGSTAMESSNVTTGKRMREHDSRNQNIPELKTTMRSPKRVIIKPPSCEVKEVVEEGSSFSPKSLFALPHGVGGLHMLDSFNFVGES<br>SEDLLLEVPSNSSFPQAELLHPSLSYGARQVSTTSSSVHSPVTHP

GmGATA60	Glyma17g27110	I	MESPSSSPIFPQFTFDNNNSDHFIVEDLLDFSNDDVVITDATFDSITTDSSTVTTTVHSCNSSSFSGSDPNTVPDIGSRNLSDGHFSDDLCVPYDDIAELEWLSNFVEESFSSEDLHKMQLISGMNAQNNDVSEAREFHYEPTTTRSGSHTPEPTRNSPIFNSEVSVPAKARSKRSRGPPCNWASRLLVLSPTSSSSDNEVVVPSPATAEPCPTPAKKMAKVGPRKKDSSSSDGNGSGGDGRRCLHCATDKTPQWRTGPMGPKTLCNACGVRYKSGRLVPEYRPAASPTFVLTKHSNSHRKVLELRRQKEMVRSQQHHHQHQQQFLQHHHHNHHHYQHHQNMMFDVSNGDDYLIHQYVGPDFRQLI
GmGATA61	Glyma17g34670	II	MTPYSLNPPGPSIQAGQTQLFNISPNNQDCRTIFNIFDPRKTRIEIGGLRDNYHQQDDKMMVLHDGSSSNSNKSSFNNNISPEPVVVMVDPIISSACDQQHNLPYEEESKNIDDHGSGNKWMSSKMRLMKKMMRPSMSPTTDKAINSGLESSSSRYSQRSLCNNNASSTTRVCSDCNTSTTPLWRSGPKGPKSLCNACGIRQRKARRAMTKATSGLITPITCAKTRVHNKEKKSRANHFAQFKNKYKSTTTTSAGSSEGVRKLEYLKDFAISLRSNNSDFEQGFPRDEVAEAALLLMDLSCGFVHL
GmGATA62	Glyma19g28650	I	MNMDMCQNVSVSGECQQVQVFAPSCSSSLDDLFSAQNTEVDVELEWLSEFVEDCFSSPPSCVLVPIGVKTTSTSTNLSSGTLKRPQQQNESPLQNFAVPGKARSKRKRLSAPRTNKDPLNIWSHHLNPQNESLCSDPPLLKQAYWLADSELIMPKPKDEEQEEVVTKEDEKVINVMSKESFGDSELEEGSNGQQPMPTRRCSHCLAQRTPQWRAGPLGPKTLCNACGVRYKSGRLLPEYRPAKSPTFVSYLHSNSHKKVMEMRMAVFSTISSEQ
GmGATA63	Glyma19g41780	II	MMDLKEWSSSSEELNVNRKCCADCKTTKTPLWRGGPAGPKTLCNACGIRYRKRRACWRKGELKKQKQKQKQRWKMLGEEEQAAVCLMALSCGFVFA
GmGATA64	Glyma20g32050	I	MVGPNFMDEIDCGSFFDHIDDLLDFPVEDVDGGAATLPSVAAAGNCNSLASIWPAESDSFPTSDSVFSGNTASDLSAELSVPYEDIVQLEWLSNFVEDSFCGGSLTMNKVEEPSCTTKEDSVNTQFHTSSPVSVLESSSSCSGGKTFPLSSPEIYIPVPCGRTRSKRPRPATFNPRPAMNLISPASSFVGENMQPNVISSKSSSDSENFAESQLVPKMPKQASEEPKKKKKVKLPLPLVPADNNQNASQPVRKCMHCEITKTPQWRAGPMGPKTLCNACGVRYKSGRLFPEYRPAASPTFCPSVHSNSHKKVLEMRCRGIDKSGFAINSAASPELIPNTNSSLPLEYM

Table S2-3 Sequence and information of GATA proteins in *Solanum lycopersicum*

Gene	Gene id	group	Sequences
SlGATA1	Solyc01g060490.2.1	II	MDLTDKVIGSEATEMTTPEVMISSEMEVKAAEMMISPEIKAKAPEVISSEIQMKTPEVISSEIQMKNCVNCGAMKTPLWRSGPAGPKSLCNACGIRSRKKKRDLLGLNKDEKKTKKSSANSASSSNVDKKKKKKICAVKNADAIPIHWTDLDDVEQAAFLLMCLSCSSVCA*
SlGATA2	Solyc01g090760.2.1	I	MDVYGLHSAPDLFRIDDLLDFSNDEIFSINNNSNNTDSNHHHQPHSHNSSAAGPANYYDALLPNSSDDFTDNLCVPSDDVAELEWLSNFVEDSFSNFPANSVTGTMNITSNTASFHGRSRSKRSRSTSSWTSSLQNSNATTSVKNKESSVYTRERSSSMDEDVPRRCTHCASEKTPQWRTGPLGPKTLCNACGVRYKSGRLVPEYRPAASPTFVLTQHSNSHRKVMELRRQKEMIHQPQQQQQMPPSTEEGMYGHHFRVC*

SlGATA3	Solyc01g100220.2.1	II	MDPNQKDESSGSEATGISKSCSDCKTTKTPLWRSGPSGPKSLCNACGIKYRKKKSSPIGLTKGATKKKEKPLSNSGSTEEVEYCKKGKMGNGKKDGKLS<br>KVLRVKLMMLGKEVVILQRQRSAMKKPRKLDEVERAAVLLMALSCGSVFG*
SlGATA4	Solyc01g106030.2.1	III	MAEANRRANMYGRETMNAALHQDRHQQTQIDDDDDDDVVAAVGGGGSGGGGIESMDNPTPHIRYDQHHHSHSHALHNGGAGGSMEMNGVEGVSH<br>NALYGPPSEIVPTAGSGASDQLTLSFQGEVYVFDAVSPEKVQAVLLLLGGYEVPPGIPAVNVVPQSQRASGDFPGRLNQPERAASLNRFREKRKERCFDK<br>KIRYTVRKEVAMRMQRKKGQFTSAKSIPDEVGSSADWNEGSGQEEQETSCRHCNISSKSTPMMRRGPAGPRSLCNACGLKWANKGILRDLSKVPAPGT<br>QDQTAKPGEQSHGEPNGSDDMAAIITPDDNNPVG*
SlGATA5	Solyc01g106040.2.1	III	MEAADNSRKMVGGQYGVVAFQQEYVNVPIRVDRYGGGGFEAHDISVGGGYEEAMSSGEELQVCSMPLVAGPVDSGRVMAVSSRTSELTISFEGQVYV<br>FPAVTPEKVQAVMLLLGGCDVPSYVPNSNSVALPSTKSVENDVPTRQNISPRMASLIRFREKRKDRCFEKTIRYACRKEVAQRMHRKNGQFASLKEGGK<br>SSADNIDSGDSAAQSEPTLRRCHHCGTNESETPAMRRGPSGPRTLCNACGLMWANKGMFRDITKGGSHVPFDKNEPGTPDIKLSTFAPENSYLKQHQEG<br>SSGETKLTEENPSVTTAELDMQEAAENFADSSPFRIRSSSVNIDDEDNLDELANASGTEFEIPADFDEQIGIDSHMSVHWPVT*
SlGATA6	Solyc01g110310.2.1	I	MELIEARALKSSFLSDMAMKNTQQVFLDDIWCVTGINNGASEDFSVDDLLDFSDKDFKDPELHEDDEKTSFSGSSQKRNSQDSTFSGMESFGSLAGELPIP<br>VDDMENLEWLSQFVDDTPSEFSLLCPTESFKDKTGGFTESRSEPVVRPVVKKTRVPCFPLPFPVKPRSKRSRQAGRTWSFPSSAVSGDSSSPTSSSYGSSPF<br>PSGFFTNPVYDGDLFCSVEKPPLKKPKKNPSVETGSGRRCTHCQVQKTPQWRAGPLGPKTLCNACGVRYKSGRLFPEYRPACSPTFSLEVHSNSHRKVLE<br>MRRKKETGEGIDSGLASMISTC*
SlGATA7	Solyc02g062380.1.1	I	MKFYPHVVYMVPVCYVFGQVTQQIFKQKMDYSGNCQSFVSGDDLFVDELLDLSNGFSEDEENENENNNSSSSQQIKECDKETVIIPSGKQDFGSLLGCEI<br>SLPGADLDNLEWLSHFVEDSFSEYSLTYSAGNLPGKSLKLHSNVEIPVREKPCFTAPLGAEREKNKPPSMSFSSSSSTTANSFWGELSVENKPAARKPKKK<br>MEKRIGIGAKQCSHCGVQKTPLWRTGPLGEKTLCNACGVRFKSGRLLPEYRPASSPTFSTGLHSNSHRKVLEMRQKKETEPGPPVQSF*
SlGATA8	Solyc02g062760.2.1	II	MNSCGNAYCHGGPCTCNLYYNHENNNASSAYSMLYHEYHQKDENHSENMYSFASSPSSSSSSVDCTLSLGTPSTRLSNDERRLHDSSHEPQRRSSSYMS<br>KCWNLLQNKNNHTSSTHHHKSGRGSSNNNVNSSSSNNIADPLVARRCANCDTTSTPLWRNGPRGPKSLCNACGIRFKKEERRASAAAAANGIGIGSESST<br>QHTINGSWVHHSQGQKMPCYTSAYGNHEFRFIEDHDDRDSDNAISFWPFLASQHC*
SlGATA9	Solyc02g084590.2.1	I	MTQNQTFGDDFSAAGAGQNGVSGDDFFVDDLLDFSNGFVEGEGDEEEEEGKNQGGEGISVQKPCSVSIAVSPLKKTEIDDKGKVTISVNEDFASLPVSEIS<br>VPTDDLDSLEWLSHFVEESFSGYSLAYPAGKLPVEKKTGDGEIPVEEKKPCFATPVQTKARTKRGRSSVRVWPVCSGSLTESSSSSTSSSSTTTMSSSPPTG<br>SWFLYPTPVHSAESPGKPLAKKLKKKPASHGGNGPQQPRRCSHCGVQKTPQWRAGPMGAKTLCNACGVRFKSGRLLPEYRPACSPTFSTELHSNNHRK<br>VLEMRRKKESEETGLTQPVQSF*

SlGATA10	Solyc02g085190.1.1	II	MMHRCSGSMVGPCSCGLFHNQSGSSGTTTSSAFSMLFSHEYSENMYSSSSSNVDCTLSLGTPSTRLINEDNHEKRSSVYSKHERLRSSSNCISNFWDILHPKQHVTTPQYKSTRGANNNSTTTDPLVARRCANCDTTSTPLWRNGPRGPKSLCNACGIRFKKEERRASAAAATANGGGGAETTTHQHWVHHHHHSQPTKMACFSSASYGNEFRFIEDNEHHRDSDAAATAIPFFPWRLNVADRPSLVHDFTR*
SlGATA11	Solyc03g033660.2.1	I	MLYRTAPHYSSSPLQMNSLEKALKTSYFRPETAMKMTHNQPSIDDFFVDNLLDLSNGFAEDEIEQLNEHPNGFNTQNLCSVSPQKKMEDENGDFGCELSYPENGLDNLEWLSQFVEEDSHSGYSLIGKLPVKKNKSVTENPVQVNSCFTVPVQTKPRTKRRRIGGRVWSFTGSSTSSASSSTITTTAESIVRFPASVSNRRKMKTEKPVQPRRCSHCGVHKTPQWRTGPMGAKTLCNACGVRFKSGRLLPEYRPACSPTFSSERHSNNHRKVLEMRQKKEERTGGDRFAPPVHSF*
SlGATA12	Solyc03g120890.2.1	I	MEASDLFVSGFFSHAGDEQIPNNINNNCNNFSVDDLLVIPKDDEVMADAFFNSITGNSADSSNVTVVDSCNSSVSGGDGQFNGNLSGRSFTDAPFPNSELCVPFDDLAELEWLSNFVEESFSSDDVQNLQFIPVANINSSSTVTTDSSSSATTFSTGPNSPPAFPADTSVPGKARSKRSRAAPCDWSSRLQLLLSPATSSSESNNISPPSVNNTTFATAKATKAPSKKRESVETPGRKCLHCASDKTPQWRTGPLGPKTLCNACGVRYKSGRLVPEYRPASSPTFISARHSNSHRKVLELRRQKDLQRHQAHHHQHQLLSQPTIFGVSNGGDEFLLHHHQNCGPNFRHLI*
SlGATA13	Solyc04g015360.2.1	I	MGSDFVDEIDCSSFFDHMEDLIEFSPENVCGGVDAVDCNDFPSIWNDPLPDSDPFFSGSYSNSASDFSAELSVPLEDIVQLEWLSTFVEDSFSCGGLTLGKEHCSVKKESSDSKFLTSSPVSVLESSSSSSSSTSSSGPGKTLPPLSPCPRGPQRARTKRPRPTTFNPRSVNHLMFPTSVSPQQFVAPGVNSLDSENFAESPMKKKKKIKLSIPLAPIETNQDNYQPAPQAVRKCLHCEITKTPQWRAGPMGPKTLCNACGVRYKKGRLYPEYRPAASPTFVPSLHSNSHKKVLEMRSNVFPEEETHYKQAKPRRARPLITVQAENNTTASTTPTE*
SlGATA14	Solyc04g076530.2.1	III	MIPQIWLQKMNRQMQAMNVDIHFSGDGEEVPAGNEKAVGNFPVGYEGPVDSVKVTMPHQTVCSASDVVALQNRETDFDQLTISFRGKIYVFDGVTTQKVHSLFKLLGGYEYSPGTQALGLLSANQKDYVDHPVHCADPKRLESLIRFYEKRKKRCYEKKIRYGIRQEVAFRMKRKNGLFARKGSNGPKQENIPSEETRCIHCGTSSKATPMMRRGPDGQRNLCNACGLTWANKGIMRTLYKASYDNTELELEIATKPTMKLLNVPPLIKFLHQSASDLDALERKPYCINISSSHPTERLYIHTTDKSIMMAENPRPLQARPFEDHAPLQSLRMVEEEEEEEEEGDEDGAYEDDGGEETMDEAEDVSMKVLNHHQYGGQSQQLCGGGVVEVSRTSELTLAFEGEVYVFPAVTPDKVQAVLLLLEGCDIPTAVPTVELPFDNKVEDLPKQANLSKRFASLVRFREKRKERCFDKKIRYSVRKEVAQRMHRKNGQFASLKSSSGSSSCDSAKSSLEGDGTQHSLTIPGKCHHCGVSENCTPAMRRGPDGPRTLCNACGLKWANKGTLRDLSKAGRTISVELNELGTPNNPVKAFAEGSLDHCADVEKNLFSSGSNLANDIPAGIISSSNNLVEKL*
SlGATA15	Solyc05g053500.2.1	I	METMYSKTCFMVDDDILNFSLDDDEEKYQTSSSSFESSNTLGFRQDDHNPSFPDYVEEELEWLSNKDAFPAVEFDLFSDHVIFDHHSPNSVLENNNNNCNVNLKDNAFTSHASSLLQVPMNHPVGTRSKRRRRIALQCDNSCVWGNQVKFNNTSTKQGLTLLKISMAKAKRGTSIGRTCQHCGVDKTPQWRAGPTGPKTLCNACGVRYKSGRLFPEYRPANSPTFSVELHSNSHRKVLEMRKQRI*
SlGATA16	Solyc05g054400.2.1	II	MVDLSDKQGLGSEEMSSGVTSPETSQSNVKTCADCGTTKTPLWRGGPAGPKSLCNACGIKSRKKRRAFLGLNNEEKKSKKSVVVGHKNIEVQHHLNQSCSSSSNSDDSKSSNFVKNIVSSSLKKKLLPFGKEEVVMQRPRSRSTQKRKLGEVEQAAFLLMALSCGSFYTHGRMKIDKERKNEKCCTLGSKSSTLWVG*

SlGATA17	Solyc05g056120.2.1	I	MGSNVVDEIDCGSFFDHIDDLIDFPLESENVGLSSTDCKDFPSIWNDPLPDSDSLFSGSHRNSASDLSAELSVPYEDIVQLEWLSTFVEDSFSGGGLTLGKENIPVEKEPSSQGKFQTSSPVSVLESSSSSSSSSFSSSGEKTLPLSPCHRGPQRARTKRPRPTTFNPFPVFVAPVVPTESENFAESPMKKILKPAEPEQKKKKKIKFSIPLAPVETNQNPVAQQAVRKCQHCEITKTPQWRAGPMGPKTLCNACGVRYKSGRLFPEYRPAASPTFVPALHSNSHKKVLEMRTTIVPDNDIIARTSSPAIATQLEFNPSNVSVEEEHK*
SlGATA18	Solyc06g060940.1.1	IV	MGKQGPCYHCGVTSTPLWRNGPPEKPILCNACGSRWRTKGTLANYTPLHARPESDDLDDYRVCRVKNMSLKNKEAKVLKQKQNHDNTVVRTPPDYYQGFLKSLDEDTSNRSSSGSAISNTESSAQFGSTEASDLTGPAQSNKWEAMVPSRKRTCISRSKHSSVEKLTKDLCTILHEQQSSYFSGSSEEDLLFESDKPMVSVEIGHGSVLIRHPNSIGREEESEGSSLSVNNKRHYVNEAYSRLSAPPVNINRGVNLPNLGTEITKKPNSQGMEQHPIKRDKDHLEKLHILGHHTSPLCHIDLKDVLNYEEFVTHFSSDEQKQLLKYLAPVDSFAPPDSLKSMFHSSHFEENLSSFQKLLAEGVFDSSLPGVKLEDSRTLKRLILCYLTKSKWVEKYNLFKEPKCTSSANGNEVTGRPNAIGTGHSGNVKRPLEGHHPKYAGAKKAMKSPKRVVMKSSYEQKELVDNNNSCFSPKCLFPLPSKSSPVLDSFHVTNDQDVLLDVPSNSSFAQAELLLPTSNFAAQASTGSSSVYPHLVRP*
SlGATA19	Solyc06g075140.2.1	I	MVEQNYMDGISMGHIENEDFESILNGLDFSIQNLEADRLDEDWDATVYGELLGPIPSETLMSLPPLELTNVDNVFPEAQGNVIFQTGSPISVLENTRSCSGGRSAISFNFGSKGRRSKRARSSTLNPWLKMAPMPCTTSAAKKNSDSKIGKVNKRKLSSAMASPLFKRCTHCEVTKTPQWREGPLGPKTLCNACGVRYRSGRLLPEYRPAASPTFIPSLHSNSHKKVVEMRRKTVESSPEFDSQNFVPLGSYLLDEYF*
SlGATA20	Solyc07g038160.2.1	II	MTPPDHITPNFPFGLNNSNNNSLVTPNYHFFFNSTTNQTASFHHQHTQYYMQHEQLEVDNDGGSSYDLGKKNEVGSGLKLSLWKREDKLLSSEIKKLDQEKKKNSTNSACIKLKLGDQKQKPIQTDYCSNNIPIRVCTDCNTTKTPLWRSGPKGPKSLCNACGIRQRKARRAMAAAAAEGKTDQKVQQHKQNITTKVTSNNDVKPLKKRCKFGPSSSSTNNAPKKLGFEDFLINLSNKLAFQQIFPQDEMEAAILLMALSSGLVHG*
SlGATA21	Solyc08g007190.2.1	IV	MGKEGPCYHCGVTSTPLWRNGPPEKPILCNACGSRWRTKGTLANYTPLHARAEPCDFEEHRVSRFKNISMKNKEAKILKRKQSHHDAEVGTPDYSLGFRKVLDEDTSNRSSSGSAISNSESCAQFGSAEASDLTGPAQSNIWDSTVPSRKRTCFNRPKPSSVEKLTKDLYTILHEQQSSYLSASSEEELLFESDKPMVSVEIGHGSVLMRYPSTIGREEESEASSLSVDNKHRSVSDAYSRLTTPPVNISKGVNSPNMGTERIKKPTGPAIEQDQIKRNKDHLEKLQILGHHNSPLRYLDLKDVLNYEEFTTHLSSDEQQQLLKYLPPVDSFAPPDSLRSVFESSQFEENLCSFQKLLAEGVFDNSFPGVTLEDCRNLKRFILCYLTKSKWVQQYNLLKDTKCKNSSSGSEVAGEPNAVGTCHSANVKKPREGQYPKCSGAKTTMKSPKRVVMKSIYEQKELVDNDGSCFSPRSSFALPSENSSLVLDSLRSANENSHQDLLLDVPSNSYCPQAELLLPTSSFTTQASTSSSSMYPPHFIRP*
SlGATA22	Solyc08g066510.2.1	I	METPDFFQTGYYNSSEKQLISDVKNGEHFVVDDLLDLPNDEGMATDDTLDLTVIGNSTDCSVVHNSCNSSLSGSNHHPQSLGYRDFPQGHLSTEFALPYEDMAELEWLSNFVEESFSSNEMHKMQMVQAMRNRTDSEIHQFIPDPNRASATSNTIFKPEMPVPAKARSKRSRMAPGNWASRLLVVSPNTTNPDSSMDTISVQDMSSSSESGMIIPSSGKKTVKCSSAPKKKENNIHHVPSNNTGSNSEGRKCLHCATDKTPQWRTGPLGPKTLCNACGVRYKSGRLVPEYRPAASPTFMLTKHSNSHRKVLEIRRQKEVTQVEHQHQHQFLPHNMMFDVSNADDYLIHQHMGPDFRQLI*

SlGATA23	Solyc08g077960.2.1	IV	MGKQGPCYHCGVTSTPLWRNGPPEKPILCNACGSRWRTKGTLINYTPLHARAEPDDLEDYRVSRFKNFTVKNKEVKMLKRKKSHDNPEIGILPEYHQGF<br>HRKALDEDTSNRSSSGSAVSNTESYGQFGSAEASDLTGPAQSNIWDATVPSRKRTCVNRPKQSSVEKLTKDLYTILHEQQSSYFSGSSEEDLLFESDKPMV<br>SVEIGHGSVLIRHPSSIGREEESEASSLSVDNKHHSSNEAYSQLTTPPVNISKGVNSSNLVIQRTQKPTVQGVEHEQFKRSKDHLEKLQILGHHNSPLCHIDL<br>KDVLNYEEFTRHLSSDEQQQLLKYLPPVDSFSPPESLRSMFESSQFEENLSSFQKLLAEGVFDNSLSGVTVEECRNLKRFMLCYLMKSKWVEQVNLLKD<br>MKCKNSSSSSEVAGGRNVFGTGHSVNMKRPRDGQHPKNLGVNTTMKSPKRVAMKNSYEQQKEIMENDGSCFSPKSLFALPSENSFRFTNESSDQDLLL<br>DVPSNSSFPQAELLLPTASFAAQASTSSSSIYPHLIHP*
SlGATA24	Solyc09g075610.2.1	II	MKSCSYCHTTTTPLWRSGPSGPKSLCNACGIKYNKKRRQILGVEKKRRIEKKSIEIGKLMGFGGKLREEEQAAILLMALSCGSVGKLLC*
SlGATA25	Solyc09g091250.2.1	I	MKMEDLDPTACFMVDDDLLNFSLEDETVEEDDEKSTITSKDPLSYSSSSSTNPLVSLLPHPECVEEELEWLSNKDAFPAIEFGILSENPGMVFDHHSPVSVL<br>ENSSSTSHSSGNGVVSGNAYTSCCVNLKVPVNYPVRARSKRRRRRRRGGFADMPSEHCLPVTQPSFKNVKQREPLLSLPMNSAKSAASIGRRCQHCGAD<br>KTPQWRAGPLGPKTLCNACGVRYKSGRLLPEYRPANSPTFSAAAHSNSHRKVLEMRKHKIGVGGMLIHEACGYRLLGMYLLSSFNSCRRWCSLEMEVL<br>KFTVF*
SlGATA26	Solyc10g018560.1.1	I	MDVYGRLTPEVFRIDDFLDFSNEEDIFSSSKTAIDFDLNHHYQPPPTDSIADTGCYYHAPPNSVDFTDKLCVPSDDVAELEWLSNFVEDSSNNFPSNNLTQ<br>TMYHLNNTNTILHSKSRSKRSRNSNSTSWNTSSLQRHKSANQKNSNQDENSGDYNSNKLSNNSKIITSRKCTHCASEKTPQWRTGPLGPKTLCNACGVR<br>YKSGRLVPEYRPAASPTFVLTQHSNSHRKVMELRRQKEIIDQQQQHGMYGHHYPVC*
SlGATA27	Solyc10g047640.1.1	III	MTAAVQREGQQQTHFADDGDDDDVDVAGGGGGGGMESIEDSSHHIGYDHNHHGLHNGTDGTIATTAAALNGVEGVPHNSLYVPGSEMVGGGSSDQL<br>TLSFRGEVFVYDAVSPEKVQAVLLLLGGYEVPAGIPTVNMASQSHRASSEGPGRLNQPQRAASLSRFREKRKERCFDKKIRYTVRKEVALRMQRKKGQF<br>TSSKTVSDEAASSSAEGNAGSSQEEQETLCRHCGTSSKSTPMMRRGPAGPRSLCNACGLTWANKGILRDLSKVSTTGAQELSVKSSEQNGEADGSDVMA<br>AAGIITSDDENMVLPSE*
SlGATA28	Solyc11g069510.1.1	I	MDEIPTGPIVDDDFDDILNFLDMPMESLEGDVLGGVEWDVSESKGFGPIPTEALMDFLPLPQSNIGNRRVNAVANSHPPIKFTEVQGTGTFQTQSPVSVLE<br>GSNSCSGGKSVPIKHDPVIPVRPRSKRARPSAVNPWVLMAPISSTRVASKKISDARKTKERRRRLSLLSGAKEPMKNYVQQISDAAPPVSDVSKKKITSTQ<br>QSSFFKKCTHCEVTKTPQWREGPLGPKTLCNACGVRYRSGRLFPEYRPAASPTFVPSVHSNSHRKVVEMRKKTLYGGAGEVEEPPKVIMGRSSEALPEPT<br>IAADPAMSPAPEFVPMSSYLFDVY*
SlGATA29	Solyc12g008830.1.1	II	MSLVSPYNNNYQFASSSTNSSCQNFFNISTTTNIQDQSGYDYQFHQPQHHHEVDNFASRSSGSHDHVDKKNKGLKLTLWKKGGQKVKNLKVEDQKQQI<br>IETDYSSNSSSNNNIIPIRVCSDCNTTKTPLWRSGPKGPKSLCNACGIRQRKARRAAAAAAAASTTPNNGTNFTSTETTTTTTMKIKVQQQKHKITKVNAN<br>HVVPFKKRCKFLSSTTTPAPEPGLVPTPAPRVGSSSSSSFYNNNNNDVQQKKKICFEDFFINLSNNLAIHRVFPQDEKEAAILLMALSSDLVHG*

SlGATA30	Solyc12g099370.1.1	II	MVDTSDKVQETGEMIGQNQTPERVTSETNQKTCVDCGTTKTPLWRGGPAGPKSLCNACGIRSRKKRRALLGLNKDDKKSKKSSSKSVVNHHQQNQSSN<br>SSSSTSSSGESMSNVIVKNECIPYKKRLLHFDREVGLQRPRSNSTHRRKLGEEEQAAFLLMALSCGSVYA*

Table S2-4 Sequence and information of GATA proteins in *Brachypodium distachyon*

Gene	Gene id	group	Sequences
BdGATA1	Bradi1g03020	C	MDSPVEKGSGSLDPDDRTASGEPKACTDCNTTKTPLWRGGPCGPKSLCNACGIRYRKKRREALGLDGPKRRETAACAHTAGEGAEQPPKKKTKREREEV<br>TVELRMVGFGKAAVLKQRRRMRRRRRLGEEEKAAILLMALSSGVIYA*
BdGATA2	Bradi1g09550	B	MASETAADGHDHPPAVAPGNDENAAAAEALLSTASEQLTLVYQGEVYVFDPVPPQKVQAVLLVLGGCEVPPGLVSMAVPTAYGEKSTTVAAKRVASLM<br>RFREKRKERCFDKKIRYGVRKEVAQKMKRRKGQFAGRADLGDGACSSAVCSSQANGEDDHFLETHCRNCGISSRLTPAMRRGPAGPRSLCNACGLMWA<br>NKGTLRSPLNAPKMAVQHPPNLSKMDVLNMDYDKTILCAENDQTTIKMDSGMSPEQVQRLEPPPTDRRRQHDSFPTKDNFMTVDKHLG*
BdGATA3	Bradi1g12330	B	MSDAAAAQEADADMRDVDAAAPAPSEDDAGEEEEGDKEGEEELALAEEPPVSLVPALPANPNQLTLLFQGEVYVFESVTPDKVQAVLLLLGTGEIPPDLA<br>GMVLRSQHENRGYDDLLQRTDIPAKRVASLIRFREKRKGRNFDKKIRYAVRKEVALRMQRRKGQFAGRANLEGESPSPGCDPASQGSGQDFLSRESKCQN<br>CGTSEKMTPAMRRGPAGPRTLCNACGLMWANKGTLRSCLKAKGEAPMVAIEQIGIDIKALTSQNNDNVAPGNVEAIGDSTHANVAVIEGVPKAQSE*
BdGATA4	Bradi1g33980	B	MAQHDGKPYQPRRGPERPPSAPQPADDAALGASAVDHLAAAAAEAEAMNRFDEEHEQQEMEEEDEEEEEVEEEEMEEDDDDEAEGDGQLDGGGNGESV<br>PMDAAAAAAAAAAAGVPLDPHGAMVAANAANASTNQLTLSFQGEVYVFDSVSPDKVQAVLLLLGGRELNPGLGAGSSSSTPYSKRLNFPHRVASLMRF<br>REKRKERNFDKKIRYTVRKEVALRMQRNRGQFTSSKPKPDEATSELATPDGSPNWGSVEGRPPSAAECHHCGINAKNTPMMRRGPDGPRTLCNACGLMW<br>ANKGMLRDLAKSTPTPLQVMVSAPNDSNGNAVVAPAAEQENPTAVVTNGHESST*
BdGATA5	Bradi1g37480	C	MSTIYMSQLSAAFPLMEEDHHQDHHQGHFQNFTLPKDPPILFPFVINNSSPSDNSLSYGSGHHLRQQHHAMLEAPQHMIGGSSSVFLAPFPTVESIRDDMIER<br>SSSYDPYDIEKLQATNGSLKARKWTAPAPAAKMRITRKTSDPAGTVKKPRKRAQAYEDHHMNQGQALGVIRTCSDCNTTKTPLWRSGPCGPKSLCNACGI<br>RQRKARRAMMAPGAAPLTTGSGIVGGKGTGDAHPKAKKEKRAADVDRSLPFKKRCKVVIQDHTATNGAAPVEANAAEPAAVSVSTAAAAPVKEGLVNT<br>IGVNWSNSPTAPGTACSFLPSSVPALDEITDAAMLLMTLSCELVRS*
BdGATA6	Bradi1g75420	A	MAGGGHVDKDAAALAAQDLPGLNVFFDQTGLETAAGGEGAGDEEEELEWLSNKDAFPSVETMAVEAPPELEALAATRPAVVGPRTKGVRRRRRVTAPW<br>NVLTPAVLPPPARAAGPRRKCTHCASEETPQWRLGPDGPRTLCNACGVRFKTGRLVPEYRPAKSPTFSPLLHSNSHRRVLEMRRRNQDDDGETPRATAAA<br>RRAERAASRLSAKAAADAPAQTA*
BdGATA7	Bradi1g78540	A	MAQEWEAAMGMELGMGTTPHYAASPAAAAAATMAAPFGHGSAYSHSLPHHYHFYGGSGAEVADPMRVDEMLDLSSHLGAHDFFPGGSNGAAQGEQA<br>PPPAAPSSSDHHGHGHHHSSSNSFNLSFADEFFVPVPREEAAELEWLSNFVDDSYPDTPNYPPAVQAAARNGARQEMLHNNNNPASTALPGRGARSKRSR

			AASAAAAAWHALVPRHQEHQRPSPSSSSSSSDQQQLVSSSKPARPKAELGSEEQGGVRRCTHCASEKTPQWRTGPLGPKTLCNACGVRYKSGRLVPEYRP<br>AASPTFVLTQHSNSHRKVMELRRQNEQLVHIRGGAAAGSPSSGSAASGEHMFRDYGVC*
BdGATA8	Bradi2g12590	C	MGSSDRKVDGIGVVEEGRRSCVECRTTTTPMWRGGPTGRRSLCNACGIRYRKKKRQDLSLDQKEPPPRQQQHNGEEAITAEVKDSTSNSNSSSGSSNLQV<br>VQERKLLMGVEEAALLLMTLSSPPPSTLLHG*
BdGATA9	Bradi2g14890	A	MLQQYCGGAMAAASTNTFSLFFPPVPAKAAAAGQQWPPTEQAASYEDLSSTVTSPSSPSSDDSSAGSLLVDCTLSLGTPSSRAHRAAEPVSAAAESCYHYY<br>QQQQQSMPAAGTGIGIGMGGVWHEQQQLEEERRCCANCGTSSTPLWRNGPRGPKSLCNACGIRFKKEERRAAETNGAGAGGCGLLSPSHGAQRMIRAPR<br>AAPEAPFLEWRLNAVPTSPAVWPERASLYQQ*
BdGATA10	Bradi2g19480	A	MEAAAAECAAGGAREKKDAGEIFLVDDLLDLPCDEEEGDEAAAAAEGLDGDCGGGGCGEAGNASGDSSTVTAVDSGNNSLSGLADGDFSGGLCEPYDQ<br>LAELEWVSNYMGDDNLPTEDLRKLQLISGFPSSQLPAAAPRAPAPKLAACAGAGAGAGGALHSEPALVPVPGKARSKRSRVAPCNWSSRLLVLPPAPASP<br>PSPASAVISPSESGTAFPPFPAKKPAKAAKKKDPLPAAAAPMTAAAMAAAEGRRCLHCETDKTPQWRTGPLGPKTLCNACGVRYKSGRLVPEYRPAASPT<br>FEASRHSNSHRKVVELRRQKEPPQQQQHLHHHGHQPQPHPGLQVHVPSPLLFDGPLIAGADEFLLRNRIGHHHSYR*
BdGATA11	Bradi2g32792	A	MCQHCFTRKTRQWRLGTREKSPLCNACGIRLIKNGELHPEYHLAASETFVGAIHSNIHRRVLELHSENQGTDGSSSSTGETAA*
BdGATA12	Bradi2g35057	C	MDSPHHKAIGVAAAAAAEGGRMCCVECRTTTTPMWRSGPTGPRSLCNACGIRYRKKRRQELGLDNKQLQSQPQQQQEQQQHQQQEQQQQQQEQQQHQ<br>QQEDHSEPTSAVKDSSSSPSKTSKLQVVKKRRVSMGVEEAASLLMALSSSSTPTLHG*
BdGATA13	Bradi2g45750	C	MLHEAAPCTCGLLYGSCGGGCSMLFATAAPGDYHHHYYSNSKQCGDDSGFNGSSYGGGSVDCTLSLGTPSTRRAAEAAAGLPWETAAVSSCNGNARQE<br>TIATAPRTDHQSANNNASAARRCANCDTTSTPLWRNGPRGPKSLCNACGIRYKKEERRAAAAAVAPTAAGLASDSGMEYAYGYARQQQQHQQQQWGC<br>YGPAVAKAAASYGMFGDAAVAEVDGPCLPWGLGVMPSSPAFGSVREMPSLFQYY*
BdGATA14	Bradi2g49620	A	MEATAPPEYDGYYGRDKKAVVACAGDHFVVDDLLALPACDDDDEEGDGGEAFLAVDTQQLPPAKEEGGGGFGNASGDSSTVTALESCTNSFSGLADGD<br>FSGGLCEPYDQLADLEWLSNYMGEGEEAFASEDLQKLQLISGIPSGGFSSAGARPPAPAAQAAAAAQQPTMFLPEAPVPVPAKARSKRSRAAAGNWSSRL<br>LVLPPAPASPPSPASMAISPAESGVSGAAQAFHVKKPSSKPAKKKDAPQALAPTSAPGTPTGVSAAASEGRRCLHCETDKTPQWRTGPMGPKTLCNACGVR<br>FKSGRLVPEYRPAASPTFVTSKHSNSHRKVLELRRQRDMHHHHHGGQQQQQQPPQHVVTGSLMHMQSPLLFDGPSAPPIVAAGDDFLLHHRSATDYRQF<br>M*
BdGATA15	Bradi2g62660	C	MEPDMVVVADPDECTASGEPKACTDCNTTKTPLWRGGPTGPKSLCNACGIRYRKRRRQAMGLDPEVKKKPKKEDAAAANTNTKAASAGAADQEGKDK<br>DKEKKEPRTHTVELHMVGFAKDVVLKQRRRMRRRKPSCQGEEERAAILLMALSSGVIYA*
BdGATA16	Bradi3g03800	B	MSGHHDATKPYQPRRGPERPPPPPQPAEGDADAGAVPEAEAEPLAMEQYEPEPLQYEPHPEHEEEEMEEGDEEEEDEEEGEGGHGHEYAYDYGTGEAVP<br>MDADAAPHAQMQGAMLPMPANAEAGGGGPHAASNTLTLSFNGEVFVFESVSPDKVQAVLLLLGGRELAPGLGGGPSSSASYSKRMNYHPHRMASLMRF

			RQKRSERNFDKKIRYSVRKEVAHRMQRHKGQFTSAKAKAEDGASPVTTSDGLTNWGAVEGRPPSAAICKHCGKSSDMTPMMRKGPDGPRTLCNACGLS<br>WANKGHMRDISKCSTAPLQVVPPANSDAPNGTTEAAPVDKQQHLAIEAAPVVQPAPAANGHDS*
BdGATA17	Bradi3g08240	C	MSTIYMSQLSTLPLMEGDQDQGHFQAFHLPKDPPILFPFMIDNPVEHQGQGYGDQHLTQQFFGESSQQFNDHMMMAGGSDVFGTPSPFQPTIQSIGSDMIQ<br>RSSYNNPYDIKSKQAVSGLTSEWASTTPPVKMRIVRKAATSDPEGGAARKPRRRAQANQAEESQQQQHAMGVIRVCSDCNTTKTPLWRSGPCGPKSLCN<br>ACGIRQRKARRAMAAAAVTAANGRVTTAASNGGAVAMVLGTQASNAYQAAKPAKKEKRAADVDRSLPFKKRCKMVDHAAITITSESVASIAAPKEDQH<br>HAGEEGVAVAERPSKAGPPAAHFHGFPRDEITDAAMLLMTLSCGLVRG*
BdGATA18	Bradi3g28003	A	MVLVDGLRGDAVADDLFFSGVTSRSSSTWRYLFVWSFCGSRSVPKLRFKGYFLQRLEVTAAGIGEGKEEEELEWLVDKDAFPLVETMAPKPSLVEEPRAT<br>DESPSPRCFEQRGGAPRRGLGRWWCEGPEGRRMLCNACGMRYRSGRLVPEYRPISSPTFPPGLHSNRHSRVVQMRLLPRVKNLQLYYQAMMCSHTVPYS<br>RATWSIQSESKEHN*
BdGATA19	Bradi3g33200	A	MASEWEIAMGVELGMGMGAYNTTSSAGAAAPMGHHAGGGYHFYGMQPMGAADPSMRVDELLDLSSAGAGAHDFFPAAAADNGHYHYHHLGPGVGE<br>PSAATTPSATSSDHQTSMLSFADEFYIPSEEAAELEWLSKFVDDSYSDMPNYSSAAHAAMAKAAAAASNSPAGQHGSCITAAAPPGRGARSKRSRASAAA<br>AAAWHSLMPRPPSQSSPSSSSCSSSDIPASSNKPARPNNSNGSRGKKQGPPVADQSVGLVEGGVRRCTHCASEKTPQWRTGPLGPKTLCNACGVRFKSGRL<br>VPEYRPAASPTFLLTQHSNSHRKVMELRRQKEIVLIRGSHPSVPTGPAGAATVKPELLFRDYGIC*
BdGATA20	Bradi3g50160	A	MTHQTLISSAPAFSSASHLLHASSPSPPTLFRRSSSAAASVMSSFAHHHGSLVEKEGKMAALRSSLRPCEAAEDMAAAVAVGPAAWGAGLLGDGFLVEDL<br>LDLEDLCEVDKEGGGVFPEAAPAADEEKSGDDHSHGSSVVSYELLPLLPPEMDMDLPAHDAEELEWVSRIMDDSLAELPPQPQLPAAPSAAWQHRPRPRE<br>AAASSAPADPMRTPTICALSTEASVPVKAKRSKRSRATVWSLSGASLSDSASSSTTTASSSGSSSTSLSSFLLDSPAFAAGSSLKKKSKHGKQHKPKKRGRKP<br>KHLASSPFLASVPVPGDRRCSHCGVQKTPQWRAGPEGAKTLCNACGVRYKSGRLLPEYRPACSPTFVGTIHSNSHRKVLEMRRKKDPVVGFEAAAPTAVA<br>SF*
BdGATA21	Bradi3g54720	A	MASGRFMEEMMREHEQVLLEATLYDHIDDLLDFPKEEDCAADVLLLDAPAPGSPLSARIIGAPPVPPPPSMELAAPPPPAAAAVPAFFAAEAFGSKDCHIGP<br>CEELDMDMAQLEWLSGLFDDASIPHEPAFNCAAPIKASAMGATNAAAAVVIPDKLEDALFRSSSPTSVLEDNSFNNVGAGAKNNNNVNNGSGGSSASSSS<br>SSSASSCSESFSGSGGARTTWSAPVSPRPEPPVLIIPARARSKRSRQSAFVRSSAPAAEPTILVPTPMYSSTSAHSDPESCNIAETNSQPPAMKKKKKAKKPAPP<br>VTSDAEGDADADYEEGGGSALPPGAVRRCTHCQIEKTPQWRAGPLGPKTLCNACGVRYKSGRLFPEYRPAASPTFVPAIHSNSHKKVVEMRQKVAPKGD<br>DLLQYIRRRD*
BdGATA22	Bradi4g01140	A	MDVRRQPNVAAAAAAAAAATLGDLFPHQPAMDSGDNNTEWLSIYLEDCLSTHPVSAEQASQGAVKQKLPPPSSSNARRKKRSLASVIRDEEEHCFTVFVE<br>PPLLLPDQKHWLAESELILPKKDKDQELVQQQEQEHEEEKKRNASAEMLFQQEQMLVCSYCLSNQTPQWWDGPSGVLCNACGLRLQAGNEFSSMERCG

			QEISKEQEQGKRQEKRRIKRPAYIDEELPQKKRTKKTTYVNEELPPEEPVQRCTHCMSHKTPQWRTGPLGPKTLCNACGVRYKSGRLLPEYRPANSPTFSSY<br>MHSNSHKKVMQMRKSVEHSGQ*
BdGATA23	Bradi4g15720	B	MQQQQPLPPICATAGADDEDPLALDFEFDVVLPDSDDDLFAFTPGFDQVFTDDQTAAAAAPMQQQQEMEVAEAEEQVGEEQLVMGYDGRMFVFDSVQP<br>HKVETILSLLDGQELVPLPAQSTKPQLTYLVQPVVVPRDFDRPAALSRYRAKRQRKGLEPVVKADYSCRRDVALRMRRRGGRFVALSAKRTGDSPNELTL<br>CTNCGERSDATPKMRRGPDGSRTFCNACGLMWDKTGRLRETAGNQSCGGGRWCRRSESM*
BdGATA24	Bradi4g23550	A	MRKPGQYVYLHEDPLALDVVDGLDLSPPGGGLCSPDDPLDKVMTYVALLDDGFLEDLGIDCSTLLDEESSRSALEEGGGGALPVAAAAASPGGTKRSARE<br>VFDGVPGAPSSIPVSTTSAGPFLGTLEAPDFGEDLSWTVLPKPHHRRTAARRRQVWPVSFPLALTTSPGPAAAAHDDSNANDDVKDKDFKLISRCNVGKCN<br>GNKRRRLSEPKKRQRDKDNVEDKDFELVGYCNDVGVGNGKQRRSAEPKQQLDKDGRLRTCTHCCSWQTPQWRHGPNGNGTLCNACGIRYKMGKLCEE<br>YRPSTSPEFSSLKHSNRHRNVEKIRDQKMKQLKVTSPVPVVPVTPDSSEVLLRMCKYET*
BdGATA25	Bradi4g41570	A	MAGGGREAEGGGALYVLALAAMVAPALLVSRLEAAVPRRARSRLPRAVPSGWWSSRISFFPPPPPQPDQSEGQEKSPGPQRPAAAAKDPPEKQKKRKSYP<br>RPRPDPGAAGERPAKRPKRCLHCNAAETPQRRSGPMGRGTLCNACGVWYNKTGALPELVPVDPPVESPISEPEEPGAIYLVRRSAAERRPPRTEAPAPRPA<br>AVRRPPRAEASPVPVPAPRPAAVRRPPRAEAPPVPVPAPTPAADRRPPRTEAPAPRPATSCLNCGSSEPPELREGPMGRREVCTACGERYKKGRRLPECQPA<br>VRPVTDSPPHSPITANSPPDSPIWEPEAPRSSVCLARKSSKNGKTPWRPKDTGKSCMHCGSVKTPQWREGPMGRGTLCNACGVRYKQGRLLPEYRPMASP<br>TFLPSQHANHHRQVLRLHKQKPRSNDQEPSQLPPGTNGVNQKWESKDQEPSQLPPATHSVTKLTPIRDKHQTNMPDCSYKEPVCDDHPTDMPGCTDEEPV<br>EAPGCTHNPPIDVPRPSSLDSLLLDGPSAPLIVESDEFAIS*
BdGATA26	Bradi5g17057	A	MLHQTLIPSSSPSCPSVSASLSSPLLYPSNAKDTAAATTTASSSPLLCSPAMPSYAHHRSSLDERMEALKGSSRQEETAEEVAAAAEAPAAWGFGDRDGFSV<br>EDLLDLEEFCEADKDGADEHEAAPAAADNQEKSNDDSLQSVVSYDVVVPHAPSVPEIVDLPAHDAEELEWVSRIMDDSLSELPPPPPPPTATMMASLAGR<br>APQHRLMMLQQRRPHDGAYRALPSSASDPLRTPTICALSTEALVPIKAKRSKRSRASGWSLSGAAPDSTSSSSTTTTSSCSSSASFSPYFLMDSAHLGASDLT<br>EDYTLGGPPPKKYKHGKHSKHKPKKRGRKPKHLPPHPASAAVSFPSDRRCSHCGVQKTPQWRAGPEGAKTLCNACGVRYKSGRLLPEYRPACSPTFESTI<br>HSNSHRKVLEMRRKKEDGPLTVSATAPAVASF*
BdGATA27	Bradi5g17370	D	MVKEGPCRHCGVTSTPLWRNGPADKPVLCNACGSRWRTKGSLENYTPMHSRDDIDVEEPRVSKLKPPMSRLKEQRQLKKRPSHIIKKNEPFSDQNFRKMG<br>DADPSRSSSGSAVSYSESCAPYGSADASEMTGSAQSHAWESLVPSRKRSCVTRSKPSQVEKLVKDLNSIMHEEQFYCLSGSSEEDLLYHSETAVGSFEIGYG<br>SVLLRHANSKSVDGDSEANSVPADNKSYVTSESLSYSGTASFVVHGESKGASNSNALSEKPKWFPVQIHDNARRDKLHYSKPHTLENVDSALVSVALEVK<br>DSKEIGEKENISAVKCLVKPAMKHLKRPHESQLQSCQEVKETTRSPKRDKLSMLAPQYVDDSDQDLLLEVPPNGRHPEAELLCPLSQLSSVSRSSTSEGRVA<br>DGDGRLRQP*

BdGATA28	Bradi5g23656	A	MRRPNNNVFLSLHDVDDVASLHCDRSSSCRHGESALCCPDDSLDEVLNCIPPLEDELLEALGIDCSSPVLHGNPNNSAVDARTVVDVPPKSEHSLSVQEQL<br>VSPFHHWTIPKKPRGRAGTVRKRPWPHPVIVGATGYQFRHGSNGGPRQPTEPRPVIAAATGANQFAQGSNGGRRQPVNPRRGRRPGNEDENEPRACSHCK<br>STDTPQWREGPLGRRTLCNACGLRYKMGREKLVPEYCPSTSPFFRDGKHSNRHSKVEKLRKKEERASMESS*

Table S2-5 Sequence and information of GATA proteins in *Oryza sativa*

Gene	Gene id	group	Sequences
OsGATA1	LOC_Os01g54210.1	I	MEVTAEFGGAYYGGAAGREKKALQQGCGDHFAVDDLLVLPYGEEDETTREGEATGGKEEAAGFGNASADSSTITALDSCSNSFGLADGDFPGELCEP<br>YDQLAELEWLSNYMNEGDDAFATEDLQKLQLISGIPSGGFSTASVPSAQAQAASAAASMAVQPGGFLPEAPVPAKARSKRSRAAPGNWSSRLLVLPPP<br>PASPPSPASMAISPAESGVSAHAFPIKKPSKPAKKKDAPAPPAQAQLSSVPVHSGGSAPAAAAGEGRRCLHCETDKTPQWRTGPMGPKTLCNACGVRY<br>KSGRLVPEYRPAASPTFMVSKHSNSHRKVLELRRQKEMHQQTPHHHQPQVAAAGGVGSLMHMQSSMLFDGVSPVVSGDDFLIHHHLRTDFRPPI*
OsGATA10	LOC_Os01g74540.1	II	MDMDSSSSPVDKVDPDECNGSKACADCHTTKTPLWRGGPGGPKSLCNACGIRYRKRRRAALGLDSSATATATDGAEQQKKTKAKKEKAQEEEVTM<br>ELHTVGFRSKDAAVFKQRRRMRRRKCLGEEERAAILLMALSSGVIYA*
OsGATA11	LOC_Os02g12790.1	II	MSTIYMSQLPATLPLMEGDQDQGLYPAFHRAKDPPILFPFMIDSAVEHQGQIYGDQGLRRQQVLGESNQQFNDHMMMGGSDVFLTPSPFRPTIQSIGSD<br>MIQRSSYDPYDIESNNKQHANGSTSKWMSTPPMKMRIIRKGAATDPEGGAVRKPRRRAQAHQDESQQQLQQALGVVRVCSDCNTTKTPLWRSGPCG<br>PKSLCNACGIRQRKARRAMAAAANGGAAVAPAKSVAAAPVNNKPAAKKEKRAADVDRSLPFKKRCKMVDHVAAAVAATKPTAAGEVVAAAPKD<br>QDHVIVVGGENAAATSMPAQNPISKAAATAAAAAASPAFFHGLPRDEITDAAMLLMTLSCGLVHS*
OsGATA12	LOC_Os03g61570.2	II	MDSSSVEKGSGSIDPDERTASGEPKACTDCHTTKTPLWRGGPSGPKSLCNACGIRYRKKRREALGLDAGEGGAERQEKKKSKRERGEEVTMELRMVG<br>FGKEVVLKQRRRMRRRRRLGEEEKAAILLMALSSGVIYA*
OsGATA13	LOC_Os05g06340.1	II	MGSSDQKVIGIAAAAAAAAEEAGRRCCVECGATTTPMWRGGPTGPRSLCNACGIRYRKKRRQELGLDKKQQQEHHPHHHQQQQQQYQRQQQQQQ<br>QEDHSDAASSVKDSSSSSSNKSSSLQVISEIVISRSKSDCEGAMEGNCVPLKRLVQQVDFLLSSTGITESCQCVAVSCANQMGLQKAANVLLFLVPIRVL<br>TMENNSCDILHIIRIIGRGCGIESKTRIIDR*
OsGATA14	LOC_Os05g49280.1	II	MLQELAPCTCGMLYGSCGGGCGGAAAAASAFSLLFPMAGGQYYYRQCGGVAEEDSRSPYGGGGAAVDCTLSLGTPSTRRAEAGAYGGGLQPWDVP<br>SSARPGGGGGGKQDGAGVAPCNKEAPAAGRLPRRCANCDTMSTPLWRNGPRGPKSLCNACGIRYKKEERRAAAAVAPTPPPSLDTGAGYAYCYSRQ<br>PPPPPAPQWGCYGQAAAKSASYAMFDAADDGPCLSWRLNMMPSSPAFAVGERPGLFQYY*

OsGATA15	LOC_Os05g50270.1	II	MLHHYYSGGAGHHQDVAAAGSPGDMASSTFSLFFPMSNGQCWPPSTVEESAAYDDHSTVTTSPSSPSSSSTGSVDCTLSLGTPSSRRAEPVAAAAPAA<br>NHGAPVPAHYPSLSAATVSWDATAESYYCGQQGRPATGAAKCAAGAGHDALLDRRCANCGTASTPLWRNGPRGPKSLCNACGIRYKKEERRAAAT<br>TTTADGAAGCGFITAQRGRGSTAAKAAPAVTTCGEETSPYVVGGGGGGGEVADAAYLAWRLNVVPPAATATAFSVWPERASLYHYN*
OsGATA16	LOC_Os06g37450.1	II	MSTIYMSQLSAALPLMEGEHHHHHQDHHQGHFQAFSLQPKDPPVLFPFVISRRSSSSSPSDSTTLSYGSDHHLTQQQQHQHQAMLEPQNMIGGSSAGIF<br>ATPFPTVKSIRDDMIERSQFDPYDTEKLQASCGLAKVVAGGKWSAVPAAKMKITRKMGEPSSGVTGGAATTVAPKKPRRRPAQAYEDHGHGGAMGQ<br>AFGVIRVCSDCNTTKTPLWRSGPCGPKSLCNACGIRQRKARRAMMASGLPASPNAAGPKAAAHSGAAAVAAAQPKVKKEKRADVDRSSLPFKKRCK<br>VVQVEDHQTLPAATNAAAAAAMEETAESATVAPPPAPTTRGGTLVDSIGLSWSKTHAAATASCSFRPSPVAPGFAAAVQDEITDAAMLLMTLSCGLV<br>RS*
OsGATA17a	LOC_Os02g05510.1	V	MSGHHEAKPYQPRRGPAPADEEAAPAAAADEAEAEAEVEAMERYEQEQEYEEGEEGEEEEYEGGEGVPMDADASAAAVAGMDPHGEMVPVAGGE<br>AGGGYPHVASNTLTLSFQGEVYVFESVSAERVQAVLLLLGGRELAPGSGSVPSSSAAYSKKMNFPHRMASLMRFREKRKERNFDKKIRYTVRKEVAL<br>RMQRNRGQFTSSKSKAEEATSVITSSEGSPNWGAVEGRPPSAAECHHCGISAASTPMMRRGPDGPRTLCNACGLMWANKGTMREVTKGPPVPLQIVP<br>AATNDVQNGIVEATGVEQHNSAVEEAVSAANGHESQSGVA*
OsGATA18a	LOC_Os03g47970.1	V	MPDAAAAAAAAQDADAVMRDAPADAAAGGGDNDDDDGDDGTEEDEEEDDDEEGDEEELPPAEDPAAPEPVSALLPGSPNQLTLLFQGEVYVFESV<br>TPEKVQAVLLLLGRSEMPPGLANMVLPNQRENRGYDDLLQRTDIPAKRVASLIRFREKRKERNFDKKIRYAVRKEVALRMQRRKGQFAGRANMEGE<br>SLSPGCELASQGSGQDFLSRESKCQNCGTSEKMTPAMRRGPAGPRTLCNACGLMWANKGTLRNCPKAKVESSVVATEQSNAAVSPSGIDNKELVVPN<br>PENITASHGEVMGDSTPANEAEIGAPKAQSQ*
OsGATA19a	LOC_Os03g52450.1	V	MAAEPPADGRDPPADDGAAGDGAVESAAAEALLSAASEQLTLVYQGEVYVFDPVPPQKVQAVLLVLGGSDMPPGLVSMAVPTTFDEKSTTVAARRV<br>ASLMRFREKRKERCFDKKIRYSVRKEVAQKMKRRKGQFAGRADFGDGSCSSAPCGSTANGEDDHIRETHCQNCGISSRLTPAMRRGPAGPRSLCNAC<br>GLMWANKGTLRSPLNAPKMTVQHPADLSKTGDTDDSKANLCAEHNQTTMKTDTEMVPEQEQKADVLPPTKEEDSMATS*
OsGATA20	LOC_Os06g48534.1	V	MARFEEEHRALGAEEEYEEEEDELEEEEEEMEEDEDAQHHEGVGGEVAVPMDAEAAAQLDPHGGMLAASGAVQPMASNQLTLSFQGEVYVFDSVSP<br>DKVQAVLLLLGGRELNPGLGSGASSSAPYSKRLNFPHRVASLMRFREKRKERNFDKKIRYSVRKEVALRMQRNRGQFTSSKPKGDEATSELTASDGSP<br>NWGSVEGRPPSAAECHHCGINAKATPMMRRGPDGPRTLCNACGLMWANKGMLRDLSKAPPTPIQVVASVNDGNGSAAAPTTEQEIPAPATVNGHES<br>ST*
OsGATA21a	LOC_Os04g46020.1	IV	MGKQGPCRHCGVTSTPLWRNGPPDKPVLCNACGSRWRTKGSLTNYTPMHARDDIDAEEPRASKLKPPTLKLKEQKQLKKNPSHITMENGPFSDQNFR<br>KMGDPDLSNRSGSGSALSYSESCAPYGTADASEMTASAQSHAWESLVPSKRRSCVTRPKPSQMEKLAKDLNSIMHEEQLLYLSGSSEEDLIYHSATPV<br>DSFEMGYGSMLLRPNSKSLEEESEASSIPADNKSYITSESYSGSVSFVYSESKATSNQNVITEQPKKFLVQTSDNARRANLHTENQDTLENANSPLVSLH

			MEGKDSEETRVKTSASNRLTKSTMNPLKRPHDTHFQSSVELRGTMRSPKRVSKYGDAMGLKCQASFMPKPGNGKDLACSDRALNLFMLPPDKLSML<br>VPPQYANTDSDQDLLLDVPLNARHPEAELLCQPSQLSSVAHSSTSEAGNAGGEGRLKQP*
OsGATA22	LOC_Os03g08370.1	VII	MQFEDGGDVHVGAGEGEDGGRVTVDELTRCLRCGISANATPHMRRGPEGRRTLCNACGIAWAKGKVRKVIDSDTPMDNAMFAQMVPELSMEFDDE<br>DKAYEFYNRYAGHVGFSVRKSSSDKSAENITRSRTFVCSREGFRKDKKGAKEVKRPRPETRIGCPARMSIKITSDGKYRISEFVPDHNHQPAPPSTMHM<br>LRSQRVLTELQTTEADSSEESATPSRFSSCSLVKQAEVIRHTNFLPAEYRCSLCSKRKKNMQPGDAGVTVKYLQSMQLSNPSFFYAVQLDEDDKLTNIF<br>WADSKSRTDFSYYSDVVCLDTTYKINEHSRPLTLFLGVNHHKQISIFGAALLYDESEESFKWLFDTFKIAANGKQPKTILTDWSMAATTASAITAAWPG<br>TVHRLCPWQVYQNSVKHLNHIFQGSKTFAKDFGKCVYDYDDEENFLLGWNTMLEKYDLRNNEWIKKIFDDRDKWSPVYNRHVFTADIKSSLQSESV<br>RNALKKSLSPQFDLLSFFKHYERMLDEFRYAELQADFHASQSFPRIPPSKMLRQAANMYTPVVFEIFRREFEMFVDSVIYSCGEDGNAFEYRVAVTDRP<br>GEHYVRFDSGDLSVVCSCKKFEAMGIQCCHVLKVLDFRNIKELPQKYFMKRWKKDVKSASTGNQELLNGGVSQIPSSYLNVPVPFIDPQHVQSNNEL<br>NHDTSVSNSHQQALHGGAQGSQGYAPLAGIQQQQFIGNFRLNHETGFL*
OsGATA23a	LOC_Os07g42400.1	VII	MRVWLGRVGGGDAMMHMLVAPDGGGGGEMPPPYGGAAAAPPPPMEQELELHRDNADDGLDGHVRCLRCGISGNATPHMRRGPDGPRTLCNACGI<br>AYRKGKMRRMIEAEPPIDEAALAKLVPEVGMEFESEDKAYEFYNKYAGHVGFSVRKSTSHKSSGNITKVRTFVCSREGYNRDKKSLEAKKPRLDTRIG<br>CPARLIIKVTPESKYRVTDFKADHNHQLAPPSTMHMLRSQRILTELQSGEAELSDDSVMTPTTKATGDLVVRQIGFLRSISLLPADYKNYLRSKRMKAM<br>QLGDGGAILKYLQTMQMENPAFFYTMQIDEDDKLTNFFWADPKSREDFNYFGDVLCLDTTYKINGYGRPLSLFLGVNHHKQTIVFGAAMLYDESFES<br>YRWLFESFKIAMHGKQPAVALVDQSIPLASAMAAAWPNTTQRTCAWHVYQNSLKHLNHVFQGSKTFAKDFSRCVFGYEEEEEFLFAWRSMLEKYDL<br>RHNEWLSKLFDERERWALAYERHIFCADIISALQAESFSSVLKKFLGPQLDLLSFFKHYERAVDEHRYAELQADFQASQSYPRIPPAKMLKQAAHTYTP<br>VVFEIFRKEFELFMDSVLFSCGEAGATSEYKVAPSEKPKEHFVRFDSSDCSCICTCRKFEFMGIPCCHMLKVLDYRNIKELPQRYLLKRWRRTAKSANE<br>ENQGTLLFLICMRIHFAEAHDAGDLNLNIIPSARCLLHDTGVEAVRTGHAELPRIKVSNL*
OsGATA24	LOC_Os10g32070.1	V	MVVVDGLHDGGGGDLQALLDDAGVDDVAARGGGEVEEEVERPSNEDAFPAVEKMATAAAAKGLQCRHCGTTETPQWRHGPEGHRTLCNACSMR<br>YRSGKLVPEYRPLRSPTFSPELHSNRHHRVLQLRRRPGPQSAAPSPAAVARYGGEAKEEEEELAWVSNKDAFATVETTMAPSPRVVETPPEHDHRPAN<br>TPTTSPEPHSDRPRRVVQLPRRLQEPSASANLAHAVAATARAGRECAHCGTTKTPAWRLGPDSRRKLCNACGNKYRSGQLNSTTFSQNSQEQKKKSK<br>SSACSRERKRSAVAATVVVGGGLRDDAAAIADEHLDGGDLQALLDDVALDDVAARGGGDAGEAKEEEEELEWLSNKDAFPTVETMSPAPPENRTKA<br>PVPPAGWQCRHCGSTETPLWRERDGPAEAEHVRKEETPPNITPATKHRRIVDLLRCSTALNTAAKAVERRCTHCGTTKTPAWLSGPDSRGKLCNACG<br>KQYRKGRLVPEYRPLNCPTFSPELHSNAHAHRRRRESPVAIAIAGEK*
OsGATA25	LOC_Os12g42970.1	I	MDVHPPNAAASSLEELFPHQPATESDRSGIEWLSVYVEDCLSTSASCTNPVSAELPPITMASQGAAKPKLPPRSSTNARKKKRSLASVISDTDDQHCITL<br>FVEPPLLLLDHKDWLAESELILPKKDKDEELVQEQEQEEEENYKMSAGMQFQQEQLVITCSYCLSSQSPQWWDGPSGPTCDACRLRIEARNGHTTSSK

			KRYGQEIDKEQDIGKRRDKKKIKKAVYVNDELLSEEPMKRCTHCLSYKTPQWRTGPLGPKTLCNACGVRFKSGRLLPEYRPANSPTFVSDIHSNSHKKVMQLRNSVPHPRK*
OsGATA26	LOC_Os12g07120.1	V	MDGDGDVGGGGGGGGGGGVRYVLALPAMASLAVLIAHLDAAVPVPRRPRSYLPRAVPMAWWAFRLPVFRPPPPPPPPPPAKNPVKEEEGVARVVVVVAPPPPVDPGEEEAGKRAAKRARRCLNCDAVETPQWRSGPMGRSTLCNACGVRLRAVGSLPEHRAPAARTTTAAPASPPDSPIWTPGHKPPSSSPDIYLVRRTPKLPVTRPPRTKQAPPTAPAPAPPPPPPQPASPKTKTKAKAKKPKRKRSCVHCGSTETPQWREGPTGRGTLCNACGVRYRQGRLLPEYRPKGSPTFSPSVHAANHRQVLELRRQQRQSTNPSTPPPPPVSAAEPIPDEQKEEVVSVPVAAAAPATDGGAASSLDALLLDGPSAPLIVDGDDFLVS*
OsGATA27	LOC_Os03g03850.1	I	MRKPTPYVSLHDVVAFDFVDGDVPFDDLVDGEGLCCPDDPFEEVMRCLSAVDDPFLAAFKLDCSPPTPAADADVDSRSEEHMHADVGGGLDLQRAVGGGDEKAGTPSTVDDVPWLQASAVARKPRRAPAAVRKRVWSLVSPQLATAAAAAVDNSRDEVSSGGGGGGEGGEHCSRPAKRRRKCGEEKRCGHCQTTETPQWRVGPDGPSTLCNACGIRYRIDHLLPEYRPSTSPGFGSDGYSNRHRKVVKLREKKRKKAMLAATATALTSGPV*
OsGATA28	LOC_Os11g08410.1	II	MPKPTPSSSSFLDFTGGVDGDDDDPSCPFEGLCCPDDPLDQVLNFDSSDFGHVFFESLDVELFLPRGGPSRGAGEEDSKGAVERVAFGSSAAVESELGGVGGGGAGSEVSVPGGAGGGRGEDMETEALDVKPVVGVGAGGAMGAHVAGGVGAPGAFPESKQLVPWPCAVGAGASAPGAAPDNRLLALPDVRFDALTAEGAAPGGERGKTIPDSVSKNGLPTLPGVRSATPTAPPATPFRLEWDHAAAPSSSATTTPSDSSLSSPPSLSSVFPRIARVFPSRTKPRRRRTLRRQHWSLICPLHLVPVAAAADAARGKSISELNASASAATDAGTPSINDGGGGSYHRRVVGRQRNRQVRKDRRCSHCGTSETPQWRMGPDGPGTLCNACGIRSKMDRLLPEYRPSTSPSFNGDEHSNRHRKVLKLREKKGRD*
OsGATA2a	LOC_Os02g43150.1	I	MTHQALIPSTPPSAFSPASHFLHASSSSSPSLSSHAVVATAAAAMSSFAHHHHGSLVEKDGRMSALRSSLRPYEAAEEMAAAAAAGGPAAAWGAVERGAGMMGDGFSVEDLLDLEELCEVDRDGGEQGEAAAAAAAAVEKERSSDSHGSSVVSYEPMPLLPPVMDLPAHDVEELEWVSRIMDDSLAELPLPQLPAAAAALAACGKPQHRRPHEGAASALLDPMRTPTICALSTEALVPVKSRRSKRSRASVWSLSGAPLSDSTSSSSTATTSSCSSSASFSPFLQYVDFPALVASDLLDEQPRSKKSKHGKNGKQKPKKRGRKPKHQQPPHLAAAAGGGAALPATGDRRCSHCGVQKTPQWRAGPEGAKTLCNACGVRYKSGRLLPEYRPACSPTFVSSLHSNSHRKVLEMRRKKETPVIVAAAAPAVASF*
OsGATA3	LOC_Os02g56250.1	I	MAGVGFVEDMLREQSLLEATCGDLFDHIDDLLDFPKEESAADVLLLDAPAPGSPLSSRIIGGHATMAAAPPPPPQMMALPPPPAPAKDDASALFDAAGALGAEVFDRKDAHIGPCDELDMDMAQLEWLSGLFDDGTIPHEPSFPGVNCAAPIKASALTANAGVVLPDKAEEALFRSSSPISVLEHSGFNVATNGGSSSSSSSSASSSSESFSGSGRAWSAPVSPRPEPPVLVIPARARSKRSRPSAFPAVRGAPAATETTILVPTPMYSSTSSHSDPESIAESNPHPPPMKKKKKAKKPAAPAAASDAEADADAADADYEEGGALALPPGTVRRCTHCQIEKTPQWRAGPLGPKTLCNACGVRYKSGRLFPEYRPAASPTFMPSIHSNSHKKVVEMRQKATRTADPSCDLLQYIRRRD*

OsGATA4	LOC_Os03g05160.1	I	MVGDKDAAALAGELTGDAGASLNGFFDHTGLESAVVGEGQGEGEEEEELEWLSNKDAFPSVDTMAAEVESAAPGAPARAAVGPRTKGLRRRRRVT<br>APWSLAPLLSRPRQAAAAAADAGAPRRRCTHCAVDETPQWRLGPDGPRTLCNACGVRFKSGRLFPEYRPANSPTFSPLLHSNSHRRVMEMRLQSEED<br>ASAASRVNAKARRAERAAARLAGKDKK*
OsGATA5	LOC_Os04g45650.2	I	MDALKSSCRSEEAADEGAAAAPSAWGMVERDGFSVEDLLDLEEFCEAEKDAAEENEQALALVAAPEEEKSKDDSQPSSVVTYELVAPPPPPPEIVDLP<br>AHDVEELEWVSRIMDDSLSELPPPPQPPASVVASLAARPPQPRQLQRRPQDGAYRALPPASYPVRTPTICALSTEALVPVKAKRSKRSRATAWSLSGAP<br>PFSDSTSSSSTTTTSSCSSSASFSSFSPLLKFEWHPLGGTSDLPDDHLLPPGKKSKHGKNGKNKPKKRGRKPKQLPPHPSGAAASAPAPGDRRCSHCGVQ<br>KTPQWRAGPEGAKTLCNACGVRYKSGRLLPEYRPACSPTFVSAIHSNSHRKVLEMRRKKEVGSGLLTAAAAAAPAVASF*
OsGATA6	LOC_Os05g44400.1	I	MEVAAADYAGGVRVKKEAGGCGGSGDMFLVDDLLDLPCDEEEEETGLCGAYGGGGAGLGAGVVGGGGDDRAAGNASADSSTVTAVDSCSNSFSG<br>LADGDFSGGLCEPYEQLAELEWVSTYMGEETLPTEDLRKLQLISGIPAAPRAPPALAVSAVQLPAGGAGALPTEAPVPGKARSKRSRVAPCSWSSRLM<br>VLPPPPASPPSPASAVISPSESGTAAPAFPAKKAAKSAKKKDGPSPAPAPNAAAQAAAEGRRCLHCETDKTPQWRTGPMGPKTLCNACGVRYKSGRLV<br>PEYRPAASPTFVVSKHSNSHRKVVELRRQKEMQLLHHHQQPPPHVGAGGGGAAGGLLHVTSPLLFDGPTSSAPLFAGADEFLIHNRISPDYRRQAT*
OsGATA7	LOC_Os10g40810.1	I	MASEWEMAMGVDLGMGMSTYHNASGGIAAAPMMGHHGGGGGGGGYSAAHHHHHHYYGMPHQAAMGDAMRVDDLLDLSNTPGAHDFFPASAA<br>AAAAGDHGHHHHHHIGGMGEPSGATPSATSSDHQTSMLSFADDFYIPTEDAAELEWLSKFVDDSYSDMPNYQSSAHAAMAAAAASAANNGGGSSA<br>GQDSCLTAAPGRGARSKRSRATAAAAAAWHSLVPRPPSQSSPSSSCSSSDFPSSNKPSGTARPNGSGGGSRGKKSPGPAGAEVGMEAGVRRCTHCASE<br>KTPQWRTGPLGPKTLCNACGVRFKSGRLMPEYRPAASPTFVLTQHSNSHRKVMELRRQKELLIIRGSHRDAAAAAAAAAAAAAAGSAAATGRPELM<br>FRDYGVC*
OsGATA8a	LOC_Os01g24070.1	II	MGSTDRKVVGIGVAEEGRRSCVECRATTTPMWRSGPTGPRSLCNACGIRYRKKRRQDLGLDLNQPQKQEHGEVIPEVKDSNSNSNNCNSGSGNSSSN<br>LQVVPKRRLLMGVEEAALLLMTLSSPSASTLLHG*
OsGATA9	LOC_Os01g47360.1	I	MLHEAAPCTCGLLYGSCGGGCSLLFPAGAPGDHHHHHHYKQYCGAGDGEYPDVPYGGGGSVDCTLSLGTPSTRRAEAAVAGLPWDQSSLQPSCNGR<br>QEMSGAAAPRTEPSGGAGAAAASAPRRCANCDTTSTPLWRNGPRGPKSLCNACGIRYKKEERRAAAAAVAPTALASDGGVEYAYGYPRQQQQWGC<br>YGPAVAKAASFGMFGDAAGEDGPCLPWGLGVMPSSPAFGAVREMPSLFQYY*

Table S2-6 Sequence and information of GATA proteins in *Zea mays*

Gene	Gene id	Sequences
ZmGATA-1	AC184831	MRQLSYLSGLDGLDDGVADCDGSCGLRDDGPPCLLHSGLCCPIGPLDDETDTVAIAIDNDKELLQDLAVAAGRVAASPLSSGNSPSSSAVETTTTLPPTPWRFVVP RRKRRKRRDRRPVVMRGSIRPGIPTLHAAAMSSSSSSSGNNRPCSLTAETSSPPQLWRLVVPTRKRDRRPVVMRGSIKRPCSLGTTPGIPTPHAAGNSNPSSSSGGG STGARRQLESLQVRPRPPPTNRQRVQRVCSNCGSTETPLWRTGSDGSATLCNKCGLRLSRNRQAAQAS
ZmGATA-2	AC194965	MMMDSAEHQKVMGIAPAAAPAAEAGRPCCVECRTTATPMWRGGPTGPRSLCNACGIRYRKKRRQELGLDSAKKPQQNQRPPPPQQQQQQEDHCPATSAVAVR DNTKSSRLQVVKKRRVLMGVEEAAVLLMALSSSSRSTLLHG
ZmGATA-3	AC202864	MEVSAECAGGGSVKKEADLFLVDDLLDLPCDDDDEETVVGDWEGDGSKQAAVLDRGCGAGGEEGAAGNASKNESSAVTALDSCSNSISGSGLADGDFSGGLC EPYDQLAELEWLSNYLGEDNFPTEDLKKLQLITGIPPAATAMAPAPAPAAAQAQPAAGVLPQEAPVLGKARSKRSRVAPCSWASRLVVLPPPSPGSPPSAAISPSE SGSGTAALAFPARKPLKPAKKKEAPSPSLPPVPNNAAAAGAGEGRRCLHCETDKTPQWRTGPLGPKTLCNACGVRYKSGRLVPEYRPAASPTFVVSKHSNSHRK VLELQRQKEAHPHHHHQYQPQQALAHVGAGGTLNLMHAPSPLLFDGPAAPLIGDDFLVHSHIGPDFRQLI
ZmGATA-4	GRMZM2G009530	MSFFASQESDESNTEWLSGYVEDCFSSSTSYTNPVSAMASEGVGKPKLPPPPSSNGRRKKRSLASVMTNGDYQQFVLPLYVEPPLILIDQKHWMAESELIHPKKD DDDREVCQQQGQEQEKGVPRLVKMCSNCLSSQTPRWRDGPSGRQMMCSACGMRLKPETRLSAEQQCGQEEPTRKEQEPDRKRKKAAKKTCYESSDAPPSSDQ QEKRCAHCLSSKTPQWRAGPLGPKTLCNACGVRFKSGRLLPEYRPANSPTFVSCLHSNSHKKVMQMRQAVAQQQ
ZmGATA-5	GRMZM2G025002	MPLPPPNMRTQEPHPWLRDAPDDLPDCDGTGACFPGGGLCYPDDPLELVHSLFPAQTTVDRSALGIGTSAGEPPCREQEQPLAESAYGGGGSGGRNSCGLSSYVL EGLSGLEEIDTNMFFADDALDGGGGGGGGEATQDNLPCDSTGAKPKPPAHMPEAGDVHASPLPAHMLPGALQAYDACRAFHGASPPMAAGGPLPDFVTNDGA PMPAAAGLHACGALPGIVSNGASKPPSCRAFHGASPPTAAGGPLSVFVTNNGAPMPGAAAGLHACVVVLPGIVSIGASGPPRMEPMPARASLHPNAAPAHAPPPP SPPSSSASSGSGRCPSSATSGTEFLQPHAWVVPRRQRSLPTGARRSRSAPRQRNMQAQKVCRHCHSPDTPQWRTGPNGRATLCNACGLRYAGHRLVPEYRPLTAP SFRSGQHSNRHRNVMKLREQMKAAATEEEPSEQPTEGNT
ZmGATA-6	GRMZM2G031983	MSTVFMTQLPRTLRLMEGEQDQGLFPASTFHVAKDPPILFPFMITSPVHVDQLQGQSSYGDQHLGQHVLSESTQQFTDRTMISGSEVFPRRPSPFGQTIQSIDGDMI QPSAYDPYDIVENQRTEGLSGGGWTVSSSSSSPPAKMKIMRKATSEYPEGGAARKPRRRAQAHQDESQLLTMQQQAMGVVVRVCSDCNTTKTPLWRSGPRGPK SLCNACGIRQRKARRAMVAAAAAAAASNGGAPPQAVSGVATQQQPKPKPARKEKRSDAADRSLPFKKRCKMVVVDHAAGAAAATATPEAAAARSTKDQADH VSGDKQVVAPAAAMRSLDQSEIATPPPSAVSSFHAAFPADEITDAAMLLMTLSCGLVRS
ZmGATA-7	GRMZM2G039586	MIATSLSLLIPGCKLPFRHNNHCTSRFGSPLPTLHISHPDLHSSPNHTRSAYIAPSDLERKFLSVHPMSAIYMSQLSTALPLMEGDHQDHHHHHQGHFQAFTLPKETP ILFPFVISNSSASEGSLSYGSAADHHLLRQRHQAMLEPQHMIGGSSTATGNSVFSTPFPTVESIRDDMIEPASYDPYDMGKLQVGGSMDACSWTPAAAKMRITRKA TADPSAGKKPRRRAQAGYDDTMSGQPNLGVIRVCSDCNTTKTPLWRSGPCGPKSLCNACGIRQRKARRAMMAAASGSVSAVPTDSGKASPSNAAVAAAAAHP

		KVKKEKRVDVDRSLPFKKRCKVVQQGHGAAVVVAAPAAATDSATVVQATAEDGDDDTCPSRDLLVDDIGGLISWSRSPAAPASAAAATCSFRASPALPVQQDE<br>ITDAAMLLMTLSCGLVRS
ZmGATA-8	GRMZM2G044576	MEAAASAEYGYYGGGAGPHETKTAGCGDHFVVDDLLALPPYDDEEEGATGETPLCLQPVKEEEGGLGNFSADSSIVVTAIDSCSNSFSRLADDDFPGEFYEPYDQ<br>LVELEWLSNYMGEGEETFAAEDLEKLKLISGGFSPAAVNVSAPAPVGVASAASATQSGMFLPVPAKARSKRSRAAPGNWSSRLVVLPPTPASPPAPAASMAISPS<br>ESGISAQQAFRAKKPPPSKKKDAAAPAPAPAEGRRCVHCDTDKTPQWRTGPMGPKTLCNACGVRYKSGRLVPEYRPAASPTFVMSKHSNSHRKVLELRRQKEV<br>VVQQPPHVMGGAGGPAGGLMRMQSAMLLDGPVAAVSSSSPIVGGDEFLIHQHLGTADYRQRI
ZmGATA-9	GRMZM2G048850	MTSLSMPEQVHGHSPQETVAMASDEPQSQLTGLGDPKASRSDAATMVTTGGAGDGEEVMGAESPLDFVLVGTPWGLLLAPTPLASMKQDEEEVSAPPCPEKTP<br>AAANGAKQCVQCGATETPQWRIQPTGQGALCNACRIRLRPAEALREKVHVHAHRPSAPALGPATSTAISEPPADGWISGAWMDFDAHLLKVTPPPPARRQESPS<br>PTTEPAPEKKTRKRKPSTRRCQAASTAASRPRRH
ZmGATA-10	GRMZM2G052616	MGSADRSKIDGIVVPEKGARSCVECRATTTPMWRSGPTGPRSLCNACGIRYRKKRRQELGLEHNKQQQQQQNNGEAKTGVKDSSSNSSSGSSNFHVVQKRRLL<br>MEVEEAALLLMTLSSSPASTLLHG
ZmGATA-11	GRMZM2G054615	MLHQTTLIPPSVSAASSSPFLHAATTAPAPGGGSCTPAFLRGASAMPSYAHHRSSPLDDGRMDALKCNNSVLPEETTADAAGAFVEKDGFSVEDLLDLEEFAEPD<br>KDVAEREDDDAPPAAAAAAERSKADSQPSSVVKYDLPPLLPPPPEMVDLPSHDVEELEWVSRIMDDSLSELQPQAQPKPAAAVVASSAARPPLAQQRRPFAHDG<br>TYRAVAAAPPQAGPQRTPTICALSTEAMIPVKAKRSKRSRGPGWSRPGASFLPDSASSSSTTTTSSCSSSGSISPFLRLDSSPFGGGLELGEGLFSYGHLLPRPPSKKS<br>KHGGKGSKHRPKKRGRKPKHLPPPHPSAAASQPGPSDRRCSHCGVQKTPQWRAGPEGAKTLCNACGVRYKSGRLLPEYRPACSPTFVSSIHSNSHRKVLEMRRK<br>KEGGMVATAAPAVASF
ZmGATA-12	GRMZM2G058479	MSHHDGSKPYQPRRGPERHPQPADGIAAPPPAAVAPSVEHLVAAAAEAEALNRFAAEQQQQLQGHEQEVGEEEEEEDEQEDEMEEEDEDEHEGQHGGIGGEHV<br>PMDADAAAAAAAAAVSQMDPHSALVAGTVPPMATNQLTLSFQGEVYVFDSVSPDKVQAVLLLLGGRELSSLGGASSSAPYSKRLNYPHRVASLMRFREKRKER<br>NFDKKIRYSVRKEVALRMQRNRGQFTSSKPKPDEIAASEMASADGSPNWALVEGRPPSAAECHHCGTNATATPMMRRGPDGPRTLCNACGLMWANKGLLRDV<br>TKSPVPLQATQSAPHLDGGNGSAMSAPGSELENAAAAMTNGHESSSSGV
ZmGATA-13	GRMZM2G065896	MAAEPAAADHDLRPPLADGAAAAGVGAASLAAAAGAAEALMSATSEQLTLVYQGDVYVFDPVPPQKVQAVLLVLGGYEVPPGLVNMAVSSANDEKNTTVAA<br>RRVASLMRFREKRKERCFDKRIRYSVRKEVAQKMKRRKGQFAGRSDFGDGACSSAACGSPANGEDDHFRETHCQNCGISSRLTPAMRRGPAGPRSLCNACGLM<br>WANKGTLRSPLNAPKMTQQLLANPCNMVDTDDKNSNVLPVEHNQATPKTDSMMPKEEQKLDIRLPTEEDTKAVS
ZmGATA-14	GRMZM2G067171	MAMVDEGGKGEAQPPGLDPRNRRSAATRATGDMDGGGRGEEWEGPPGYVLSLPAAALPLPVAVSCLDATVRRKGRSHIRHRGHPSGWWAFRLPVPAPEEAKR<br>PVPPASAANNPSEEDRSCPRHQRLPVRQAPAPGPHTPAPAEERPAKRVRMCLQCGAAVTPQWRSGPMGQGTLCNACGVRLKAAGALRGQVKHRPTPATARTPA<br>RPPLDSPASESSPDSPILEPGSVPDVYLVRKKPLKRGRPPPPPRTVPAAAPPLAPAVYLVKKKKKSASARKPWRPPKSAKQCLHCGSSSTPQWREGPLGRSTLCNA

		CGVRYRQGRLLPEYRPLASPTFEPSEHANKHSQVMQLHRQRKSQGQHHPLPAEHPRAMDVLQFPQRWQVKEEYPPTPLHQPLPHPVVDGSLASGELRVGDMVD<br>AAADAGHGGGGGKGSDLNNNAPSSLDSRLLEGPSAPLLVDGDKPLVH
ZmGATA-15	GRMZM2G077002	MGKQGPCRHCGVTSTPLWRNGPPDKPVLCNACGSRWRTKGSLANYTPMHRKDDIDDDEPRVSKLKPPTSKLKSQKKKPNHIIMENGPFSGQNFRKMGDVDQSY<br>RSSSGSAVSYSESCAPYGAADASEMTGSAQSHAWESLVPSRKRSCVTRPKPSPVEKLAKDLNFIMHEEQLYYPSGSSEEDLLYHSETPVGSFEMGSGSVLLRHPNS<br>KSLEKESEASSIPADNKSYITSESYSGSASFAIHNGNKAAINLNASNARLKKSPLHMEDNARRGVGSISGPEGFTKSTMKPLKRPRDTQFQIDAELEGTMRSPLRGL<br>KSGALAQFESSSLPKSGYTTKDSTCTGGALNLFMLPPEKLLVVPPQYVDPDQDLLLEIPLNARHPEAELLCQPSQLMSSISRSSTSMGGVAEGEGCLKQP
ZmGATA-16	GRMZM2G080509	MSHSHHDGSKPYQPRRGPERPPQPADGIAVPPPAAVAPSVEHLVAAAAEAEALSRLGAEQQQLLQGHEQEVGEEEGEDEEEDEMEDDDDDDDEQEGQHGGIGV<br>EHVPMDADAAAAAAVAAAGAQMDPHSVLVPGTVPPMATNQLTLSFQGEVYVFDSVSPDKVQAVLLLLGGRELSSLSGASSSAPYSKRLNFPHRVASLMRFREK<br>RKERNFDKKIRYNVRKEVALRMQRNRGQFTSSKPKPDEIAASEMAAADGSLNWALVEGRPPSAAECHHCGINATATPMMRRGPDGPRTLCNACGLMWANKGL<br>LRDLSKSPVPLHSIQQSAPILNGGNGSAMSALGSELENAAAAMGNGHEP
ZmGATA-17	GRMZM2G101058	MASEWEMAMGVELGMGMGGTYHHNASSITTAPMMMSSHPHSHSGGAASYSTPHHHHYYGGMPPMGDAMRVDDLLDLSTPGAGAHEFFPTAAPATDKGHHS<br>SGAMGEPSPTANSSDHRTSALSFADEFYIPTEEAAELEWLSKFVDDSYSDMPNYSSATHAAMAAAAAAAANAAGNGGGTTSAGQDSGVAAAPGRGARSRRSRA<br>TAAAAAVWHSLVPRPPSQSSPSSSSCSSSDFPSSNKPARPNGGSRGKKVPGPPGGGPAGGEAGLEGGGVRRCTHCASEKTPQWRTGPLGPKTLCNACGVRFKSGR<br>LMPEYRPAASPTFVLTQHSNSHRKVMELRRQKELILIRGSHRDAAAGSAAGGPRPELMFRDYGVC
ZmGATA-18	GRMZM2G104390	MSSAAGRAPAPTPMGSADRCKIDGIVAAEKATRSCVECRATTTPMWRSGPTGPRSLCNACGIRYRKKRRQELGLDRKLQQQQNNGEAKTDEAKDSSSNSSSGSS<br>NLQVVQKRRLLMGVEEAALLLMTLSSSPTSTLLHG
ZmGATA-19	GRMZM2G110295	MASEWEMAAMGVELGMGMGGTYHHNASSITTAPTSSHHSGGAGYSAAHHHHYYGMPPVGGDATAMRVDDLLENLDLSTGAGAHEFFPTAAAANKGHHHSG<br>GAMVGEPSPTANSSDHQTSLLSFADEFYIPSEEAAELEWLSKFVDDSYSDMPNYSSATHAAMAAAAAAAAAAANTAGTRAGGTSGGQDSCATAAPGRGARSKR<br>SSRAAAAAWHSLVPRPPSQPSPSTSCSSSDFTPASTNNKPARPSNGGSRGRKSPGPAGEVVAVGVEGGVRRCTHCASETTPQWRTGPLGPKTLCNACGVRFKSGR<br>LVPEYRPASSPTFVLTQHSNSHRKVMELRRQKELVLIRGTHRDASAAAAGSAGPELMFRDYGVC
ZmGATA-20	GRMZM2G113098	MASEWEMAMGVELGMGMGTYHYHHNASSITTAPMSSHHSGGASYSTPHHHHYYGMPPTGGAGDAMRVDDLLDLSTGAGAGAHEFFPTAPAPATTDKGHHHP<br>GAMGEPSPTAANSSDHQTSLLSFADEFYIPSEEAAELEWLSKFVDDSYSDMPNYSSAAAHAAMAAAAAGNGGGGTSAGQDSCVTAAPAGRGARSKRSSRAPAA<br>AAWHSLVSRPPSQPSPSSSSCSSSDFPSSSNRPARGRKSPGPGGDAVAGSDGGVRRCTHCASEKTPQWRTGPLGPKTLCNACGVRFKSGRLVPEYRPAASPTFVLT<br>QHSNSHRKVMELRRQKELILIRGSHRDAAAAAGAGSAGPRPELMFRDYGVC
ZmGATA-21	GRMZM2G114775	MDSSVEKQGSVALDPDERAPASGETKACTECHTTKTPLWRGGPCGPMSLCNACGIRYRKKRREAMGLESSSKAATAGGSEHQQQQRKKKATAAAAAASSKRE<br>RERERERNKEADEVTVELRAVGFGKEVVLKQRRRMRRRRRLGEEERAAILLMALSSGVVYA

ZmGATA-22	GRMZM2G118214	MAMADDEDKGEPPGLDPRTRRPVATGDMAGGGGEEEWEGPPGYVLSLPAVAPLLPVAVSCLDATVRRKRRSRPRLRAQPSGWWAFKLPVPAPEEAKGPVLPASVVNNPSEEARPQSLCVRQAPSPDPYTPAAAEERPVKSVRMCLQCGAVVTPQWRSGPMGQGTLCNACGVRLKVAGALRGQVRHRPAPRTAARPPPDSPASESSPDSPIWEPGSVPDVYLVRKMPLKQGRPPPPSPMPAPPPPAPAVYLVKKKRKKTPASVASAKKPWRPPKSAKQCLHCGSSSTPQWREGPLGRSTLCNACGVRYRQGRLLPEYRPLASPTFEPSEHANRHSQVLQLHRQRRSGSQSHHHQQQHPLPVEKHPPRPTAALQFPQRWHVKEEYPPTPPHQPLPHPVVAGSLAAGELRVGGMVDAAADADADAGHGGGNGNGLSNAPSSLDSLLLEGPSAPLLVDDDDPLID
ZmGATA-23	GRMZM2G123909	MLHEAAPCTCGLLYGSCGGGCSLLFAAAAAGADHYYKHCGGGDGEAFSGGLYGGSVDCTLSLGTPSTRRAEAGAAARAPAGLHSWEAPAPSCNGGRQQEARGAEAGARRCANCDTTSTPLWRNGPRGPKSLCNACGIRYKKEERRAAAAAVAPAADGGVDYAASYGGYARQPQQWGCYGPAAAVAKAASLGMFGVDAAAEVVDGPCLPWGLGVMPSSPAFGAVREMPSLFQYY
ZmGATA-24	GRMZM2G135381	MEVSPECADGGRVKTEVDLFLVDELLDLPYDEEEDAREAVVGDGEAAGDGGKQASVLHRACGDGGEEGAAGNASNDSSAVTALDSCSNNSLSVSGLADGDFSGGLCEPYDQLVELEWLSNYMGEDNLPAEDLKKLRLINGIPPAATATAPAAAAQAQPPADGALPPEAPVPGKARSKRPRVAPCSWATRLLVLPPTPASPPSAAISPSESGTAAPVAFPAKKPSKPAKKKEAPTTPVPDNSAGAGESRRCLHCETDKTPQWRTGPLGPKTLCNACGVRYKSGRLVPEYRPAASPTFVVSKHSNSHRKVLELRRQKEAHLHPHPHQYQYQPQPQPQPQAFVHGGGGALVHAPTPLLFDGPAAPLIGDDHFLIHSHIVGPDFRQLI
ZmGATA-25	GRMZM2G138967	MGMEIDMETYPNLNTSPSAATASGDAKACANCHTTKTSLWRGGPEGPKSLCNACGIRYRKRRQAIGLDAGAAAAANSQQDLQQPKKKAAVDPQQQDQHQLRKKTTAVANPQQDRHQPRKRAAAAAAATDPQHTSITKKDTDKKDQQVTVDLHVVGFGKEATFKQRRRMRHNKCMSEEERAAVLLMALSSGVIYAS
ZmGATA-26	GRMZM2G140669	MASFVVHHHGSPLDREGKVKMSALALRSSLRPCEAAEEVGAGQAWCRGADMGIAAFADEFSVEDLLDLEDLCEVDKDCAELGDTVPAAESVQEEDKVSSDSHGSSVLSYELMTLPVPHPMIDLPLPAHDAEELEWVSRIMDDSLAELPPPPKLPAAPLGAARRPLEGAVAGPMRSPTTTICALSTEALVPVMRAKRSKRSRGSVWSLSGGAPLSESTSSSSTATTSSCSSSASFSPLLLLPADPSLVLSSHLLDETPRPKKKSKHGKHGKSGKPKKRGRKPKHHPKPPQFSGGAAYAPAQGDRRCSHCGVQKTPQWRAGPEGAKTLCNACGVRYKSGRLLPEYRPACSPTFVSSIHSNSHRKVLEMRRKKDGDLGQLPLPVPAALLLGPAVESF
ZmGATA-27	GRMZM2G163200	MAGGGFVEDMMREQSLLEATCGDLFDHIDDLLDFPNEDSAAAVLLLDAPAPGSPLSARIIDVGRAGNALAPPAAPAQHDASASAFFAAAGNDVFDTKDVVGAHIGSCDDIDMDMAQLEWLSGLFDDASIPHEPVFPGATAGGAAPIMKSSALAAGALLPQDKMEDALLFRSSSPISVLEHGSGGSGSASATGSASSSSSSASSSSESFSGSGSGSGGGHVWSAPVSPRPAPPPALVIPARARSKRSRSSAFTGAAARAGAETPTILVPTPMYSSGSAHSDPESIAESSPHPAPPMKKKKKAKKPVVPAPAASSDDNDGDADYEEGGEQTEPQGGAVRRCTHCQIEKTPQWRAGPLGPKTLCNACGVRYKSGRLFPEYRPAASPTFVPSIHSNSHKRVVEMRQKAVRSGDPSCDLLHFIRRRD
ZmGATA-28	GRMZM2G324131	MREQERRSPPMETTRTASNEPQLPESGPTTGRSAAAMATGDMAGGGAGEGERGPDSVLPSSPWVALLSVVGSHIARPEFQRKPRSHAPRAAPARPWGSKFPALPPVAPAKMKPTRKVRHKRRQRVHRAPPLPAAAMKRLAKKRARVCTLCGTTQTPSWRTSPADRLVMLCNACGIRARTSGAAPPEQVHVHVHLPTPATTVVVSDPEQPAPLEILQGSEYEHLPTAATVVVSAQEQPPPQEEIQQRSESPPDSPYLNSMLDFDVYLLKRTHPRREKSPPPPPPPTAGITAAALAPPPKGKKDKQKKKWCLHCGT

		TWSLQWRTGPMGVSTLCNACGVRYRQGRLVPEYRPRASPTFDQSEHSYKHRKVLQLREMQDRPAPPAAFRPSGNRKKRRKGKEQQQHQPAQARSRSEEQDQQPAQPAAVAVLQVRKKSDKGKAQRLPDPQPALRLKLRKELRGKQQHLLPPLPPPPPPPALPPHAGYDYDMVQQHLPPSPPVHPDPHAGYDMLHPHAGYDMLQSSYSYLPLPPALQHAAGDDDDMEMEQQHLVLLPPAAFLLRAGDEMAQYLPPPPPPPELPHAAADDDMEMEWRLLSLPPALPHATDELMVGEPMPIPPLDPFLFDGPAAPRIIDDDDDDEPATVIVIDDDEPAPVIVVDDDDDDVPVTVIVVDDD
ZmGATA-29	GRMZM2G325850	MLRHCNTTTQQQHQRHRHRQSIPATALAPATMPSTTFSLFSPLPCSTKAHQYAPADWFDDDDSNSAVIATPPSPSSSSTGSVDCTLSLGTPSSRLAAPKQRPLPASSCGTMPWDVAAAADQSCCCCRRRQGSSSSSRPSVVVNKSAVMPCAGQDPLLVDRRCANCGTSSTPLWRNGPCGPKSLCNACGIRFKKKERRAAATAAMDQGGCAYVAERAQYGAAAGRAYYGSCEGPAFPCDGGDVADAEAAPQNFLAWRLDVVAPAQAQAAAFAAVWPEQTTLFQYN
ZmGATA-30	GRMZM2G379005	MDMDMAQLEWLSGLFDDASIPHEPAFPGAAPIIKSSALAAGALLPPDKMEDALLFRSSSPISVLEHGSFNNANGGGGGCSASSESSSASSSSESFSGSGSGSGEGHAWSAPVSPPPVLVIPARARSKRSRPSAFTRAGAEAPTILVPTPMYSSGPSHSDPESIAESSPHPAPPMKKKKKAKKPPAPPAPASSDDNDGDADYEEGGERAEPQGGAVRRCTHCQIEKTPQWRAGPLGPKTLCNACGVRYKSGRLFPEYRPAASPTFVPSIHSNSHKKVVEMRQKAVRSGDPSCDLLQFIRRRD
ZmGATA-31	GRMZM2G396451	MGVDAAASARLLPAMGVTWRFGPQAIHEGLHVPLLQMRFTNSKLLIVNLSQQRAMAIGGLLGGPGRCTVVSMDEIKRTVPCWSVSSPFVNIGIYRSSPKQLRSDSARYLEWSLSMLGKENREAPLRIACPPGVPLDLVSNFEIKSFDGCANPHLGLAAVVAAGIDGLRRHLKLPEPIESNSSDHSSKLKRLPQNLQESVESLSVDKVLHELIGDKLVTTAIAIRKQGIKYSNFSDLCQKQEAVKEVLGSLAKFACVLFPNIPEAYLDRWCDVYSFGVILWELATLRMPWSGMNPMQVVGAVGFQDRRLDIPKEVDPLVARIIFECWQNDQQRTGIYLVHLKAGARASTTHRAPAPAPARNVFLPAIPPPPTPSLPFPASRPRRPSLQSRPRQRRVPAAHLSHLFLSLALLTRRRSVGCRLSCLLARPLFMVMVDEGGKGEAQPPGLDPRTRRSAATRATDDMDGGGRGEEWEGAPPATCSRSRRRRCPCPSSSPAWTPPSGARGATISATVAIRRAGPAAPAPGPHTPTPAEERSAKRVRMCLQCGAAVTPQWRSGPMGQGTLCNAYRVRLKVAGVLRDQGRLLPEYRPLASPTFEPSEHANKHSQVMQLHRQRKSQGQHHPLPAEHPRAMDVLQFPQRWQAYTALRSAGKSVEIIFVSLDRDEASFRDHFQGMSWLAVPFDAAGLLRQKLCARFAIERIPALIPLSASATPSSGLGCGEDAVRLVGEYGVDAYPFSAQRRRELESMDDARRGGGRLQELLGCEERDYVISADDIKIPIADLAGKTVGLYFGAHWCPPCHVFTKQLKEVYNELKILRPVSFEVIFVSIDRSKGEFQASMSSMPWLAIPYSDAVRKKLTRIFVVKGIPGLLILGLDGKALKTDVHRPELLPTYEGSRCNKIKGIKAPRITPKNVIRFSSYTDTFYFITPEAWYWSEYLYLKKESLTVVISDVKCDDLKEEGKVQQTKRGNSIHDHSSSHSIRSFSALTESGPGLTSVHPSSRTRPPPFYALLPRSDGPFAGDEHTPQQELSGVEQREARDPMAAAEGQWVLMATGRTPTNIAVIKYWGKRDEALILPINDSISVTLDPDHLSATTTVAVSPSFPSDRMWLNGKEISLLGGRFQSCLREIRKRARDFEDKEKGVKIKKEDWDKLHVHIASYNNFPTAAGLASSAAGLACFVFTLGKLMNVKEDYGELSSIARDLGVHAAVYMVVL
ZmGATA-32	GRMZM2G397616	MAAASPAFSLFFPLPNTKAFQFDDDSHSSVTTCPSSPSSSSSTGTVDCTLSLGTPSSRRAAAKPRPPCLALPSRSAVSCDVAAPADQSCCCRCCSCPGGRRPSPAAANKRAAAAHGHGQDPAPLVDHRCASCGTTSTPLWRNGPRGPKSLCNACGIRFRKKERRAATGTTTADMDQGGCYLAQRAQYGAATSGRAYYGGGDGDIADAEAVPAQFLAWRPSVVEAAEFAAVWPEPATLFQYIN

ZmGATA-33	GRMZM2G404973	MPSYAHHHSSPLDDTRMDALKCNNNSAPPEETAEDADAAGALVEKDGFSVEDLLDLEEFGEPDKDGAEPEDDDAPPVPAAAEERSKDDSQPLSVVTYDLPSPPPEMVELPSHDVEELEWVSRIMDDSLSELPPQAQPPPAVVASLAGRPPLAQQRRPFAHDGAYRAVAPPPGPLRTPTICALSTEAMIPVKAKRSKRSRGPAWWRSGAPFLSDSASSSSTTTTSSCSSSGSFSPFLRLDSSPFGGLEVGEGYYGHLLPRPPSKKSKHGAKGSKHKPKKRGRKPKHLPTNSSGAGAAASQPGPSDRRCSHCGVQKTPQWRAGPEGAKTLCNACGVRYKSGRLLPEYRPACSPTFVSSIHSNSHRKVLEMRRKKEGDMVATAAPAVASF
ZmGATA-34	GRMZM2G421212	MGKQGPCRHCGVTSTPLWRNGPPDKPVLCNACGSRWRTKGSLANYTPMHRNDNIDDDEPRVSKLKPPTSKLKSQKKKTNHIIMENGPFSGQNFRKMGDVDPSYRSSSGSAVSYSESCAPYGAADASEMTGSAQSHAWESLVPSRKRSCVTRPKPSPVEKLAKELNYIMHEEKLYYLSESSEEDLLYHSETPIGSFEIGSGSVLLRHPNSKSLEEESKTSSIPADNKSYITSESYSDSASFVVHSGNKAAINLNLPTARPKKSPLHMEDNARRDKLHYENQHVLESVDSPLVSVDLEDVINYTNFMKYLTKEDRRQLLKFLPPVDSLTPPESLRSMFSCIQFSDAIDSYQMLLREGILDPSLCGDEEWKKVKTLALTNLTKCSWLECYKQKKGAKETGGVGGISGPEGFTKSTMKPLKRPRDTHFQSDAELDGTMRSPLRVLKSGALALQFESSSLPKSGYATEDSTCTGGAPNLFMLPLEKLPLLVPSQYAVSDQDLLLEIPLNARHPEAELLCQPSQLMSSITSSSTSMGGVAEGEGYLKQP
ZmGATA-35	GRMZM2G464037	MSNQPPHASLQDDLPDCDGDDPLALAIRLFPAHTTGAGLSPAALGIGRVAEPPRREQEPLANSTYGVRGAGPDPWGLRLSRSVLGGLDGFDVDTFFADDALDGGGEATEGDSPPAHVTAAARGSLANDDASPLPAAMPAVALEAYDACSAPVPAACGGAPPVLVANNSMPPASCLGARGALYIVSKRTRTHAPPPLAAESMTPAVAPPPAMPDGGNGGNNRSAALPVALAPPSGSTGGAVRRRRPVPRPRNREVQRTCSHCQSSKTPQWREGPDGRRTLCNACGLRYKSHRLVPEYRAAESMTPRDLHPNAGLVGASSGPSSRPSSSSTTTPSQSQIDFPSHALPAVAPPPAMPDPDGGNGGNNRSAALPVALAPPSGSTGGAVRRRRPVPCPRNRQVQRTCSHCQSSETPQWREGPDGRRTLCNACGLRYRSHRLLPEYRPTTSPSFQIGQHSNRHRRIMQIREQNGTAGS
ZmGATA-36	GRMZM2G532534	MAGCGAAADENPAAEELASGADASLNAFFDHAGLELAAAAGGGQGADDDEEEELEWLSNMDAFPSVETMSAEVEAAPSAAPPARLEPLPHASHAVGPRTKGLRRRRRVTAPWSVPPVLPPPAGSGAPRRRCTHCASEETPQWRQGPAGPSTLCNACGVRFKSGRLFPEYRPILSPTFSPLLHSNSHRRVMEMRRHPVGIGWDGTEESQSWRAWEESIHGGRHVCHSTAFAFCFAWEEHTISTGFCFRLPMSDEAGKSRSSSTYHTRKRRRDGRLRCTVRGYMLLPRLSVAVQRFLSFFFPPGTGSGDGDWQRARGRRSQVAAF
ZmGATA-37	GRMZM5G879778	MKPTPSPIAIDNDKELLQDLAVAASLSSGNSPSSSAVETTTTLPPTPWRFVMPRRKRRKRRDRRPVVMRGSIRPGIPTLHAAAMSSSSSGNWPCSLAAETTPPPQLWRLVVPTRKRGRRSVVMRDSIKRSCSLGTTPGIPTPHVAGNSNPSSSGGGSGGGSTGARRQLESLQVRPRPPPTNRQRVQRVCSNCGSTETPLWRMGSDGSATLCNKCGLRLSRNRQAAQAS
ZmGATA-38	GRMZM5G887975	MGLPAEQWYCYHEEGGASPYGAASNPDRLHAVARHAVHAPGRISRRRRRRRLDGGAPPRLSAPASLCNACGIRYKKEERRRRTSAAGTLDRRAPPQQQWGCYGPGAGKSAASYGMYDGDVGVVAVDGPCLSWMLEVVPSSPAFAARERRTLSPYY

Table S3. Analysis and distribution of the conserved motifs in GATA proteins of *S. italica* and other species.

Supplementary Table S3-1 Analysis and distribution of conserved motifs in Setaria italica GATA proteins.

Motif	Width	Best possible match
1	32	SHCGTTKTPQWRAGPEGPKTLCNACGIRYKSG
2	34	RLLPEYRPAASPTFVPSKHSNSHRKVLELRRQKE
3	40	RVASLMRFREKRKERNFDKKIRYSVRKEVALRMQRRKGQF
4	15	DEEELEWLSNYMEED
5	31	CGHCGAVETPQWRSGPMGPRTLCNACGVRLK
6	50	NYTPMHRKDDIDDDEPRVSKLKPPTSKMKSQKKKANHIITENGPFSG QSF
7	19	IGDDFLIHNRIGPDFRQLI
8	33	NQLTLVFQGEVYVFDSVSPDKVQAVLLLLGGGE
9	20	LDEVEEAAILLMALSSGLVY
10	48	WASRLLVLPPPPASPPSPASAAISPSESGTSAPAFPAKKPSKPAKKKE

Supplementary Table S3-2 Analysis and distribution of conserved motifs in GATA proteins of sevent species.

Motif	Width	Best possible match
1	29	HCGTTKTPQWRTGPLGPKTLCNACGVRYK
2	34	SGRLVPEYRPAASPTFVSSLHSNSHRKVLEMRRQ
3	50	RLNLPHRVASLMRFREKRKERCFDKKIRYSVRKEVALRMQRNKGQF TSSK
4	41	PAASNQLTLSFQGEVYVFDAVTPDKVQAVLLLLGGRELPPG
5	15	DLAELEWLSNFVDDS
6	50	SELTGPAQSHAWESLVPSRKRTCVTRPKPSSVEKLTKDLNTILHEZQS YY
7	18	EEEZAAILLMALSSGSVY
8	11	VDDLLDFSNDD
9	11	GKARSKRSRAA
10	50	YEEFMRHLTNEEQQQLLKYLPPVDSAKPPDSLRSMFESSQFKENLSYF QQ

Table S4. *SiGATA* gene promoter region cis-acting element details.

Supplementary Table S4 Cis-regulatory elements in the promoter region of SiGATA in this study.

Category	Cis-regulatory elements	Sequence	Function	Number of genes	Gene name
Devolepment related elements	as-1	TGACG	root-specific expression	24	SiGATA02, SiGATA03, SiGATA04, SiGATA05, SiGATA06, SiGATA07, SiGATA08, SiGATA09, SiGATA11, SiGATA12, SiGATA13, SiGATA14, SiGATA15, SiGATA16, SiGATA17, SiGATA18, SiGATA19, SiGATA20, SiGATA21, SiGATA24, SiGATA25, SiGATA26, SiGATA27, SiGATA28
Devolepment related elements	AAGAA-motif	gGTAAAGAAA	involved in endosperm-specific negative expression	18	SiGATA01, SiGATA02, SiGATA03, SiGATA04, SiGATA06, SiGATA08, SiGATA10, SiGATA12, SiGATA14, SiGATA15, SiGATA16, SiGATA17, SiGATA21, SiGATA23, SiGATA25, SiGATA26, SiGATA27, SiGATA28
Devolepment related elements	CAT-box	GCCACT	cis-acting regulatory element related to meristem expression	17	SiGATA01, SiGATA02, SiGATA03, SiGATA05, SiGATA06, SiGATA07, SiGATA10, SiGATA11, SiGATA12, SiGATA13, SiGATA14, SiGATA15, SiGATA21, SiGATA22, SiGATA26, SiGATA27, SiGATA28
Devolepment related elements	CCAAT-box	CAACGG	MYBHv1 binding site	15	SiGATA01, SiGATA02, SiGATA06, SiGATA09, SiGATA10, SiGATA11, SiGATA13, SiGATA14, SiGATA15, SiGATA18, SiGATA21, SiGATA22, SiGATA24, SiGATA26, SiGATA27
Devolepment related elements	O2-site	GATGATGTGG	cis-acting regulatory element involved in zein metabolism regulation	11	SiGATA02, SiGATA06, SiGATA07, SiGATA09, SiGATA17, SiGATA18, SiGATA19, SiGATA23, SiGATA24, SiGATA26, SiGATA28
Devolepment related elements	circadian	CAAAGATATC	cis-acting regulatory element involved in circadian control	5	SiGATA10, SiGATA18, SiGATA19, SiGATA20, SiGATA22
Devolepment related elements	dOCT	CACGGATC	meristem specific activation	5	SiGATA06, SiGATA07, SiGATA16, SiGATA17, SiGATA22

Devolepment related elements	GCN4_motif	TGAGTCA	cis-regulatory element involved in endosperm expression	4	SiGATA04, SiGATA07, SiGATA22, SiGATA25
Devolepment related elements	RY-element	CATGCATG	cis-acting regulatory element involved in seed-specific regulation	4	SiGATA02, SiGATA06, SiGATA12, SiGATA17
Devolepment related elements	OCT	CGCGGATC	meristem specific activation	2	SiGATA05, SiGATA22
Devolepment related elements	MBSI	aaaAaaC(G/C)GTTA	MYB binding site involved in flavonoid biosynthetic genes regulation	2	SiGATA20, SiGATA26
Devolepment related elements	MSA-like	(T/C)C(T/C)AACGG(T/C)(T/C)A	cis-acting element involved in cell cycle regulation	2	SiGATA09, SiGATA14
Devolepment related elements	AC-I	(T/C)C(T/C)(C/T)ACC(T/C)ACC	negative regulation of phloem expression; restricting the vascular expression to the xylem	1	SiGATA05
Devolepment related elements	HD-Zip 1	CAAT(A/T)ATTG	element involved in differentiation of the palisade mesophyll cells	1	SiGATA09
Devolepment related elements	motif I	gGTACGTGGCG	cis-acting regulatory element root specific	1	SiGATA08
Devolepment related elements	AACA_motif	TAACAAACTCCA	involved in endosperm-specific negative expression	1	SiGATA07
Environmental stress-related element	ARE	AAACCA	cis-acting regulatory element essential for the anaerobic induction	23	SiGATA01, SiGATA03, SiGATA05, SiGATA06, SiGATA07, SiGATA08, SiGATA09, SiGATA10, SiGATA11, SiGATA12, SiGATA13, SiGATA14, SiGATA15, SiGATA16,

					SiGATA17, SiGATA18, SiGATA19, SiGATA21, SiGATA22, SiGATA23, SiGATA25, SiGATA27, SiGATA28
Environmental stress-related element	LTR	CCGAAA	cis-acting element involved in low-temperature responsiveness	16	SiGATA02, SiGATA03, SiGATA07, SiGATA08, SiGATA13, SiGATA14, SiGATA16, SiGATA17, SiGATA19, SiGATA20, SiGATA21, SiGATA23, SiGATA24, SiGATA25, SiGATA26, SiGATA27
Environmental stress-related element	MBS	CAACTG	MYB binding site involved in drought-inducibility	15	SiGATA01, SiGATA02, SiGATA03, SiGATA04, SiGATA06, SiGATA07, SiGATA12, SiGATA13, SiGATA16, SiGATA17, SiGATA19, SiGATA20, SiGATA21, SiGATA22, SiGATA28
Environmental stress-related element	GC-motif	CCCCCG	enhancer-like element involved in anoxic specific inducibility	13	SiGATA03, SiGATA05, SiGATA06, SiGATA08, SiGATA10, SiGATA12, SiGATA13, SiGATA17, SiGATA20, SiGATA21, SiGATA22, SiGATA26, SiGATA27
Environmental stress-related element	W box	TTGACC	wounding and pathogen responsiveness (Binds WRKY type transcription factors)	12	SiGATA01, SiGATA02, SiGATA03, SiGATA06, SiGATA08, SiGATA16, SiGATA17, SiGATA20, SiGATA21, SiGATA23, SiGATA24, SiGATA26
Environmental stress-related element	box S	AGCCACC	wounding and pathogen responsiveness	9	SiGATA03, SiGATA06, SiGATA08, SiGATA10, SiGATA11, SiGATA16, SiGATA17, SiGATA19, SiGATA21
Environmental stress-related element	WUN-motif	AAATTTCCT	wound-responsive element	9	SiGATA01, SiGATA02, SiGATA05, SiGATA10, SiGATA12, SiGATA14, SiGATA20, SiGATA25, SiGATA28
Environmental stress-related element	TC-rich repeats	GTTTTCTTAC	cis-acting element involved in defense and stress responsiveness	6	SiGATA04, SiGATA09, SiGATA17, SiGATA19, SiGATA20, SiGATA25
Hormone responsive elements	ABRE	AACCCGG	cis-acting element involved in the abscisic acid responsiveness	25	SiGATA01, SiGATA02, SiGATA03, SiGATA04, SiGATA05, SiGATA06, SiGATA07, SiGATA09, SiGATA10, SiGATA11, SiGATA12, SiGATA13, SiGATA15, SiGATA16,

					SiGATA17, SiGATA18, SiGATA19, SiGATA20, SiGATA21, SiGATA23, SiGATA24, SiGATA25, SiGATA26, SiGATA27, SiGATA28
Hormone responsive elements	CGTCA-motif	CGTCA	cis-acting regulatory element involved in the MeJA-responsiveness	24	SiGATA02, SiGATA03, SiGATA04, SiGATA05, SiGATA06, SiGATA07, SiGATA08, SiGATA09, SiGATA11, SiGATA12, SiGATA13, SiGATA14, SiGATA15, SiGATA16, SiGATA17, SiGATA18, SiGATA19, SiGATA20, SiGATA21, SiGATA24, SiGATA25, SiGATA26, SiGATA27, SiGATA28
Hormone responsive elements	TGACG-motif	TGACG	cis-acting regulatory element involved in the MeJA-responsiveness	24	SiGATA02, SiGATA03, SiGATA04, SiGATA05, SiGATA06, SiGATA07, SiGATA08, SiGATA09, SiGATA11, SiGATA12, SiGATA13, SiGATA14, SiGATA15, SiGATA16, SiGATA17, SiGATA18, SiGATA19, SiGATA20, SiGATA21, SiGATA24, SiGATA25, SiGATA26, SiGATA27, SiGATA28
Hormone responsive elements	TATC-box	TATCCCA	cis-acting element involved in gibberellin-responsiveness	9	SiGATA04, SiGATA06, SiGATA09, SiGATA10, SiGATA11, SiGATA13, SiGATA15, SiGATA20, SiGATA26
Hormone responsive elements	ERE	ATTTTAAA	ethylene-responsive element	8	SiGATA01, SiGATA02, SiGATA05, SiGATA07, SiGATA10, SiGATA16, SiGATA20, SiGATA25
Hormone responsive elements	P-box	CCTTTTG	gibberellin-responsive element	8	SiGATA03, SiGATA06, SiGATA12, SiGATA15, SiGATA16, SiGATA17, SiGATA19, SiGATA21
Hormone responsive elements	TCA-element	CCATCTTTTT	cis-acting element involved in salicylic acid responsiveness	8	SiGATA04, SiGATA07, SiGATA08, SiGATA09, SiGATA16, SiGATA17, SiGATA20, SiGATA28
Hormone responsive elements	TGA-element	AACGAC	auxin-responsive element	6	SiGATA11, SiGATA16, SiGATA18, SiGATA19, SiGATA26, SiGATA27
Hormone responsive elements	GARE-motif	TCTGTTG	gibberellin-responsive element	4	SiGATA2, SiGATA11, SiGATA17, SiGATA18
Hormone responsive elements	AuxRR-core	GGTCCAT	cis-acting regulatory element involved in auxin responsiveness	3	SiGATA02, SiGATA10, SiGATA17

Hormone responsive elements	TGA-box	TGACGTAA	part of an auxin-responsive element	1	SiGATA14
Light responsive elements	G-box	CACGTC	cis-acting regulatory element involved in light responsiveness	23	SiGATA01, SiGATA02, SiGATA03, SiGATA04, SiGATA05, SiGATA06, SiGATA07, SiGATA09, SiGATA10, SiGATA11, SiGATA12, SiGATA13, SiGATA16, SiGATA17, SiGATA18, SiGATA19, SiGATA20, SiGATA23, SiGATA24, SiGATA25, SiGATA26, SiGATA27, SiGATA28
Light responsive elements	Box 4	ATTAAT	part of a conserved DNA module involved in light responsiveness	18	SiGATA01, SiGATA02, SiGATA04, SiGATA05, SiGATA07, SiGATA08, SiGATA09, SiGATA10, SiGATA12, SiGATA13, SiGATA15, SiGATA19, SiGATA20, SiGATA21, SiGATA24, SiGATA25, SiGATA26, SiGATA28
Light responsive elements	Sp1	GGGCGG	light responsive element	15	SiGATA03, SiGATA04, SiGATA05, SiGATA06, SiGATA10, SiGATA13, SiGATA14, SiGATA16, SiGATA17, SiGATA18, SiGATA19, SiGATA21, SiGATA22, SiGATA23, SiGATA27
Light responsive elements	TCCC-motif	TCTCCCT	part of a light responsive element	13	SiGATA07, SiGATA08, SiGATA09, SiGATA10, SiGATA12, SiGATA14, SiGATA16, SiGATA17, SiGATA19, SiGATA21, SiGATA25, SiGATA27, SiGATA28
Light responsive elements	GT1-motif	GGTTAA	light responsive element	12	SiGATA06, SiGATA07, SiGATA09, SiGATA11, SiGATA12, SiGATA14, SiGATA20, SiGATA22, SiGATA24, SiGATA25, SiGATA27, SiGATA28
Light responsive elements	GATA-motif	AAGGATAAGG	part of a light responsive element	11	SiGATA01, SiGATA08, SiGATA09, SiGATA12, SiGATA15, SiGATA18, SiGATA20, SiGATA22, SiGATA24, SiGATA26, SiGATA28
Light responsive elements	I-box	gGATAAGGTG	part of a light responsive element	10	SiGATA01, SiGATA04, SiGATA06, SiGATA10, SiGATA12, SiGATA14, SiGATA18, SiGATA19, SiGATA24, SiGATA26
Light responsive elements	TCT-motif	TCTTAC	part of a light responsive element	10	SiGATA02, SiGATA08, SiGATA11, SiGATA12, SiGATA14, SiGATA15, SiGATA17, SiGATA18, SiGATA19, SiGATA24
Light responsive elements	ACE	GACACGTATG	cis-acting element involved in light responsiveness	6	SiGATA04, SiGATA05, SiGATA06, SiGATA09, SiGATA15, SiGATA25
Light responsive elements	AE-box	AGAAACAA	part of a module for light response	6	SiGATA06, SiGATA14, SiGATA15, SiGATA20, SiGATA25, SiGATA26

Light responsive elements	ATCT-motif	AATCTAATCC	part of a conserved DNA module involved in light responsiveness	6	SiGATA05, SiGATA08, SiGATA09, SiGATA12, SiGATA13, SiGATA19
Light responsive elements	GA-motif	ATAGATAA	part of a light responsive element	4	SiGATA10, SiGATA11, SiGATA17, SiGATA25
Light responsive elements	ATC-motif	AGCTATCCA	part of a conserved DNA module involved in light responsiveness	4	SiGATA02, SiGATA05, SiGATA17, SiGATA25
Light responsive elements	MRE	AACCTAA	MYB binding site involved in light responsiveness	3	SiGATA11, SiGATA12, SiGATA25
Light responsive elements	chs-CMA1a	TTACTTAA	part of a light responsive element	3	SiGATA09, SiGATA11, SiGATA17
Light responsive elements	GTGGC-motif	CAGCGTGTGG C	part of a light responsive element	2	SiGATA12, SiGATA22
Light responsive elements	LAMP-element	CCTTATCCA	part of a light responsive element	2	SiGATA01, SiGATA02
Light responsive elements	CAG-motif	GAAAGGCAGA C	part of a light response element	2	SiGATA04, SiGATA09
Light responsive elements	Pc-CMA2c	GCCCACGCA	part of a light responsive element	1	SiGATA04
Light responsive elements	3-AF1 binding site	TAAGAGAGGA A	light responsive element	1	SiGATA17
Light responsive elements	Box II	TGGTAATAA	part of a light responsive element	1	SiGATA03
Light responsive elements	chs-CMA2a	TCACTTGA	part of a light responsive element	1	SiGATA18

Other elements	Unnamed__4	CTCC	28	SiGATA01, SiGATA02, SiGATA03, SiGATA04, SiGATA05, SiGATA06, SiGATA07, SiGATA08, SiGATA09, SiGATA10, SiGATA11, SiGATA12, SiGATA13, SiGATA14, SiGATA15, SiGATA16, SiGATA17, SiGATA18, SiGATA19, SiGATA20, SiGATA21, SiGATA22, SiGATA23, SiGATA24, SiGATA25, SiGATA26, SiGATA27, SiGATA28
Other elements	STRE	AGGGG	27	SiGATA01, SiGATA02, SiGATA03, SiGATA04, SiGATA05, SiGATA06, SiGATA07, SiGATA08, SiGATA09, SiGATA10, SiGATA11, SiGATA12, SiGATA13, SiGATA14, SiGATA15, SiGATA16, SiGATA17, SiGATA18, SiGATA19, SiGATA20, SiGATA21, SiGATA22, SiGATA24, SiGATA25, SiGATA26, SiGATA27, SiGATA28
Other elements	MYB	TAACCA	26	SiGATA01, SiGATA02, SiGATA03, SiGATA04, SiGATA05, SiGATA06, SiGATA07, SiGATA08, SiGATA09, SiGATA10, SiGATA11, SiGATA12, SiGATA13, SiGATA14, SiGATA15, SiGATA16, SiGATA17, SiGATA18, SiGATA19, SiGATA20, SiGATA21, SiGATA22, SiGATA24, SiGATA26, SiGATA27, SiGATA28
Other elements	MYC	CATTTG	24	SiGATA01, SiGATA02, SiGATA03, SiGATA04, SiGATA06, SiGATA07, SiGATA08, SiGATA09, SiGATA10, SiGATA12, SiGATA14, SiGATA15, SiGATA16, SiGATA17, SiGATA18, SiGATA20, SiGATA21, SiGATA22, SiGATA23, SiGATA24, SiGATA25, SiGATA26, SiGATA27, SiGATA28
Other elements	Myb-binding site	CAACAG	20	SiGATA01, SiGATA02, SiGATA04, SiGATA05, SiGATA07, SiGATA08, SiGATA10, SiGATA11, SiGATA12, SiGATA13, SiGATA14, SiGATA15, SiGATA17, SiGATA18, SiGATA21, SiGATA22, SiGATA24, SiGATA26, SiGATA27, SiGATA28
Other elements	WRE3	CCACCT	16	SiGATA01, SiGATA04, SiGATA05, SiGATA06, SiGATA08, SiGATA12, SiGATA13, SiGATA16, SiGATA17, SiGATA18, SiGATA19, SiGATA20, SiGATA21, SiGATA22, SiGATA25, SiGATA26
Other elements	MYB recognition site	CCGTTG	15	SiGATA01, SiGATA02, SiGATA06, SiGATA09, SiGATA10, SiGATA11, SiGATA13, SiGATA14, SiGATA15, SiGATA18, SiGATA21, SiGATA22, SiGATA24, SiGATA26, SiGATA27

Other elements	Unnamed__2	CCCCGG	15	SiGATA03, SiGATA04, SiGATA05, SiGATA06, SiGATA07, SiGATA08, SiGATA12, SiGATA13, SiGATA14, SiGATA16, SiGATA19, SiGATA20, SiGATA21, SiGATA22, SiGATA27
Other elements	MYB-like sequence	TAACCA	14	SiGATA01, SiGATA06, SiGATA09, SiGATA10, SiGATA11, SiGATA12, SiGATA15, SiGATA16, SiGATA17, SiGATA19, SiGATA20, SiGATA26, SiGATA27, SiGATA28
Other elements	CCGTCC-box	CCGTCC	14	SiGATA01, SiGATA03, SiGATA05, SiGATA06, SiGATA07, SiGATA10, SiGATA11, SiGATA12, SiGATA14, SiGATA16, SiGATA21, SiGATA22, SiGATA26, SiGATA27
Other elements	ABRE3a	TACGTG	13	SiGATA04, SiGATA05, SiGATA06, SiGATA07, SiGATA10, SiGATA11, SiGATA12, SiGATA16, SiGATA17, SiGATA23, SiGATA24, SiGATA25, SiGATA27
Other elements	ABRE4	CACGTA	13	SiGATA04, SiGATA05, SiGATA06, SiGATA07, SiGATA10, SiGATA11, SiGATA12, SiGATA16, SiGATA17, SiGATA23, SiGATA24, SiGATA25, SiGATA27
Other elements	AT~TATA-box	TATATA	13	SiGATA06, SiGATA07, SiGATA08, SiGATA09, SiGATA10, SiGATA11, SiGATA12, SiGATA13, SiGATA16, SiGATA17, SiGATA18, SiGATA20, SiGATA24
Other elements	DRE core	GCCGAC	13	SiGATA06, SiGATA07, SiGATA16, SiGATA17, SiGATA22, SiGATA01, SiGATA02, SiGATA04, SiGATA05, SiGATA06, SiGATA09, SiGATA12, SiGATA13, SiGATA16, SiGATA18, SiGATA19, SiGATA21, SiGATA26
Other elements	TCA	TCATCTTCAT	12	SiGATA01, SiGATA02, SiGATA10, SiGATA12, SiGATA13, SiGATA14, SiGATA16, SiGATA17, SiGATA18, SiGATA20, SiGATA22, SiGATA28
Other elements	CTAG-motif	ACTAGCAGAA	8	SiGATA03, SiGATA06, SiGATA07, SiGATA08, SiGATA10, SiGATA19, SiGATA22, SiGATA23
Other elements	TATA	TATAAAAT	6	SiGATA01, SiGATA05, SiGATA10, SiGATA11, SiGATA20, SiGATA24
Other elements	DRE1	ACCGAGA	5	SiGATA09, SiGATA13, SiGATA19, SiGATA26, SiGATA27
Other elements	CARE	CAACTCCC	4	SiGATA08, SiGATA11, SiGATA12, SiGATA19
Other elements	F-box	CTATTCTCATT	3	SiGATA07, SiGATA18, SiGATA28
Other elements	Unnamed__16	GCTGCCCGTC	3	SiGATA06, SiGATA17, SiGATA21
Other elements	AP-1	TGAGTTAG	2	SiGATA05, SiGATA26

Other elements	AT~ABRE	TACGTGTC		2	SiGATA05, SiGATA23
Other elements	re2f-1	GCGGGAAA		2	SiGATA16, SiGATA22
Other elements	NON	CAACGGCCACG		2	SiGATA06, SiGATA15
Other elements	AT-rich sequence	TAAAATACT	element for maximal elicitor-mediated activation (2copies)	1	SiGATA16
Promoter related element	CAAT-box	CAAT		28	SiGATA01, SiGATA02, SiGATA03, SiGATA04, SiGATA05, SiGATA06, SiGATA07, SiGATA08, SiGATA09, SiGATA10, SiGATA11, SiGATA12, SiGATA13, SiGATA14, SiGATA15, SiGATA16, SiGATA17, SiGATA18, SiGATA19, SiGATA20, SiGATA21, SiGATA22, SiGATA23, SiGATA24, SiGATA25, SiGATA26, SiGATA27, SiGATA28
Promoter related element	TATA-box	TATACA	core promoter element around -30 of transcription start	28	SiGATA01, SiGATA02, SiGATA03, SiGATA04, SiGATA05, SiGATA06, SiGATA07, SiGATA08, SiGATA09, SiGATA10, SiGATA11, SiGATA12, SiGATA13, SiGATA14, SiGATA15, SiGATA16, SiGATA17, SiGATA18, SiGATA19, SiGATA20, SiGATA21, SiGATA22, SiGATA23, SiGATA24, SiGATA25, SiGATA26, SiGATA27, SiGATA28
Promoter related element	A-box	CCGTCC	cis-acting regulatory element	14	SiGATA01, SiGATA03, SiGATA05, SiGATA06, SiGATA07, SiGATA10, SiGATA11, SiGATA12, SiGATA14, SiGATA16, SiGATA21, SiGATA22, SiGATA26, SiGATA27
Promoter related element	CCGTCC motif	CCGTCC		14	SiGATA01, SiGATA03, SiGATA05, SiGATA06, SiGATA07, SiGATA10, SiGATA11, SiGATA12, SiGATA14, SiGATA16, SiGATA21, SiGATA22, SiGATA26, SiGATA27
Site-binding related elements	Unnamed__1	CGTGG		22	SiGATA02, SiGATA03, SiGATA04, SiGATA05, SiGATA06, SiGATA07, SiGATA08, SiGATA09, SiGATA10, SiGATA11, SiGATA12, SiGATA14, SiGATA16, SiGATA17, SiGATA18, SiGATA20, SiGATA21, SiGATA24, SiGATA25, SiGATA26, SiGATA27, SiGATA28
Site-binding related elements	E2Fb	TTTGCCGC		2	SiGATA06, SiGATA27
Site-binding related elements	AT-rich element	ATAGAAATCAA	binding site of AT-rich DNA binding protein (ATBP-1)	1	SiGATA13

Site-binding related elements	Unnamed__6	taTAAATATct	1	SiGATA19

Table S5. Tandem duplication and fragment duplication events of the *S. italica* GATA genes.

Supplementary Table S5-1 The 3 pairs of tandem duplicates in Setaria italica GATA genes.

Group	Transcript	Gene ID	Gene	Chr Location	Start	End		Group	Transcript	Gene ID	Gene	Chr Location	Start	End
Subfamily I	transcript:KQK88841	SETIT_039011mg	SiGATA25	IX	17616653	17617999	==	Subfamily I	transcript:KQK88842	SETIT_034871mg	SiGATA26	IX	17646370	17648595
Subfamily IV	transcript:KQK97872	SETIT_010863mg	SiGATA14	VII	23668062	23671360	==	Subfamily IV	transcript:KQK97873	SETIT_011929mg	SiGATA15	VII	23690759	23692758
Subfamily I	transcript:KQK99539	SETIT_010259mg	SiGATA18	VII	33403483	33404759	==	Subfamily I	transcript:KQK99540	SETIT_010130mg	SiGATA19	VII	33411756	33413570

Supplementary Table S5-2 The 5 pairs of segmental duplicates in Setaria italica GATA genes.

Group	Gene ID	Gene	Transcript	Chr Location		Group	Gene ID	Gene	Transcript	Chr Location
Subfamily I	SETIT_017219mg	SiGATA03	transcript:KQL30710	I	==	Subfamily I	SETIT_010126mg	SiGATA16	transcript:KQK98295	VII
Subfamily II	SETIT_004417mg	SiGATA11	transcript:KQL05071	V	==	Subfamily II	SETIT_011269mg	SiGATA17	transcript:KQK99264	VII
Subfamily I	SETIT_022327mg	SiGATA07	transcript:KQL14695	III	==	Subfamily I	SETIT_001735mg	SiGATA12	transcript:KQL06949	V
Subfamily II	SETIT_019058mg	SiGATA01	transcript:KQL28093	I	==	Subfamily II	SETIT_006733mg	SiGATA09	transcript:KQL11659	IV
Subfamily II	SETIT_022366mg	SiGATA05	transcript:KQL14176	III	==	Subfamily II	SETIT_024214mg	SiGATA06	transcript:KQL14269	III

Table S6. One-to-one orthologous GATA gene relationships between *S. italica* and other plants.

Supplementary Table S6-1 One-to-one orthologous relationships between *Setaria italica* and *Arabidopsis thaliana*.

Group	SiGATA Gene Name	SiGATA Gene ID	Transcript	Chr Postion		At Gene ID	Chr Postion
Subfamily II	SiGATA11	SETIT_004417mg	transcript:KQL05071	V	==	AT3G06740.1	3
Subfamily II	SiGATA11	SETIT_004417mg	transcript:KQL05071	V	==	AT3G16870.1	3
Subfamily II	SiGATA17	SETIT_011269mg	transcript:KQK99264	VII	==	AT3G16870.1	3
Subfamily I	SiGATA27	SETIT_036273mg	transcript:KQK89954	IX	==	AT2G45050.1	2

Supplementary Table S6-2 One-to-one orthologous relationships between *Setaria italica* and *Solanum lycopersicum*.

Group	SiGATA Gene Name	SiGATA Gene ID	Transcript	Chr Postion		Sl Gene ID	Chr Postion
Subfamily I	SiGATA07	SETIT_022327mg	transcript:KQL14695	III	==	Solyc03g120890.3.1	3
Subfamily I	SiGATA07	SETIT_022327mg	transcript:KQL14695	III	==	Solyc08g066510.3.1	8
Subfamily II	SiGATA11	SETIT_004417mg	transcript:KQL05071	V	==	Solyc01g100220.3.1	1
Subfamily II	SiGATA11	SETIT_004417mg	transcript:KQL05071	V	==	Solyc12g099370.2.1	12
Subfamily I	SiGATA12	SETIT_001735mg	transcript:KQL06949	V	==	Solyc08g066510.3.1	8
Subfamily II	SiGATA17	SETIT_011269mg	transcript:KQK99264	VII	==	Solyc12g099370.2.1	12
Subfamily I	SiGATA27	SETIT_036273mg	transcript:KQK89954	IX	==	Solyc01g090760.3.1	1

Supplementary Table S6-3 One-to-one orthologous relationships between *Setaria italica* and *Glycine max*.

Group	SiGATA Gene Name	SiGATA Gene ID	Transcript	Chr Postion		Gm Gene ID	Chr Postion
Subfamily I	SiGATA07	SETIT_022327mg	transcript:KQL14695	III	==	GLYMA_04G084900	4
Subfamily I	SiGATA07	SETIT_022327mg	transcript:KQL14695	III	==	GLYMA_06G086400	6
Subfamily II	SiGATA11	SETIT_004417mg	transcript:KQL05071	V	==	GLYMA_17G030900	17
Subfamily II	SiGATA11	SETIT_004417mg	transcript:KQL05071	V	==	GLYMA_03G232900	3
Subfamily I	SiGATA12	SETIT_001735mg	transcript:KQL06949	V	==	GLYMA_04G084900	4
Subfamily I	SiGATA12	SETIT_001735mg	transcript:KQL06949	V	==	GLYMA_06G086400	6
Subfamily I	SiGATA12	SETIT_001735mg	transcript:KQL06949	V	==	GLYMA_07G016800	7
Subfamily I	SiGATA12	SETIT_001735mg	transcript:KQL06949	V	==	GLYMA_08G202100	8
Subfamily II	SiGATA17	SETIT_011269mg	transcript:KQK99264	VII	==	GLYMA_19G229900	19
Subfamily II	SiGATA17	SETIT_011269mg	transcript:KQK99264	VII	==	GLYMA_03G232900	3
Subfamily II	SiGATA22	SETIT_037892mg	transcript:KQK86338	IX	==	GLYMA_17G030900	17

Supplementary Table S6-4 One-to-one orthologous relationships between *Setaria italica* and *Oryza sativa*.

Group	SiGATA Gene Name	SiGATA Gene ID	Transcript	Chr Postion		Os Gene ID	Chr Postion
Subfamily III	SiGATA23	SETIT_036747mg	transcript:KQK87242	IX	==	LOC_Os03g52450.1	Chr3
Subfamily III	SiGATA24	SETIT_036492mg	transcript:KQK87704	IX	==	LOC_Os03g47970.1	Chr3
Subfamily I	SiGATA25	SETIT_039011mg	transcript:KQK88841	IX	==	LOC_Os10g32070.1	Chr10
Subfamily I	SiGATA25	SETIT_039011mg	transcript:KQK88841	IX	==	LOC_Os03g05160.1	Chr3
Subfamily I	SiGATA27	SETIT_036273mg	transcript:KQK89954	IX	==	LOC_Os10g40810.1	Chr10
Subfamily I	SiGATA16	SETIT_010126mg	transcript:KQK98295	VII	==	LOC_Os02g43150.1	Chr2
Subfamily I	SiGATA16	SETIT_010126mg	transcript:KQK98295	VII	==	LOC_Os04g45650.2	Chr4
Subfamily II	SiGATA17	SETIT_011269mg	transcript:KQK99264	VII	==	LOC_Os05g06340.1	Chr5

Subfamily I	SiGATA18	SETIT_010259mg	transcript:KQK99539	VII	==	LOC_Os12g07120.1	Chr12
Subfamily II	SiGATA11	SETIT_004417mg	transcript:KQL05071	V	==	LOC_Os05g06340.1	Chr5
Subfamily I	SiGATA12	SETIT_001735mg	transcript:KQL06949	V	==	LOC_Os01g54210.1	Chr1
Subfamily I	SiGATA12	SETIT_001735mg	transcript:KQL06949	V	==	LOC_Os05g44400.1	Chr5
Subfamily II	SiGATA13	SETIT_002983mg	transcript:KQL08815	V	==	LOC_Os01g74540.1	Chr1
Subfamily II	SiGATA09	SETIT_006733mg	transcript:KQL11659	IV	==	LOC_Os02g12790.1	Chr2
Subfamily II	SiGATA09	SETIT_006733mg	transcript:KQL11659	IV	==	LOC_Os06g37450.1	Chr6
Subfamily III	SiGATA10	SETIT_006750mg	transcript:KQL11783	IV	==	LOC_Os02g05510.1	Chr2
Subfamily III	SiGATA10	SETIT_006750mg	transcript:KQL11783	IV	==	LOC_Os06g48534.1	Chr6
Subfamily II	SiGATA05	SETIT_022366mg	transcript:KQL14176	III	==	LOC_Os01g47360.1	Chr1
Subfamily II	SiGATA05	SETIT_022366mg	transcript:KQL14176	III	==	LOC_Os05g49280.1	Chr5
Subfamily II	SiGATA05	SETIT_022366mg	transcript:KQL14176	III	==	LOC_Os05g50270.1	Chr5
Subfamily II	SiGATA06	SETIT_024214mg	transcript:KQL14269	III	==	LOC_Os01g47360.1	Chr1
Subfamily II	SiGATA06	SETIT_024214mg	transcript:KQL14269	III	==	LOC_Os05g50270.1	Chr5
Subfamily II	SiGATA06	SETIT_024214mg	transcript:KQL14269	III	==	LOC_Os05g49280.1	Chr5
Subfamily I	SiGATA07	SETIT_022327mg	transcript:KQL14695	III	==	LOC_Os01g54210.1	Chr1
Subfamily I	SiGATA07	SETIT_022327mg	transcript:KQL14695	III	==	LOC_Os05g44400.1	Chr5
Subfamily I	SiGATA08	SETIT_022727mg	transcript:KQL17328	III	==	LOC_Os12g42970.1	Chr12
Subfamily II	SiGATA01	SETIT_019058mg	transcript:KQL28093	I	==	LOC_Os02g12790.1	Chr2
Subfamily II	SiGATA01	SETIT_019058mg	transcript:KQL28093	I	==	LOC_Os06g37450.1	Chr6
Subfamily I	SiGATA03	SETIT_017219mg	transcript:KQL30710	I	==	LOC_Os02g43150.1	Chr2
Subfamily I	SiGATA03	SETIT_017219mg	transcript:KQL30710	I	==	LOC_Os04g45650.2	Chr4
Subfamily I	SiGATA04	SETIT_017317mg	transcript:KQL32016	I	==	LOC_Os02g56250.1	Chr2

Supplementary Table S6-5 One-to-one orthologous relationships between *Setaria italica* and *Brachypodium distachyon*.

Group	SiGATA Gene Name	SiGATA Gene ID	Transcript	Chr Postion		Bd Gene ID	Chr Postion
Subfamily I	SiGATA16	SETIT_010126mg	transcript:KQK98295	VII	==	BRADI_5g17057v3	5
Subfamily I	SiGATA03	SETIT_017219mg	transcript:KQL30710	I	==	BRADI_5g17057v3	5
Subfamily I	SiGATA08	SETIT_022727mg	transcript:KQL17328	III	==	BRADI_4g01140v3	4
Subfamily III	SiGATA21	SETIT_027518mg	transcript:KQK95022	VIII	==	BRADI_4g15720v3	4
Subfamily I	SiGATA20	SETIT_028169mg	transcript:KQK93911	VIII	==	BRADI_4g23550v3	4
Subfamily I	SiGATA18	SETIT_010259mg	transcript:KQK99539	VII	==	BRADI_4g41570v3	4
Subfamily III	SiGATA10	SETIT_006750mg	transcript:KQL11783	IV	==	BRADI_3g03800v3	3
Subfamily II	SiGATA09	SETIT_006733mg	transcript:KQL11659	IV	==	BRADI_3g08240v3	3
Subfamily II	SiGATA01	SETIT_019058mg	transcript:KQL28093	I	==	BRADI_3g08240v3	3
Subfamily I	SiGATA25	SETIT_039011mg	transcript:KQK88841	IX	==	BRADI_3g28003v3	3
Subfamily I	SiGATA27	SETIT_036273mg	transcript:KQK89954	IX	==	BRADI_3g33200v3	3
Subfamily I	SiGATA16	SETIT_010126mg	transcript:KQK98295	VII	==	BRADI_3g50160v3	3
Subfamily I	SiGATA03	SETIT_017219mg	transcript:KQL30710	I	==	BRADI_3g50160v3	3
Subfamily I	SiGATA04	SETIT_017317mg	transcript:KQL32016	I	==	BRADI_3g54720v3	3
Subfamily II	SiGATA17	SETIT_011269mg	transcript:KQK99264	VII	==	BRADI_2g12590v3	2
Subfamily II	SiGATA11	SETIT_004417mg	transcript:KQL05071	V	==	BRADI_2g12590v3	2
Subfamily II	SiGATA05	SETIT_022366mg	transcript:KQL14176	III	==	BRADI_2g14890v3	2
Subfamily II	SiGATA06	SETIT_024214mg	transcript:KQL14269	III	==	BRADI_2g14890v3	2
Subfamily I	SiGATA12	SETIT_001735mg	transcript:KQL06949	V	==	BRADI_2g19480v3	2
Subfamily I	SiGATA07	SETIT_022327mg	transcript:KQL14695	III	==	BRADI_2g19480v3	2
Subfamily II	SiGATA17	SETIT_011269mg	transcript:KQK99264	VII	==	BRADI_2g35057v3	2
Subfamily II	SiGATA11	SETIT_004417mg	transcript:KQL05071	V	==	BRADI_2g35057v3	2
Subfamily II	SiGATA05	SETIT_022366mg	transcript:KQL14176	III	==	BRADI_2g45750v3	2
Subfamily II	SiGATA06	SETIT_024214mg	transcript:KQL14269	III	==	BRADI_2g45750v3	2

Subfamily I	SiGATA12	SETIT_001735mg	transcript:KQL06949	V	==	BRADI_2g49620v3	2
Subfamily I	SiGATA07	SETIT_022327mg	transcript:KQL14695	III	==	BRADI_2g49620v3	2
Subfamily II	SiGATA13	SETIT_002983mg	transcript:KQL08815	V	==	BRADI_2g62660v3	2
Subfamily II	SiGATA22	SETIT_037892mg	transcript:KQK86338	IX	==	BRADI_1g03020v3	1
Subfamily III	SiGATA23	SETIT_036747mg	transcript:KQK87242	IX	==	BRADI_1g09550v3	1
Subfamily III	SiGATA24	SETIT_036492mg	transcript:KQK87704	IX	==	BRADI_1g12330v3	1
Subfamily III	SiGATA10	SETIT_006750mg	transcript:KQL11783	IV	==	BRADI_1g33980v3	1
Subfamily II	SiGATA09	SETIT_006733mg	transcript:KQL11659	IV	==	BRADI_1g37480v3	1
Subfamily II	SiGATA01	SETIT_019058mg	transcript:KQL28093	I	==	BRADI_1g37480v3	1
Subfamily I	SiGATA25	SETIT_039011mg	transcript:KQK88841	IX	==	BRADI_1g75420v3	1
Subfamily I	SiGATA27	SETIT_036273mg	transcript:KQK89954	IX	==	BRADI_1g78540v3	1

Supplementary Table S6-6 One-to-one orthologous relationships between *Setaria italica* and *Zea mays*.

Group	SiGATA Gene Name	SiGATA Gene ID	Transcript	Chr Postion		Zm Gene ID	Chr Postion
Subfamily II	SiGATA22	SETIT_037892mg	transcript:KQK86338	IX	==	Zm00001d034751_T001	1
Subfamily III	SiGATA23	SETIT_036747mg	transcript:KQK87242	IX	==	Zm00001d013331_T001	5
Subfamily III	SiGATA24	SETIT_036492mg	transcript:KQK87704	IX	==	Zm00001d033523_T001	1
Subfamily I	SiGATA25	SETIT_039011mg	transcript:KQK88841	IX	==	Zm00001d048391_T001	9
Subfamily I	SiGATA27	SETIT_036273mg	transcript:KQK89954	IX	==	Zm00001d029896_T001	1
Subfamily I	SiGATA27	SETIT_036273mg	transcript:KQK89954	IX	==	Zm00001d047081_T001	9
Subfamily I	SiGATA28	SETIT_040064mg	transcript:KQK89964	IX	==	Zm00001d029896_T001	1
Subfamily I	SiGATA16	SETIT_010126mg	transcript:KQK98295	VII	==	Zm00001d025953_T003	10
Subfamily I	SiGATA16	SETIT_010126mg	transcript:KQK98295	VII	==	Zm00001d002811_T002	2
Subfamily I	SiGATA16	SETIT_010126mg	transcript:KQK98295	VII	==	Zm00001d017409_T001	5
Subfamily II	SiGATA17	SETIT_011269mg	transcript:KQK99264	VII	==	Zm00001d040775_T001	3

Subfamily II	SiGATA17	SETIT_011269mg	transcript:KQK99264	VII	==	Zm00001d037605_T001	6
Subfamily II	SiGATA17	SETIT_011269mg	transcript:KQK99264	VII	==	Zm00001d009193_T001	8
Subfamily I	SiGATA18	SETIT_010259mg	transcript:KQK99539	VII	==	Zm00001d023539_T001	10
Subfamily I	SiGATA18	SETIT_010259mg	transcript:KQK99539	VII	==	Zm00001d041883_T001	3
Subfamily II	SiGATA11	SETIT_004417mg	transcript:KQL05071	V	==	Zm00001d040775_T001	3
Subfamily II	SiGATA11	SETIT_004417mg	transcript:KQL05071	V	==	Zm00001d009193_T001	8
Subfamily I	SiGATA12	SETIT_001735mg	transcript:KQL06949	V	==	Zm00001d038801_T001	6
Subfamily I	SiGATA12	SETIT_001735mg	transcript:KQL06949	V	==	Zm00001d012757_T001	8
Subfamily I	SiGATA12	SETIT_001735mg	transcript:KQL06949	V	==	Zm00001d010785_T001	8
Subfamily II	SiGATA13	SETIT_002983mg	transcript:KQL08815	V	==	Zm00001d011771_T001	8
Subfamily II	SiGATA09	SETIT_006733mg	transcript:KQL11659	IV	==	Zm00001d016361_T001	5
Subfamily II	SiGATA09	SETIT_006733mg	transcript:KQL11659	IV	==	Zm00001d046354_T001	9
Subfamily III	SiGATA10	SETIT_006750mg	transcript:KQL11783	IV	==	Zm00001d014656_T003	5
Subfamily III	SiGATA10	SETIT_006750mg	transcript:KQL11783	IV	==	Zm00001d036494_T001	6
Subfamily II	SiGATA05	SETIT_022366mg	transcript:KQL14176	III	==	Zm00001d043969_T001	3
Subfamily II	SiGATA05	SETIT_022366mg	transcript:KQL14176	III	==	Zm00001d039113_T001	6
Subfamily II	SiGATA05	SETIT_022366mg	transcript:KQL14176	III	==	Zm00001d009668_T001	8
Subfamily II	SiGATA06	SETIT_024214mg	transcript:KQL14269	III	==	Zm00001d043969_T001	3
Subfamily II	SiGATA06	SETIT_024214mg	transcript:KQL14269	III	==	Zm00001d039113_T001	6
Subfamily II	SiGATA06	SETIT_024214mg	transcript:KQL14269	III	==	Zm00001d009604_T001	8
Subfamily I	SiGATA07	SETIT_022327mg	transcript:KQL14695	III	==	Zm00001d038801_T001	6
Subfamily I	SiGATA07	SETIT_022327mg	transcript:KQL14695	III	==	Zm00001d012757_T001	8
Subfamily I	SiGATA07	SETIT_022327mg	transcript:KQL14695	III	==	Zm00001d010785_T001	8
Subfamily I	SiGATA08	SETIT_022727mg	transcript:KQL17328	III	==	Zm00001d031135_T001	1
Subfamily II	SiGATA01	SETIT_019058mg	transcript:KQL28093	I	==	Zm00001d016361_T001	5
Subfamily II	SiGATA01	SETIT_019058mg	transcript:KQL28093	I	==	Zm00001d046354_T001	9

Subfamily I	SiGATA03	SETIT_017219mg	transcript:KQL30710	I	==	Zm00001d025953_T003	10
Subfamily I	SiGATA03	SETIT_017219mg	transcript:KQL30710	I	==	Zm00001d002811_T002	2
Subfamily I	SiGATA03	SETIT_017219mg	transcript:KQL30710	I	==	Zm00001d017409_T001	5
Subfamily I	SiGATA04	SETIT_017317mg	transcript:KQL32016	I	==	Zm00001d051981_T001	4
Subfamily I	SiGATA04	SETIT_017317mg	transcript:KQL32016	I	==	Zm00001d018421_T001	5

Table S7. Ka/ks values of each subfamily gene pair and all duplication events gene pairs.

Additional file7 Table S7-1. Ka/Ks ratio of *SiGATA* genes in Subfamily I-IV.

Clade	Gene 1	Gene 2	Ka	Ks	Ka/Ks	EffectiveLen	AverageS-sites	AverageN-sites	cN	cS	pN	pS	Note
Subfamily I	SiGATA02	SiGATA03	0.83	1.02	0.82	903.00	234.08	668.92	336.42	130.58	0.50	0.56	
Subfamily I	SiGATA02	SiGATA04	0.83	1.06	0.78	876.00	223.25	652.75	327.50	126.50	0.50	0.57	
Subfamily I	SiGATA02	SiGATA07	0.95	1.42	0.67	888.00	229.58	658.42	354.75	146.25	0.54	0.64	
Subfamily I	SiGATA02	SiGATA08	0.91	NaN	NaN	795.00	191.92	603.08	318.25	149.75	0.53	0.78	High Sequence Divergence Value (pS>=0.75)
Subfamily I	SiGATA02	SiGATA12	0.75	1.48	0.50	873.00	223.75	649.25	307.42	144.58	0.47	0.65	
Subfamily I	SiGATA02	SiGATA16	0.78	1.13	0.69	891.00	227.58	663.42	321.25	132.75	0.48	0.58	
Subfamily I	SiGATA02	SiGATA18	1.18	1.07	1.10	927.00	236.00	691.00	410.42	134.58	0.59	0.57	
Subfamily I	SiGATA02	SiGATA19	0.89	1.08	0.82	885.00	228.00	657.00	341.50	130.50	0.52	0.57	
Subfamily I	SiGATA02	SiGATA20	0.45	0.83	0.54	861.00	221.17	639.83	214.92	111.08	0.34	0.50	
Subfamily I	SiGATA02	SiGATA25	0.67	1.13	0.59	636.00	163.00	473.00	209.00	95.00	0.44	0.58	
Subfamily I	SiGATA02	SiGATA26	0.76	1.09	0.70	900.00	237.58	662.42	317.50	136.50	0.48	0.57	
Subfamily I	SiGATA02	SiGATA27	0.89	1.54	0.58	900.00	227.92	672.08	351.00	149.00	0.52	0.65	
Subfamily I	SiGATA02	SiGATA28	1.02	1.38	0.74	834.00	211.67	622.33	346.42	133.58	0.56	0.63	
Subfamily I	SiGATA03	SiGATA04	0.88	0.77	1.14	1176.00	305.75	870.25	451.58	147.42	0.52	0.48	
Subfamily I	SiGATA03	SiGATA07	0.59	0.85	0.69	996.00	265.75	730.25	297.08	134.92	0.41	0.51	
Subfamily I	SiGATA03	SiGATA08	0.89	2.52	0.35	912.00	224.33	687.67	357.58	162.42	0.52	0.72	
Subfamily I	SiGATA03	SiGATA12	0.59	0.93	0.63	1026.00	277.17	748.83	306.17	147.83	0.41	0.53	
Subfamily I	SiGATA03	SiGATA16	0.25	0.53	0.47	1242.00	331.08	910.92	193.08	125.92	0.21	0.38	

Subfamily I	SiGATA03	SiGATA18	0.88	1.09	0.81	957.00	256.33	700.67	362.75	147.25	0.52	0.57
Subfamily I	SiGATA03	SiGATA19	0.97	1.00	0.96	996.00	271.92	724.08	393.50	150.50	0.54	0.55
Subfamily I	SiGATA03	SiGATA20	0.88	1.22	0.72	1131.00	300.83	830.17	429.00	181.00	0.52	0.60
Subfamily I	SiGATA03	SiGATA25	0.58	1.08	0.54	642.00	170.08	471.92	190.75	97.25	0.40	0.57
Subfamily I	SiGATA03	SiGATA26	0.78	1.02	0.76	1230.00	338.75	891.25	431.33	188.67	0.48	0.56
Subfamily I	SiGATA03	SiGATA27	0.68	0.99	0.69	1023.00	269.08	753.92	338.42	147.58	0.45	0.55
Subfamily I	SiGATA03	SiGATA28	0.77	1.09	0.71	885.00	237.67	647.33	312.17	136.83	0.48	0.58
Subfamily I	SiGATA04	SiGATA07	0.55	0.65	0.84	975.00	254.67	720.33	279.92	111.08	0.39	0.44
Subfamily I	SiGATA04	SiGATA08	0.85	1.98	0.43	915.00	219.83	695.17	353.92	153.08	0.51	0.70
Subfamily I	SiGATA04	SiGATA12	0.66	0.81	0.81	1029.00	264.92	764.08	336.42	131.58	0.44	0.50
Subfamily I	SiGATA04	SiGATA16	0.77	0.78	1.00	1146.00	298.00	848.00	409.00	144.00	0.48	0.48
Subfamily I	SiGATA04	SiGATA18	0.91	0.91	1.00	945.00	244.67	700.33	369.00	129.00	0.53	0.53
Subfamily I	SiGATA04	SiGATA19	0.86	0.99	0.88	1005.00	259.83	745.17	382.42	142.58	0.51	0.55
Subfamily I	SiGATA04	SiGATA20	1.00	1.10	0.91	1167.00	297.92	869.08	480.17	171.83	0.55	0.58
Subfamily I	SiGATA04	SiGATA25	0.62	0.75	0.82	630.00	161.67	468.33	198.08	76.92	0.42	0.48
Subfamily I	SiGATA04	SiGATA26	0.83	1.25	0.67	1230.00	325.58	904.42	454.08	197.92	0.50	0.61
Subfamily I	SiGATA04	SiGATA27	0.73	0.82	0.88	996.00	251.75	744.25	346.25	125.75	0.47	0.50
Subfamily I	SiGATA04	SiGATA28	0.64	0.89	0.72	867.00	222.67	644.33	278.75	116.25	0.43	0.52
Subfamily I	SiGATA07	SiGATA08	0.82	2.39	0.35	864.00	213.83	650.17	325.25	153.75	0.50	0.72
Subfamily I	SiGATA07	SiGATA12	0.20	0.62	0.32	1101.00	287.33	813.67	142.17	120.83	0.17	0.42
Subfamily I	SiGATA07	SiGATA16	0.60	0.82	0.73	930.00	249.67	680.33	281.25	124.75	0.41	0.50
Subfamily I	SiGATA07	SiGATA18	0.86	0.80	1.07	1032.00	272.83	759.17	388.67	134.33	0.51	0.49
Subfamily I	SiGATA07	SiGATA19	0.68	0.76	0.90	984.00	267.00	717.00	321.67	127.33	0.45	0.48
Subfamily I	SiGATA07	SiGATA20	0.87	1.42	0.61	975.00	258.75	716.25	368.08	164.92	0.51	0.64
Subfamily I	SiGATA07	SiGATA25	0.70	0.76	0.92	738.00	188.33	549.67	250.83	90.17	0.46	0.48
Subfamily I	SiGATA07	SiGATA26	0.68	0.94	0.72	1002.00	267.67	734.33	327.42	143.58	0.45	0.54

Subfamily I	SiGATA07	SiGATA27	0.57	0.67	0.85	954.00	250.17	703.83	279.67	110.33	0.40	0.44	
Subfamily I	SiGATA07	SiGATA28	0.23	0.28	0.82	954.00	249.08	704.92	139.17	57.83	0.20	0.23	
Subfamily I	SiGATA08	SiGATA12	0.95	2.06	0.46	903.00	220.67	682.33	367.17	154.83	0.54	0.70	
Subfamily I	SiGATA08	SiGATA16	0.77	2.77	0.28	897.00	217.67	679.33	325.83	159.17	0.48	0.73	
Subfamily I	SiGATA08	SiGATA18	0.85	NaN	NaN	765.00	185.75	579.25	294.42	139.58	0.51	0.75	High Sequence Divergence Value (pS>=0.75)
Subfamily I	SiGATA08	SiGATA19	0.79	NaN	NaN	807.00	197.17	609.83	297.42	151.58	0.49	0.77	High Sequence Divergence Value (pS>=0.75)
Subfamily I	SiGATA08	SiGATA20	0.98	3.11	0.32	909.00	222.08	686.92	376.08	163.92	0.55	0.74	
Subfamily I	SiGATA08	SiGATA25	1.07	2.51	0.43	798.00	192.67	605.33	344.58	139.42	0.57	0.72	
Subfamily I	SiGATA08	SiGATA26	0.74	4.64	0.16	900.00	217.33	682.67	321.33	162.67	0.47	0.75	
Subfamily I	SiGATA08	SiGATA27	0.94	3.76	0.25	873.00	208.50	664.50	355.67	155.33	0.54	0.75	
Subfamily I	SiGATA08	SiGATA28	0.89	1.57	0.57	771.00	188.75	582.25	302.92	124.08	0.52	0.66	
Subfamily I	SiGATA12	SiGATA16	0.72	0.91	0.79	1059.00	281.67	777.33	358.58	148.42	0.46	0.53	
Subfamily I	SiGATA12	SiGATA18	0.85	0.76	1.12	1020.00	266.58	753.42	384.33	127.67	0.51	0.48	
Subfamily I	SiGATA12	SiGATA19	0.72	0.76	0.94	999.00	262.67	736.33	340.33	125.67	0.46	0.48	
Subfamily I	SiGATA12	SiGATA20	0.85	1.25	0.68	1017.00	264.00	753.00	382.58	160.42	0.51	0.61	
Subfamily I	SiGATA12	SiGATA25	0.64	0.87	0.74	717.00	184.42	532.58	230.00	95.00	0.43	0.52	
Subfamily I	SiGATA12	SiGATA26	0.67	1.00	0.68	1011.00	276.17	734.83	326.75	152.25	0.44	0.55	
Subfamily I	SiGATA12	SiGATA27	0.52	0.77	0.68	972.00	252.83	719.17	269.75	121.25	0.38	0.48	
Subfamily I	SiGATA12	SiGATA28	0.34	0.77	0.44	957.00	248.42	708.58	195.25	119.75	0.28	0.48	
Subfamily I	SiGATA16	SiGATA18	0.89	0.94	0.95	945.00	250.00	695.00	361.25	133.75	0.52	0.53	
Subfamily I	SiGATA16	SiGATA19	0.95	0.86	1.10	1008.00	272.92	735.08	396.08	139.92	0.54	0.51	
Subfamily I	SiGATA16	SiGATA20	0.99	1.31	0.76	1176.00	310.25	865.75	476.75	192.25	0.55	0.62	

Subfamily I	SiGATA16	SiGATA25	0.59	0.93	0.63	621.00	156.25	464.75	189.67	83.33	0.41	0.53
Subfamily I	SiGATA16	SiGATA26	0.88	1.14	0.77	1269.00	343.50	925.50	478.67	201.33	0.52	0.59
Subfamily I	SiGATA16	SiGATA27	0.62	0.98	0.63	1002.00	261.00	741.00	312.25	142.75	0.42	0.55
Subfamily I	SiGATA16	SiGATA28	0.72	0.92	0.78	840.00	221.83	618.17	286.17	117.83	0.46	0.53
Subfamily I	SiGATA18	SiGATA19	0.43	0.44	0.97	1026.00	271.92	754.08	245.42	90.58	0.33	0.33
Subfamily I	SiGATA18	SiGATA20	0.87	1.15	0.76	912.00	239.58	672.42	347.00	141.00	0.52	0.59
Subfamily I	SiGATA18	SiGATA25	0.83	0.95	0.86	846.00	218.67	627.33	313.92	118.08	0.50	0.54
Subfamily I	SiGATA18	SiGATA26	0.87	1.17	0.74	1143.00	301.25	841.75	432.58	178.42	0.51	0.59
Subfamily I	SiGATA18	SiGATA27	0.90	0.97	0.93	966.00	255.00	711.00	373.17	138.83	0.52	0.54
Subfamily I	SiGATA18	SiGATA28	0.82	1.03	0.80	900.00	232.92	667.08	333.75	130.25	0.50	0.56
Subfamily I	SiGATA19	SiGATA20	0.81	1.27	0.64	984.00	261.58	722.42	358.75	160.25	0.50	0.61
Subfamily I	SiGATA19	SiGATA25	0.87	1.07	0.81	849.00	221.17	627.83	323.75	126.25	0.52	0.57
Subfamily I	SiGATA19	SiGATA26	0.86	1.05	0.82	1236.00	332.00	904.00	462.17	187.83	0.51	0.57
Subfamily I	SiGATA19	SiGATA27	0.97	0.78	1.25	1023.00	268.75	754.25	410.92	130.08	0.54	0.48
Subfamily I	SiGATA19	SiGATA28	0.69	0.95	0.73	879.00	231.33	647.67	292.25	124.75	0.45	0.54
Subfamily I	SiGATA20	SiGATA25	0.63	1.19	0.53	612.00	157.75	454.25	192.92	94.08	0.42	0.60
Subfamily I	SiGATA20	SiGATA26	0.86	1.67	0.52	1239.00	330.25	908.75	465.92	221.08	0.51	0.67
Subfamily I	SiGATA20	SiGATA27	0.91	1.64	0.56	960.00	247.83	712.17	375.92	165.08	0.53	0.67
Subfamily I	SiGATA20	SiGATA28	0.82	1.65	0.50	843.00	219.50	623.50	311.58	146.42	0.50	0.67
Subfamily I	SiGATA25	SiGATA26	0.36	0.67	0.54	771.00	205.42	565.58	162.83	91.17	0.29	0.44
Subfamily I	SiGATA25	SiGATA27	0.66	0.80	0.82	696.00	178.58	517.42	226.33	87.67	0.44	0.49
Subfamily I	SiGATA25	SiGATA28	0.73	0.99	0.74	678.00	174.42	503.58	235.00	96.00	0.47	0.55
Subfamily I	SiGATA26	SiGATA27	0.75	1.29	0.58	999.00	263.50	735.50	347.83	162.17	0.47	0.62
Subfamily I	SiGATA26	SiGATA28	0.72	1.00	0.72	855.00	224.83	630.17	292.58	124.42	0.46	0.55
Subfamily I	SiGATA27	SiGATA28	0.64	0.95	0.68	876.00	228.25	647.75	279.33	122.67	0.43	0.54

Subfamily II	SiGATA01	SiGATA05	0.94	1.46	0.64	837.00	208.67	628.33	335.92	134.08	0.53	0.64
Subfamily II	SiGATA01	SiGATA06	0.84	1.62	0.52	678.00	175.17	502.83	253.75	116.25	0.50	0.66
Subfamily II	SiGATA01	SiGATA09	0.44	1.21	0.36	933.00	218.33	714.67	237.00	131.00	0.33	0.60
Subfamily II	SiGATA01	SiGATA11	0.78	1.73	0.45	594.00	141.17	452.83	219.67	95.33	0.49	0.68
Subfamily II	SiGATA01	SiGATA13	0.81	2.25	0.36	561.00	132.75	428.25	211.42	94.58	0.49	0.71
Subfamily II	SiGATA01	SiGATA17	0.76	2.47	0.31	402.00	97.17	304.83	145.83	70.17	0.48	0.72
Subfamily II	SiGATA01	SiGATA22	0.65	1.46	0.44	447.00	110.08	336.92	146.17	70.83	0.43	0.64
Subfamily II	SiGATA05	SiGATA06	0.48	0.66	0.72	645.00	175.75	469.25	165.50	77.50	0.35	0.44
Subfamily II	SiGATA05	SiGATA09	0.91	1.38	0.66	870.00	219.25	650.75	343.58	138.42	0.53	0.63
Subfamily II	SiGATA05	SiGATA11	0.82	2.12	0.39	612.00	158.67	453.33	226.00	112.00	0.50	0.71
Subfamily II	SiGATA05	SiGATA13	1.27	0.90	1.41	585.00	139.83	445.17	272.67	73.33	0.61	0.52
Subfamily II	SiGATA05	SiGATA17	0.82	1.45	0.57	402.00	101.58	300.42	149.83	65.17	0.50	0.64
Subfamily II	SiGATA05	SiGATA22	0.76	0.75	1.01	417.00	109.25	307.75	147.08	51.92	0.48	0.48

Subfamily II	SiGATA06	SiGATA09	0.80	1.26	0.63	681.00	182.25	498.75	244.92	111.08	0.49	0.61
Subfamily II	SiGATA06	SiGATA11	0.98	1.68	0.58	573.00	150.42	422.58	231.17	100.83	0.55	0.67
Subfamily II	SiGATA06	SiGATA13	0.72	0.75	0.95	441.00	108.25	332.75	153.50	51.50	0.46	0.48
Subfamily II	SiGATA06	SiGATA17	0.76	1.05	0.72	384.00	100.75	283.25	135.00	57.00	0.48	0.57
Subfamily II	SiGATA06	SiGATA22	0.75	0.61	1.23	414.00	105.50	308.50	146.08	43.92	0.47	0.42
Subfamily II	SiGATA09	SiGATA11	0.87	1.56	0.56	600.00	142.92	457.08	235.17	93.83	0.51	0.66
Subfamily II	SiGATA09	SiGATA13	0.87	1.01	0.86	579.00	135.00	444.00	228.25	74.75	0.51	0.55
Subfamily II	SiGATA09	SiGATA17	0.71	1.89	0.37	387.00	93.92	293.08	134.25	64.75	0.46	0.69
Subfamily II	SiGATA09	SiGATA22	0.65	0.99	0.66	447.00	109.50	337.50	146.83	60.17	0.44	0.55
Subfamily II	SiGATA11	SiGATA13	0.65	1.61	0.40	399.00	88.25	310.75	134.58	58.42	0.43	0.66
Subfamily II	SiGATA11	SiGATA17	0.34	0.97	0.36	405.00	94.42	310.58	85.67	51.33	0.28	0.54
Subfamily II	SiGATA11	SiGATA22	0.62	1.57	0.39	393.00	91.75	301.25	126.67	60.33	0.42	0.66
Subfamily II	SiGATA13	SiGATA17	0.53	1.76	0.30	405.00	92.75	312.25	118.08	62.92	0.38	0.68

Subfamily II	SiGATA13	SiGATA22	0.27	0.46	0.58	447.00	105.33	341.67	77.58	36.42	0.23	0.35	
Subfamily II	SiGATA17	SiGATA22	0.55	1.12	0.49	399.00	96.25	302.75	118.00	56.00	0.39	0.58	
Subfamily III	SiGATA10	SiGATA21	0.64	2.05	0.31	477.00	117.75	359.25	154.42	82.58	0.43	0.70	
Subfamily III	SiGATA10	SiGATA23	0.58	2.44	0.24	843.00	211.08	631.92	254.83	152.17	0.40	0.72	
Subfamily III	SiGATA10	SiGATA24	0.58	NaN	NaN	939.00	226.92	712.08	287.83	172.17	0.40	0.76	High Sequence Divergence Value (pS>=0.75)
Subfamily III	SiGATA21	SiGATA23	0.75	NaN	NaN	468.00	114.92	353.08	167.25	97.75	0.47	0.85	High Sequence Divergence Value (pS>=0.75)
Subfamily III	SiGATA21	SiGATA24	0.65	NaN	NaN	471.00	116.67	354.33	153.75	105.25	0.43	0.90	High Sequence Divergence Value (pS>=0.75)
Subfamily III	SiGATA23	SiGATA24	0.49	1.49	0.33	870.00	213.58	656.42	235.75	138.25	0.36	0.65	
Subfamily IV	SiGATA14	SiGATA15	0.05	0.15	0.32	741.00	174.25	566.75	25.83	23.17	0.05	0.13	

Additional file 7: Table S7-2. Ka/Ks ratio distribution of tandem duplication and segmental duplication gene pairs.

Type	Gene 1	Gene 2	Ka	Ks	Ka/Ks	EffectiveLen	AverageS-sites	AverageN-sites	cN	cS	pN	pS
tandem duplication	SiGATA25	SiGATA26	0.36	0.61	0.60	765.00	202.50	562.50	161.83	84.17	0.29	0.42
tandem duplication	SiGATA14	SiGATA15	0.05	0.15	0.32	741.00	174.25	566.75	25.83	23.17	0.05	0.13
tandem duplication	SiGATA18	SiGATA19	0.42	0.45	0.92	1023.00	268.67	754.33	240.92	91.08	0.32	0.34
segmental duplication	SiGATA03	SiGATA16	0.24	0.51	0.46	1239.00	331.75	907.25	184.50	123.50	0.20	0.37
segmental duplication	SiGATA11	SiGATA17	0.22	0.74	0.30	378.00	87.58	290.42	55.92	41.08	0.19	0.47
segmental duplication	SiGATA07	SiGATA12	0.16	0.61	0.27	1083.00	282.58	800.42	118.17	117.83	0.15	0.42
segmental duplication	SiGATA01	SiGATA09	0.38	1.07	0.36	915.00	213.17	701.83	210.58	121.42	0.30	0.57
segmental duplication	SiGATA05	SiGATA06	0.46	0.63	0.73	648.00	175.83	472.17	162.08	74.92	0.34	0.43

Additional file 7: Table S7-3. Mean value of *SiGATA* genes Ka/Ks.

Type	Means of Ka/Ks
Subfamily I	0.71
Subfamily II	0.60
Subfamily III	0.29
Subfamily IV	0.32
Tandem duplication	0.61
Segmental duplication	0.42

Table S8. Primer sequences for qRT-PCR.

Supplementary Table S8. Primer sequences for qRT-PCR.

Serial number	Gene primer	Primer sequence(5'to3')
1	SiACTIN-F	GGCAAACAGGGAGAAGATGA
2	SiACTIN-R	GAGGTTGTCGGTAAGGTCACG
3	SiGATA06-F	CGCCAACTGCGACACCACG
4	SiGATA06-F	CGCTCTTCCTTCTTGTACCGGATC
5	SiGATA09-F	CGCCCTTATGGAGGAGTGGT
6	SiGATA09-R	CCTTCGGGTGATGTACTGATGC
7	SiGATA10-F	GGTCATCCTCTGGAGCGTAC
8	SiGATA10-R	CTATTTCATCAGGCTTTGGTTT
9	SiGATA11-F	GTGGGGTTCGGCAAGGAG
10	SiGATA11-R	GAGGGCCATGAGCAGGATG
11	SiGATA12-F	GCCTGGTATGTTCATCCCG
12	SiGATA12-R	GGACGGCTTTGACGGTTTC
13	SiGATA14-F	ACCATAGCGATACTCCTG
14	SiGATA14-R	AGTTGACTGTTGCCTTGT
15	SiGATA15-F	GTGCCATCAAGAAAGAGG
16	SiGATA15-R	CAAAGGAACCCACAGGA
17	SiGATA16-F	CCTCGTCAGGTTCGTTCTCG
18	SiGATA16-R	CGCTTCTTGGGCTTGTGC
19	SiGATA18-F	CGCCAGCAGAGCAAGAACA
20	SiGATA18-R	CCGCACCTGAACCTGAAGC
21	SiGATA22-F	GTGGGGTTCGGCAAGGAG
22	SiGATA22-R	GAGGGCCATGAGCAGGATG
23	SiGATA24-F	TGCCTGGAAACCCGAACC
24	SiGATA24-R	GAATGTCTGTCCTCCGAAGTATGT
25	SiGATA25-F	GATGCACTCCAACCGGCACAAC
26	SiGATA25-R	TCGGAAGAAACCGCTCCCAGA
